# Genome at Juncture of Early Human Migration: A Systematic Analysis of Two Whole Genomes and Thirteen Exomes from Kuwaiti Population Subgroup of Inferred Saudi Arabian Tribe Ancestry

**DOI:** 10.1371/journal.pone.0099069

**Published:** 2014-06-04

**Authors:** Osama Alsmadi, Sumi E. John, Gaurav Thareja, Prashantha Hebbar, Dinu Antony, Kazem Behbehani, Thangavel Alphonse Thanaraj

**Affiliations:** Dasman Diabetes Institute, Dasman, Kuwait; University of Florence, Italy

## Abstract

Population of the State of Kuwait is composed of three genetic subgroups of inferred Persian, Saudi Arabian tribe and Bedouin ancestry. The Saudi Arabian tribe subgroup traces its origin to the Najd region of Saudi Arabia. By sequencing two whole genomes and thirteen exomes from this subgroup at high coverage (>40X), we identify 4,950,724 Single Nucleotide Polymorphisms (SNPs), 515,802 indels and 39,762 structural variations. Of the identified variants, 10,098 (8.3%) exomic SNPs, 139,923 (2.9%) non-exomic SNPs, 5,256 (54.3%) exomic indels, and 374,959 (74.08%) non-exomic indels are ‘novel’. Up to 8,070 (79.9%) of the reported novel biallelic exomic SNPs are seen in low frequency (minor allele frequency <5%). We observe 5,462 known and 1,004 novel potentially deleterious nonsynonymous SNPs. Allele frequencies of common SNPs from the 15 exomes is significantly correlated with those from genotype data of a larger cohort of 48 individuals (Pearson correlation coefficient, 0.91; p <2.2×10^−16^). A set of 2,485 SNPs show significantly different allele frequencies when compared to populations from other continents. Two notable variants having risk alleles in high frequencies in this subgroup are: a nonsynonymous deleterious SNP (rs2108622 [19:g.15990431C>T] from CYP4F2 gene [MIM:*604426]) associated with warfarin dosage levels [MIM:#122700] required to elicit normal anticoagulant response; and a 3′ UTR SNP (rs6151429 [22:g.51063477T>C]) from ARSA gene [MIM:*607574]) associated with Metachromatic Leukodystrophy [MIM:#250100]. Hemoglobin Riyadh variant (identified for the first time in a Saudi Arabian woman) is observed in the exome data. The mitochondrial haplogroup profiles of the 15 individuals are consistent with the haplogroup diversity seen in Saudi Arabian natives, who are believed to have received substantial gene flow from Africa and eastern provenance. We present the first genome resource imperative for designing future genetic studies in Saudi Arabian tribe subgroup. The full-length genome sequences and the identified variants are available at ftp://dgr.dasmaninstitute.org and http://dgr.dasmaninstitute.org/DGR/gb.html.

## Introduction

Genetic approaches, like Whole Genome Sequencing (WGS), Exome Sequencing and Genome Wide Association (GWA) Studies, have helped identify causal variants associated with various recessive and complex disorders in many populations [1]–[4]. The last decade has witnessed sequencing of personal genomes of European, African and Asian descent, including famous personalities such as James Watson, Craig Ventor, and Stephen Quake [5]–[13]. However, the primary resources required for disease association studies are provided by population-scale projects such as 1000 Genomes Project [14] and International HapMap Project [15]. These efforts have enabled creation of imputation panels [16], [17] and detailed catalogues of SNPs, indels and large structural variations [18]. By virtue of considering geographically diverse populations, (for instance, the 1000 Genomes Project that considers 1,092 genomes sampled across 14 populations from Europe, East Asia, sub-Saharan Africa and America) these efforts have also identified rare or population-specific variants in addition to common variants.

Individual populations can exhibit different profiles of rare and common variants [19]. The 1000 Genomes Project, demonstrates that as high as 53% of rare variants (at Minor Allele Frequency (MAF) ≤0.5%) are observed only in a single population, and 17% of low-frequency variants (at MAF of 0.5% to 5%) are observed in a single ancestry group (more than one population form a ancestry group – for example, Africa ancestry group is made up of samples from YRI, LWK and ASW populations). Apart from whole genome sequencing projects, population-scale exome sequencing projects (such as NHLBI Grand Opportunity Exome Sequencing Project (ESP) [20] that covers diverse and richly-phenotyped populations in United States of America) and large-scale exome sequencing projects conducted in individual countries (China [21], Tibet [22], Denmark [23], and Qatar [24]) have also provided a large catalogue of variants and have often been successful in associating rare variants to diseases.

Substantial drop in sequencing costs have enabled large scale sequencing of populations, such as Southeast Asian Malay population (by the Singapore Sequencing Malay Project, SSMP) [25], hitherto not represented in major genome variation studies. These efforts in under-represented populations help in identification of novel and low-frequency variants (including point mutations and structural changes). Low frequency variants, by virtue of being recent in origin, can exhibit increased level of population differentiation and can have profound effects on phenotypes [26].

Arabian Peninsula is at the nexus of Africa, Europe, and Asia and has been implied in early human migration route out of Africa, [27], [28] and in early inter-continental trade routes [29]. The State of Kuwait is situated on the north-east of the Peninsula, and at the northern end of the Persian Gulf. Parts of Kuwait have been inhabited since ancient times. Archeological antiquities discovered in the region point to urban existence dating back to more than four thousand years. This is due to Kuwait's unique geographic location, enabling it to serve as both sea and land links connecting different parts of the Old World [30], [31].Kazima (an ancient name for the region of Kuwait) has served as a stop for caravans coming from Persia and Mesopotamia en route to the eastern and internal parts of the Peninsula, and as commercial link between the countries of Indian Ocean, Syria, and Europe [32], [33]. In Failaka (an island of Kuwait), archaeologists find a complex history of human occupation extending back to 4,000 years [34]. A team from Denmark's Moesgård Museum has unearthed Mesopotamian-style building in Failaka's southwest corner, typical of those seen in the nearby Iraqi mainland, dating back to 2000 BC [35]. It is believed that Dilmunites, a maritime people hailing from today's Bahraini and Saudi Arabian coasts and controlling Persian Gulf trade, inhabited the island in 1800 BC [36]. Between 356 BC and 323 BC, Alexander the Great established a trading post (called Icaros by the Greeks) on the island [37]. The island probably fell under the control of different empires such as the Selucid (remains of Alexander the Great's Macedonian empire), Byzantine (eastern Roman Empire), and Sassanid (Neo-Persian empire) [38]. Later, nomadic Bedouin tribes inhabited the area, and Islamic armies engaged Persian forces at Kazima in 623 AD, beginning their conquest of Persia [39].

Kuwait, like many other countries in the Peninsula, is constituted of settlements from three ancient population groups of (i) Persian origin; (ii) “city-dwelling” Saudi Arabian tribe origin; and (iii) “tent-dwelling” Bedouin origin from Middle East and North Africa. The subgroups in Kuwaiti population resulting from these three distinct ancestries have been characterized in earlier studies using data on mitochondrial DNA genetic variations, Y-STRs, and genome-wide genotypes [40]–[42]. The term “city-dwelling tribes” is used in the context of “true” Arabian tribes, who descended from the ancient tribes of Arabia, which inhabited ancient (Pre-Islamic) cities of Saudi Arabia such as Mada'in Saleh, Al-Shuwayhatiyah, Mecca and Medina [43]. The nomadic Bedouins come from the deserts (Middle East and North Africa) or cultivated areas bordering the deserts [44].

The Kuwait Genome Project (KGP) aims to sequence (at high resolution) genomes from different ethnic groups inhabiting Kuwait. In this work, we report for the first time a genome sequence resource of the Kuwaiti population subgroup of inferred Saudi Arabian tribe ancestry. This subgroup traces origin to the tribes of Najd region of Saudi Arabia. Najd is a plateau in the central region of the Arabian Peninsula spanning from west to east; the eastern sections (historically known as Al-Yamama) are marked by oasis settlements engaged in farming and trading activities, while the rest has been sparsely occupied by nomadic Bedouins). A group of families and tribes from the Najd region began to arrive into the Kuwait region in the year 1613 AD and this marked the formation of modern-day Kuwait. The Bani Khalid tribe established a fishing village (called Qurain) on the site of present day Kuwait City and maintained ties with members of their tribe who had settled and wielded political influence in the region of Najd during their earlier migration eastwards. Since 1710 AD, several other tribes left Najd region (because of drought) and started to arrive (after wandering around the Arabian Peninsula) into Kuwait in several waves until 1756 AD. Bani Utub tribe (comprising the families of al-Sabah, al-Khalifa, and al-Jalahima), one of the new arrivals, took over power from the Bani Khalid rulers [45].

The genome resource is developed by sequencing two whole genomes (referred as KWS1 and KWS2) at >40× coverage, and thirteen exomes (referred as KWS3-15) at >60× coverage. In total, we catalog 4,950,724 SNPs, 515,802 short indels and 39,762 structural variations. We further identify a set of 2,485 exomic markers having significantly different allele frequencies compared to other global populations. This study further provides genetic evidence that the HVS1 (Mitochondrial Hypervariable segment 1) segments of the 15 samples cluster with the observed segments from native Saudi Arabian population [46]; the evidence corroborates our earlier work [42] that 81% of the surnames in Kuwait S group are of Saudi Arabian tribe origin.

## Results

A group of 15 Kuwaiti natives of Saudi Arabian tribe origin, from the Kuwait S subgroup as confirmed in our previous study [42] was selected. The tribe names of the 15 participants and the ancestry estimates are presented in Table S1. The contribution due to Arab ancestry (as deduced using genotype data from Negev Bedouin population as reference – see [42] for details) is at a mean value of 74.4% (ranging between 66.1% and 86.5%); and contributions due to European and West Asian ancestries are at mean values of 10.55% (ranging between 5.8% and 13.4%) and 11.11% (6.2% to 18.5%) respectively. Examination of genetic clusters derived using principal component analysis (PCA) for Kuwait population (Figure S1) reveals that these samples are located deep in the Saudi Arabian tribe cluster (and not at the boundaries of the clusters or in regions that overlap among the three clusters). Whole genome sequencing was carried out on two samples, and exome sequencing on the remaining thirteen. (see Materials and Methods).

### Classification of single nucleotide polymorphisms (SNPs) and insertions/deletions (Indels)

A total of 4,950,724 SNPs and 515,802 indels were identified. These variants were sub-divided into two categories viz. variants that are in exome regions (**UE**, union set of exomic variants from the two whole genomes and thirteen exomes) and the rest (**UW**, union set of non-exomic variants from the two whole genomes). The UE data set includes 121,470 SNPs and 9,675 indels; the UW data set includes 4,829,254 SNPs and 506,127 indels. Of the total reported SNPs, 1,174 (UE: 109; UW: 1065) are found to be triallelic. Biallelic SNPs in the UE data set show a higher genome-wide transition-to-transversion (Ti:Tv) ratio of 2.58, as compared to SNPs in the UW data set, which gives a value of 2.09.

When assessed against dbSNP 137 [47] (which includes variants reported in 1000 Genomes Project phase I release), it is seen that 10,098 (8.3%) SNPs from the UE data set and 139,923 (2.9%) SNPs from the UW data set are either not included in dbSNP or the alleles seen in Kuwaiti samples are not subset of alleles seen in dbSNP; these SNPs can be considered as ‘novel’ variants. A similar analysis on indels identifies 5,256 (54.32%) novel indels from the UE data set and 374,959 (74.08%) from UW data set; the size distribution of the novel biallelic indels is similar to that of the known biallelic indels in both the UE and UW data sets (Figure S2). We observed enrichment of low frequency variants in novel biallelic SNPs from the UE data set (79.98% have minor allele frequency (MAF) <5%) and only a small fraction of the novel SNPs are ‘common’ (5.6% have MAF >10%) (Figure S3). Significant difference is also observed in the mean values of MAF between the known and novel SNPs (t-test p-value <10^−16^ with 95% confidence interval of 0.127–0.129).

### Classification of SNPs and indels based on genome annotation

We characterized the biallelic and triallelic variations from the UE and UW data sets onto thirteen classes based on their genome annotation (see Table 1 for biallelic markers and Table S2 for triallelic markers). 43.47% of the known biallelic SNPs from the UE data set lie in coding regions and a further 41.19% of the SNPs lie in 3′ UTRs. A similar distribution is observed for novel SNPs as well. The extent of discovered exomic SNPs in UTRs is as much as that seen in coding regions (CDS). This is to be expected as the TruSeq exon capture kit (that we use in this study), compared to kits from NimbleGen and Agilent, has a high coverage of UTR targets as much as that of CDS [48]. The ratio of nonsynonymous SNPs to synonymous SNPs is 0.923 for known biallelic SNPs and 1.5 for novel biallelic SNPs from UE data set. Amino acid substitutions are also seen highly correlated between the novel and known nonsynonymous SNPs in UE data set (Mantel test r = 0.917 and p-value <10^−4^ for 9999 replicates).

**Table 1:** Classification of the identified biallelic SNPs based on genome annotation.

Class$	UE_SNP_Known	UE_SNP_Novel	UE_Indel_Known	UE_Indel_Novel	UW_SNP_Known	UW_SNP_Novel	UW_Indel_Known	UW_Indel_Novel
Coding	48377	3988	328	461	819	30	10	30
Coding, Splicing	6	3	0	0	2	0	0	0
Downstream	26	2	3	4	27059	800	1011	2623
Downstream, Upstream	3	1	1	0	834	27	31	57
Intergenic	183	17	4	8	2894110	87303	77233	222130
Intronic	262	25	16	39	1733328	50399	51898	143952
NCExonic	8769	796	342	306	3684	105	75	218
NCSplicing	1	0	1	0	44	4	2	4
Splicing	9	0	7	11	69	4	3	23
3′ UTR	45838	4386	3409	3735	3025	110	118	347
3′ UTR, 5′ UTR	3	1	0	0	11	0	1	1
5′ UTR	7772	851	306	353	883	40	17	51
Upstream	38	4	2	1	24626	839	766	2025

$ , Legends to the class types.

**Coding** - Variant is in the coding exonic region of a protein coding transcript.

**Splicing** - Variant affects a nucleotide that is in a splicing region of a coding transcript.

**Downstream** - Variant is within 1000 bp of the transcript stop site on the 3′ side.

**Upstream** - Variant is within 1000 bp of the transcript start site on the 5′ side.

**Intergenic** - Variant does not interact with any gene transcripts.

**Intronic** - Variant lies within an intron.

**NCSplicing** - Variant affects a nucleotide that is in a splicing region of a non-coding transcript.

**NCExonic** - Variant is in an exon for a non-coding transcript.

**UTR5** - Variant is in an exon of a coding transcript but is on the 5′ side of the start codon.

**UTR3** - Variant is in an exon of a coding transcript but is on the 3′ side of the stop codon.

We observe 5,462 known and 1,004 novel potentially deleterious nonsynonymous SNPs in UE data set. Further, a set of 234 SNPs from the UE data set and 2638 SNPs from the UW data set are associated (as inferred using Ensembl Variation database [49]) with previously known diseases and risk factors such as diabetes and cholesterol levels (Table S3).

### Detection of structural variations seen in the two whole genomes

**Figure 1:**
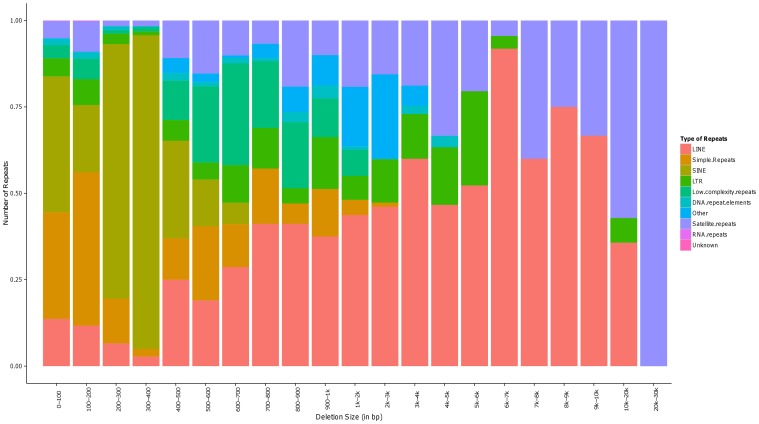
Repeat Composition as seen in deletion variants identified in the two whole genome sequences of Saudi Arabian tribe ancestry. Two regions with highly biased (as compared to general distribution) repeat compositions are seen in the ranges of 300–400 bp and 6–7 kb in length, as insertion polymorphisms of short interspersed nuclear elements (SINE) and long interspersed nuclear elements (LINE), respectively.

Paired-end reads provide a unique opportunity to detect structural variations with high precision. Using algorithms (see Materials and Methods) that survey the configurations of paired-end mappings to detect structural variations, we identify 39,762 variations consisting of 27,060 deletions, 4192 duplications, 1044 insertions, 1137 inversions, 3411 tandem duplicates, 1216 intra-chromosomal translocations, and 1702 inter-chromosomal translocations (Table S4). Of the 27,060 total deletions, 24,351 (89.99%) are “known” structural variations, annotated in DGV (Database of Genomic Variations, a curated catalog of human genomic structural variations) [50]. Further, we see that 15,523 (57.36%) of the total deletion variants lie in repeat-rich regions containing SINE (which include ALU), LINE and LTR repeat elements. We observe two regions with highly biased (compared to general distribution) repeat compositions of length in the ranges 300–400 bp and 6–7 kb, as insertion polymorphisms of SINE and LINE elements respectively (Figure 1).

### Frequency analysis of variants from the UE data set - comparison of allele frequencies between small and large sample sets

**Figure 2:**
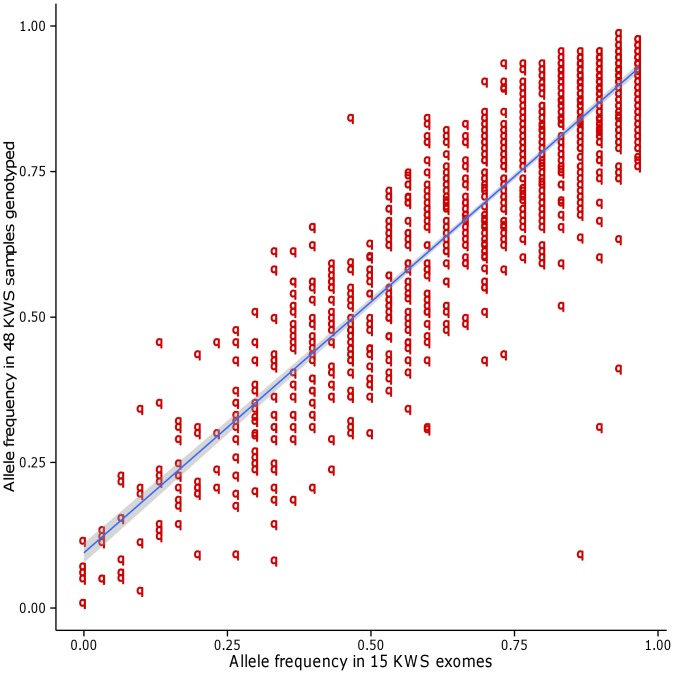
Comparison of allele frequencies of the deleterious nonsynonymous SNPs identified in the exome data set with those in the data set of genotypes from 48 samples.

We considered the remaining set of 48 participants from our previous study [42] which was clustered with the 15 samples sequenced to form the Saudi Arabian tribe subgroup. Allele frequencies were seen correlated (Pearson correlation coefficient 0.90, p<2.2×10^−16^, 95% confidence interval of 0.902 to 0.906) for 25,974 common exomic markers (from UE data set) between the two sample sets. Further narrowing down the comparison to only deleterious SNPs (n = 1,166), the correlation coefficient is still high at a value of 0.91 (p<2.2×10^−16^, 95% confidence interval of 0.899 to 0.919) (Figure 2). These values of 0.90 and 0.91 reflect a strong association implying that allele frequencies determined using a data set of 15 samples hold good in a larger data set of 48 samples. A similar correlation coefficient of 0.89 (95% CI 0.85 to 0.92) has been reported while comparing allele frequencies of 149 (potentially deleterious nonsynonymous) SNPs obtained from exome sequencing of 7 Qatari samples with those obtained from Affymetrix 5.0 microarray genotyping of 149 Qatari samples [51].

### Comparison of genome and exome variability in Saudi Arabian tribe subgroup of Kuwaiti population with global populations

**Figure 3:**
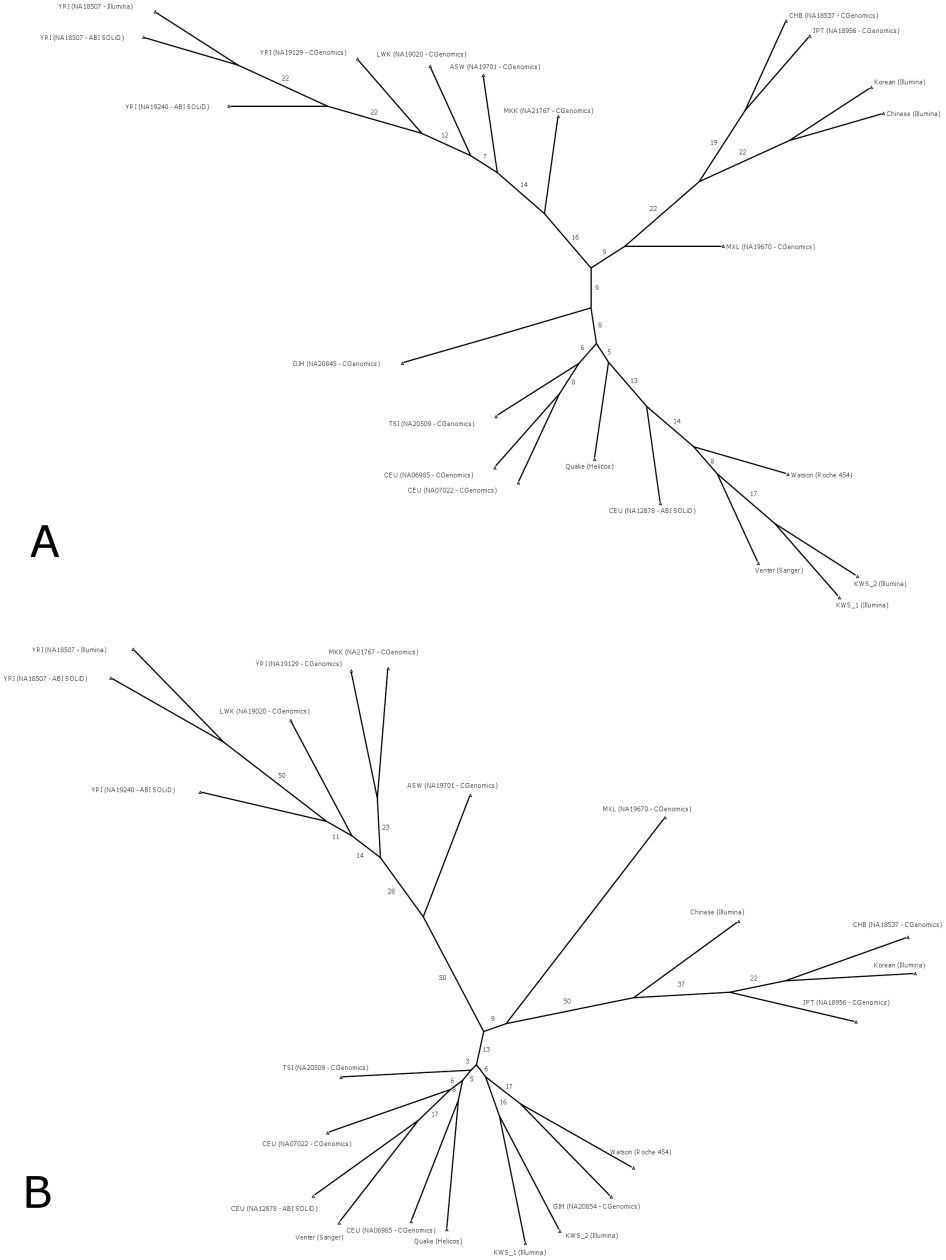
Intergenome distances between the KWS genomes and individuals from continental populations. (a) Nearest neighbor tree based on variant positions shared between the KWS samples and individuals from intercontinental populations. (b). Intergenome comparisons based on variant positions associated with OMIM disease genes and are shared between the KWS samples and individuals from intercontinental populations.

In order to assess the extent of variability exhibited by genomes of Saudi Arabian tribe subgroup, we compare each of the two KWS1 and KWS2 genomes with 20 genomes (see Materials and Methods) across four continents. These 22 genomes have been sequenced using six different technologies: Sanger (1 genome), Roche (1 genome), CGenomics (11 genomes), Helicos (1 genome), ABI SOLID (3 genomes), and Illumina (5 genomes). The intergenome distances among the genomes are calculated by adopting the method of Moore et al. [52]; the method takes care of the variability across the platforms by calculating the extent of shared variant locations. Moore et al. illustrates that neighbor-joining tree constructed using intergenome distances presents ethnicity as the dominant trend and is robust to depth of coverage. The consensus neighbor-joining tree is presented in Figure 3a. The sequences are closely neighbored based on ethnicity. The two KWS genomes are closely neighbored with one another and are placed amidst Europeans. The two groups of Asian and African genomes are placed further away from the two KWS genomes. A similar tree is constructed using the number of shared variant positions with known disease-causing/predisposing alleles as cataloged in OMIM [53] (see Figure 3b).

The overall pattern of neighbor-joining of the genomes using OMIM variants is similar to that seen in the tree deriving using shared genome-wide variants – African genomes are clustered together, the Asian genomes are clustered together, and the KWS genomes are clustered along with European genomes. The one exception observed is that GIH (Gujarati Indians in Houston), that was appearing as a separate branch in the tree based on genome-wide shared variants is now seen clustered along with KWS and Europeans in the tree based on OMIM shared variants. The distribution of known disease-causing and predisposing variants in the Saudi Arabian tribe substructure of the Kuwaiti population is quite distinct from the African and Asian populations, but is similar to those of Europeans and Gujarati Indians (in Houston).

We further compare the allele frequencies of the exomic SNPs with those seen in populations from four continents (AFR, AMR, ASN, and EUR) using data from the 1000 Genomes Project. The Pearson correlation coefficients are seen in the range of 0.73–0.87 (between KWS and AFR: 0.730 [0.727–0.733], KWS and ASN: 0.760 [0.758 - 0.763], KWS and AMR: 0.860 [0.859 - 0.862], and KWS and EUR: 0.872 [0.870 - 0.873]). While the values are modest for KWS with AFR and ASN, they are high for KWS with AMR and EUR. The 1000 Genome AMR is made of three populations namely CLM: Colombian in Medellin, Colombia (60 individuals); PUR: Puerto Rican in Puerto Rico (66 individuals); and MXL: Mexican Ancestry in Los Angeles, California (55 individuals). Examination of the data generated by the 1000 Genomes Project Admixture working group on the proportion of source population ancestry (European, African or Native American) for each individual in each of the three populations of AMR reveals a high European ancestry – the mean value for European ancestry in AMR turns out to be 59.8%
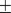
18.8%. Thus it can be inferred that the high correlation with AMR group is due to European ancestry in AMR. In summary, allele frequencies seen in Saudi Arabian tribe substructure of Kuwaiti population are more correlated to those seen in Europeans or admixed Americans with high European ancestry than to Asians or Africans. This result based on exomic SNPs is in agreement with the results from genome comparisons on shared SNPs (Figures 3a and 3b).

**Figure 4:**
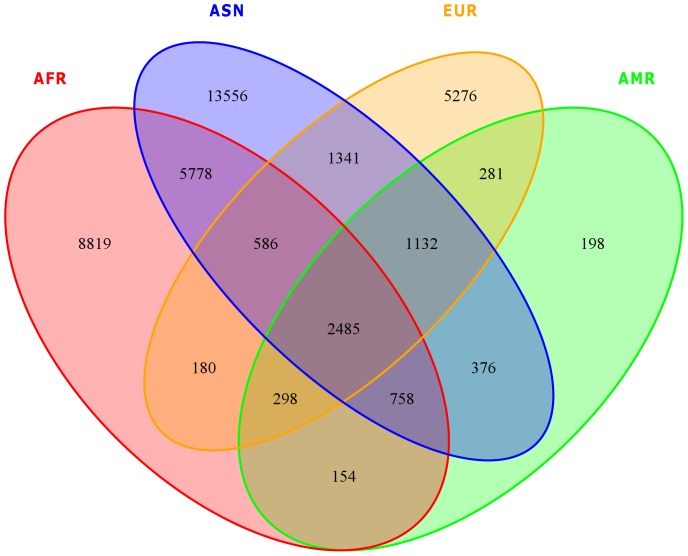
Venn diagram depicting the number of SNPs having significant difference in allele frequencies between the KWS group and other continent populations from the 1000 Genomes Project (F_st_ >0.25 & q-value <0.05).

We identify 2,485 markers (from the UE data set) that show significant differences in allele frequencies when compared with populations from all the four continents (Figure 4). Out of these 2,485 markers, only 164 markers are found to be deleterious (see Supplement Table S5). A set of 1,512 markers lie in OMIM genes. Nine of these markers denote causal variants for OMIM diseases [54]–[63] (see Table 2). It is important to validate the allele frequencies in a larger sample set, which we will consider in our future studies. However, four out of these 9 markers are present in our in-house genotype data set [42] of 63 samples of Saudi Arabian tribe substructure (that includes the 15 samples sequenced in this study). Two out of these four markers still show significant allele frequency differences with the continental populations. These are: rs2108622 (19:g.15990431C>T) (associated with associated with Warfarin drug response [MIM: #122700] and altered Vitamin K (VK1) metabolism [64], [65]) (Risk allele frequencies: AFR: 0.085, AMR; 0.285, ASN: 0.206, EUR: 0.273, KWS: 0.7; larger GWAS data set of Saudi Arabian tribe: 0.532), and rs6151429 (22:g.51063477T>C) (associated with Metachromatic Leukodystrophy [MIM: #250100] [66]], also called Arylsulfatase A deficiency) (Risk allele frequencies: AFR: 0.000, AMR: 0.041, ASN: 0.019, EUR: 0.081, KWS: 0.533; larger GWAS data set of Saudi Arabian tribe: 0.325).

**Table 2:** Markers denoting causal variants for OMIM diseases and showing significant differences in risk allele frequencies between the KWS and continental populations.

SNP_ID (risk allele) & gene name	HGVS	MIM_ID	Phenotype	Risk Allele Frequency					Allele Frequency in a larger data set of 63 Kuwaiti natives of Saudi Arabian tribe ancestry	Reference
				AFR	AMR	ASN	EUR	KWS		
rs1042114 (G)	1:g.29138975G>T	#103780	Alcohol Dependence	0.037	0.086	0	0.131	0.667	0.105	Zhang H et al. [54]
OPRD1 (*165195)										
rs1049254 (G)	16:g.88709828A>G	+608508	Reactive Oxygen Species Generation	0.831	0.646	0.773	0.62	0.133		Bedard K et al. [55]
CYBA (+608508)										
rs1800742 (A)	16:g.2110805G>A	#191100	Tuberous Sclerosis-1	0	0.011	0	0.004	0.133		Jones AC et al. [56], Niida et al. [^57^]
TSC1 (*605284)										
rs1801483 (A)	17:g.79767715G>A	#125853	Diabetes Mellitus, Noninsulin-Dependent	0	0.003	0	0.012	0.133		Hager J et al. [58]
GCGR (*138033)										
rs2020912 (C)	2:g.48027755T>C	#614350	Colorectal Cancer, Hereditary Nonpolyposis, Type 5	0	0	0	0.015	0.167		Wu Y et al. [59]
MSH6 (*600678)										
rs2108622 (T)	19:g.15990431C>T	#122700	Coumarin Resistance/Warfarin resistance	0.085	0.285	0.206	0.273	0.7	0.532	Caldwell MD et al. [60]
CYP4F2 (*604426)										
rs2814778 (C)	1:g.159174683T>C	#110700; #611162	Duffy Blood Group System; protection against Plasmodium Vivax	0.943	0.069	0	0.003	0.4		Reich D et al. [61]
DARC (*613665)										
rs6151429 (C)	22:g.51063477T>C	#250100	Metachromatic Leukodystrophy (also called Arylsulfatase A deficiency)	0	0.041	0.019	0.081	0.533	0.325	Regis S et al. [62]
ARSA (*607574)										
rs7076156 (G)	10:g.64415184A>G	605990	Nephrolithiasis, Uric Acid, Susceptibility To	0.974	0.815	0.913	0.734	0.3	0.508	Gianfrancesco F et al. [63]
ZNF365 (607818)										

### Occurrence of a rare Hemoglobin variant

The most prevalent genetic blood diseases in Kuwait are Thalassemia and Sickle cell anemia [67]. In one out of the 15 samples, we observe a rare hemoglobin variant (Figure S4), called Hemoglobin Riyadh (previously reported, for the first time, in a Saudi Arabian woman from Riyadh, with alpha-thalassemia and iron deficiency [68]). The variant leads to an amino acid substitution (namely, beta120 Lys replaced by Asn) in beta-globin chain. While both lysine and asparagine are polar amino acids, lysine is positively charged and asparagine is neutral. The effect on the function of hemoglobin beta chain is not known.

### Estimates on the number of exomes needed to be sequenced for deriving the complete spectrum of diversity in the tribe population

**Figure 5:**
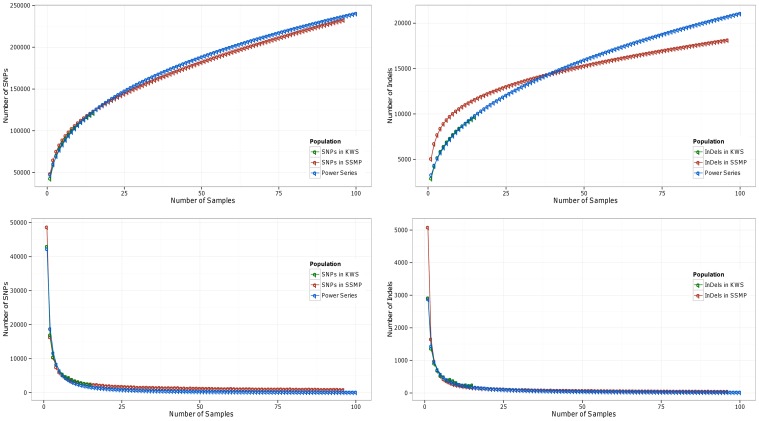
Distribution of total number of variants (SNPs and indels) upon step-wise addition of exomes, and the distribution of number of new variants added per exome. (Coefficient of determination (R^2^) >0.99 for all fitted curves).

The repertoire of variants (SNPs/Indels) increases with the number of samples sequenced. In our study, the number of variants increases from 45,770 (for one exome) to 134,396 (after sequencing 15 exomes). The number of exomic variants we identify in 15 samples is in agreement with that observed in 7 exomes from the population of Qatar (another State from the Peninsula) [51]. It is expected that the increase would saturate when sufficient number of exomes have been sequenced; in order to assess this, we examine the trends in the total number of variants (SNPs and indels) upon step-wise addition of exomes, and the trends in the increase in the number of new variants added per exome (Figure 5). The distribution of the total number of SNPs or indels with every new exome sequenced follows a power series(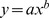). A similar distribution is observed for new variants added per new exome sequenced. The distributions seen are similar to that observed for high coverage sequence data of 100 exomes in South East Asian Malay population [25]. The results indicate that we need to sequence further more samples to capture the full spectrum of diversity in the Saudi Arabian tribe substructure of Kuwaiti population [69].

### Y-chromosome and mitochondrial genetic ancestry

Both the WGS samples (KWS1 and KWS2) are observed to have J1e [J-P58] Y-chromosome haplogroup which is seen in the Arabian Peninsula. The overall estimated time of expansion of J1e haplogroup is around 10,000 years and the ancestors of J1e haplogroups are observed in the Caucasus and eastern Anatolian populations [70].

Furthermore, the mitochondrial haplogroups (indication of maternal ancestry) are determined as J1b2 for KWS1 and H6B for KWS2. Both the haplogroups are observed in Arabian Peninsula, but have different origins. The mitochondrial J haplogroup originated in Near East or Caucasus (a geopolitical region at the border of Europe and Asia, and situated between the Black and the Caspian sea) around 45,000 years from present, whereas the H haplogroup originated in West Asia around 20,000–25,000 years to present and was carried by early human migration to Europe [46]. We also assess the maternal haplogroups in thirteen exomes. The following haplogroups could be identified. **(i) T2c1d1 (2), T2c (1)** - Haplogroup **T** is predominantly of Eurasian lineage. T2b to T2e is found in high frequency in Saudi Arabia; **(ii) U6 (1)**, **U4a1 (1)** – U haplogroup is supposed to have originated 55,000 years back in Eurasia. U6 is seen in 10% of North African population; U4 is seen widely distributed in Europe; **(iii) R0a1a (1), R0a2c (2), R0a1 (1)** – R0 haplogroup is seen frequently in the Arabian belt. Haplogroup R is a common macro-haplogroup seen in West Eurasia; **(iv) H20 (1), H6b (1)** - Haplogroup H is supposed to have originated in Southwest Asia (20–25,000 YBP); H2 and H6 seems to be common in Eastern Europe and the Caucasus; **(v) J1b3b (1), J1b2 (1), J2b2 (1)** - Haplogroup J (along with ‘T’) is associated with people who migrated to Europe and developed farming and herding during the Neolithic Era (8,000–10,000 yrs ago); and **(vi) N1b1a (1) - is** found in Middle East, Egypt, Caucasus and Europe.

**Figure 6:**
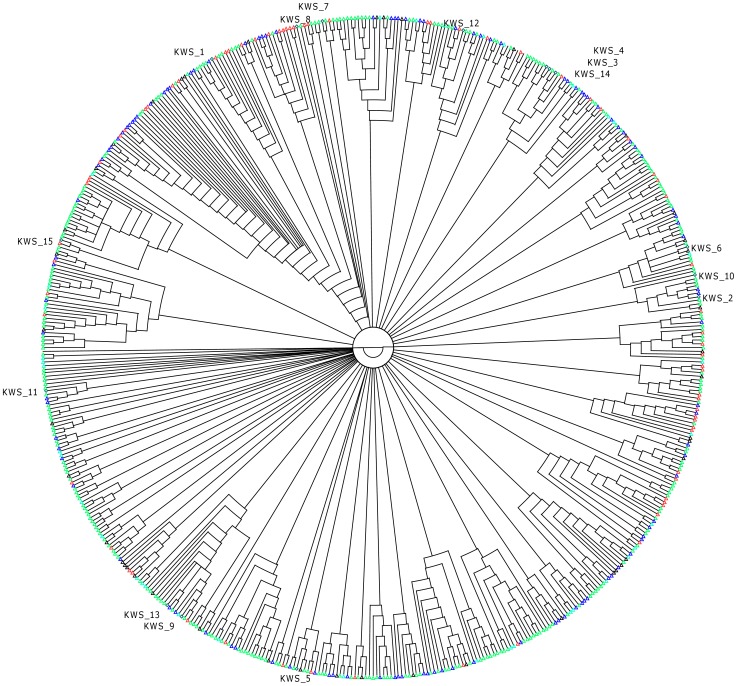
Phylogenetic tree of the observed HVS1 segments among the 15 participants together with those observed by Abu Amero [46] in Saudi Arabia natives. Kuwaiti samples are labeled as KWS. Green triangles denote sample from Central region of Saudi Arabia; Blue triangles denote samples from Southern region of Saudi Arabia; Red triangles denote samples from Western region of Saudi Arabia; Black triangles denote samples from Northern region of Saudi Arabia; Not Known [Cyan triangles].

In an effort to examine the consistency of the observed mitochondrial haplogroups from the 15 participants with those observed in native Saudi Arabian population, we considered the data from Abu-Amero et al. [46]. They analyzed 553 Saudi Arabs using mtDNA sequencing and found a total of 365 different mtDNA haplogroups present in Saudi Arabia. A scattering of haplogroups is expected considering that the strategic location of the Peninsula has enabled it to serve as migration route out of Africa into Eurasia, and as gateway of access for movement of human population across diverse spheres of Asia and Europe for centuries. Neighbor-joining tree (Figure 6), generated by comparing sequence of hypervariable segment (HVS1) among the 15 participants together with those observed in Saudi Arabia indicates that the haplogroups of the 15 KWS samples do not form new branches but rather cluster with the haplogroups (nodes) seen in Saudi Arabia. Thus the mitochondrial haplogroups of the 15 participants (of Saudi Arabian tribe ancestry) considered in this study are consistent with those seen among Saudi Arabian natives.

### Genome view of the variants

**Figure 7:**
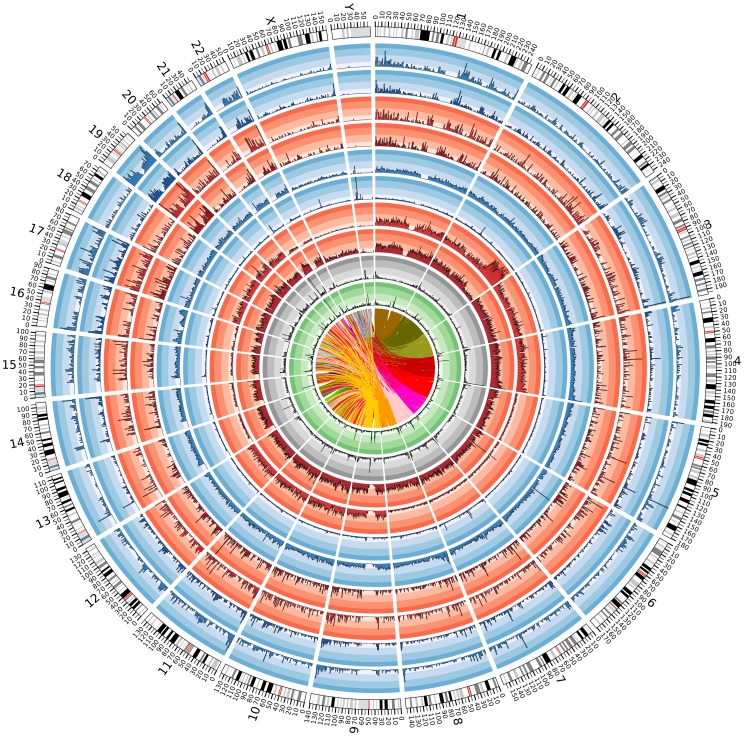
Summary of analysis of genomes from Kuwait subgroup of Saudi Arabian tribe ancestry. Tracks (from outer to inner): Karyotype of Human Genome; Density (in every window of 1 Mb) of ‘known’ SNPs (i.e. annotated in dbSNP 137) from the UE data set; Density of ‘novel’ SNPs (i.e. not annotated in dbSNP137) from the UE data set; Density of ‘known’ indels from the UE data set; Density of ‘novel’ indels from the UE data set; Density of ‘known’ SNPs from the UW data set; Density of ‘novel’ SNPs from the UW data set; Density of ‘known’ indels from the UW data set; Density of ‘novel’ indels from the UW data set; Density of long Indels; Density of duplications, inversions and tandem duplications; Links representing intra- and inter-chromosomal translocations. The image was generated using Circos [71].

Figure 7 provides a high-level view of the contents of the draft genome sequence in terms of density of known and novel variants, (SNPs, short and long indels) observed from whole genome and exome sequences, density of duplications and the extent of chromosomal translocations. We have also created a genome browser (see the section on Data Availability) for users to view an annotated display of the identified variants and structural variations, in the context of sequence and annotation tracks from other genome resources.

## Discussion

The Arabian Peninsula comprises nine countries namely, Bahrain, Iraq, Jordan, Kuwait, Oman, Qatar, Saudi Arabia, United Arab Emirates and Yemen. Genetic clustering using genome-wide genotype data derived from the DNA samples of Kuwaiti natives, followed by observation of concordance with ancestry estimation based on surname lineage classifications, identify a highly endogamous subgroup predominantly of “city-dwelling” Saudi Arabian tribe ancestry among three possible subgroups [42]. In this study, we sequence whole genomes (at coverage >40X) using DNA samples from two participants and exomes (at coverage >60X) from thirteen participants belonging to the “city-dwelling” Saudi Arabian tribe subgroup of Kuwaiti population and identified exomic and non-exomic variants. We report a total of 4,950,724 SNPs (comprising union set of exomic variants from thirteen exomes and the two whole genomes [UE: 121,286 SNPs], and union set of non-exomic variants from 2 whole genomes [UW: 4,762,627 SNPs]), and 496,600 indels (UE: 9,623 and UW: 486,977). Majority of the novel biallelic SNPs from the UE data set are seen in low frequency (79.9% have minor allele frequency (MAF) <5%). Minor allele frequencies of variants derived using exome data from 15 individuals is significantly correlated (Pearson correlation coefficient, 0.90, p<2.2×10^−16^) with those derived using genotype data from a larger cohort of 43 individuals. Furthermore, we identify 39,762 variations consisting of 27,060 deletions, 4192 duplications, 1044 insertions, 1137 inversions, 3411 tandem duplicates, 1216 intra-chromosomal translocations, and 1702 inter-chromosomal translocations. The presented genome characterization (as summarized in Figure 7) provides a unique resource for designing genetic studies targeted at Saudi Arabian tribe subgroup.

The allele frequencies of exomic SNPs from the Saudi Arabian tribe substructure exhibit a higher correlation with Europeans (Pearson correlation coefficient at 0.86–0.87) than with Africans (at 0.73) or Asians (at 0.76). Furthermore, the tribe exhibits a higher degree of intergenome similarlty with ‘Europeans’ than with Africans or Asians, assessed by examining shared genome-wide SNP positions between the genomes; the observation persists even when only those SNP positions that are associated with OMIM disease genes are considered. We further confirm the observation of Moore et al. [52] that ethnicity acts as the dominant trend structuring SNP locations.

We identify a set of 2,485 (out of 103,187) SNPs showing significant differences in allele frequencies with populations from other continents; these variants form Kuwait S (Saudi Arabian tribe) specific variants which can be used for future genetic studies. Two of the variants are particularly interesting:

(**1**) A nonsynoymous deleterious variant (rs2108622 (19:g.15990431C>T), that lies in cytochrome P450 4F2 (CYP4F2 [MIM:*604426]) gene, is associated with Warfarin drug response [MIM:#122700] and altered Vitamin K (VK1) metabolism. The risk allele is shown to be associated with higher Warfarin dosage requirement to elicit anticoagulation response. The risk allele T for this marker is seen in high frequency in KWS samples compared to other continent populations. An earlier study in Kuwait has reported a poor quality of anticoagulation with Warfarin [72]. Thus the rs2108622 variant can be considered as a candidate for clinical characterizations and considered for genetic testing in Kuwaiti population. (**2**) A 3′ UTR variant (rs6151429 [22:g.51063477T>C)]), associated with Metachromatic Leukodystrophy (MLD) [MIM:#250100], lie in ARSA [MIM:*607574] gene. The risk allele C for this marker is seen in high frequency in KWS samples, compared to other continent populations. MLD is an autosomal recessive leukodystrophy, characterized by a buildup of sulfatide fat in cells, especially in cells of the nervous system. The accumulation causes progressive destruction of the myelin sheath, leading to characteristic symptoms that include hypotonia, irritability, gait disturbances, mental deterioration, convulsions, paralysis, spastic tetrapearesis, difficulties in feeding and swallowing, abnormal eye movements, atrophy of the optic nerve, posture abnormalities, ataxia, dementia, and coma. It is estimated that MLD occurs at the rate of one in 40,000 individuals worldwide. However, some populations that include certain Arab groups in the Occupied Territories and a group of Jews who migrated from Southern Arabia (Habbanites) have shown to harbor much higher frequencies of the disease [73]. El Khateeb et al. [74] report that five of the 35 Kuwaiti children (with neurologiocal disorders) examined for auditory brain stem responses demonstrated characteristics of MLD. Instances of the disease have been reported from Oman, another region of the Arabian Peninsula: Koul et al. [75] report the incidence in two siblings with first degree consanguineous parents; and Rajab et al. [76] report the disease in 18 patients with an observed incidence of 1 in 25,000 births during the years 1993 to 2002. Heinisch et al. [77] found it to be more frequent among Arabs living in two restricted areas: of ten families with affected children, 3 from the Jerusalem region and 7 from a small area in lower Galilee. Two of these families were Muslim Arabs and 2 were Christian Arabs. Zlotogora et al. [78] could identify 3 Muslim Arab families and 1 Christian Arab family from Jerusalem to study the disease ARSA haplotype defined by 3 intragenic polymorphic sites; the parents were first cousins in all these 4 unrelated families. They found the same haplotype in 8 non-Arab patients from the US and Europe and postulated that the haplotype has a common origin for the mutation and may have been introduced into Jerusalem at the time of the Crusades.

Hemoglobin Riyadh variant [68] is one of the 1175 hemoglobin variants identified so far [79], [80]. This variant was identified for the first time in a Saudi Arabian woman from Riyadh, and thus it is interesting to find this variant in one of the 15 samples of Kuwaiti Saudi Arabian tribe ancestry. This particular variant has also been subsequently found in members of few Saudi Arabian families, in a family of Mexican-Spanish ancestry, in members of an Asian Indian family, in an 82-year-old Japanese male, and in many other Japanese families. The most prevalent genetic blood diseases in Kuwait are Thalassemia and Sickle cell anemia. The abnormal haemoglobins and the thalassaemias are inherited as autosomal recessive (AR) disorders. Large family size, high rate of consanguinity and tribe/clan endogamy make the Arab region unique from the point of view of genetic disorders. The genes for sickle cell hemoglobin and thalassemia are found in all Arab countries in different frequencies and even within different regions from the same country [81]. While the β thalassemia is prevalent throughout the world, α is found more in the Mediterranean region, Middle East South Asia and South East Asia [82]. Marouf et al. [83] conducted a comprehensive electrophoretic screening of the Kuwaiti population and showed that 23.5 per cent had abnormal hemoglobin genotypes, with beta-thalassemia minor (14%), sickle cell trait (6%), sickle cell anemia (0.9%), S beta zero thalassemia (0.8%) and S beta + thalassemia (0.8%) as most commonly identified hemoglobinopathies; they further identified two rare hemoglobin variants, Hb-D Punjab and Hb-E. Our current work has identified a rare Hemoglobin Riyadh variant in one of the 15 samples analyzed. Another new rare Hemoglobin variant, named as Hb Boston–Kuwait variant, has recently been identified in a Kuwaiti toddler [84]. A systematic characterization of HBB gene and other Hemoglobin genes in Kuwaiti Thalassemia and sickle cell anemia population might further reveal rare ancestry-specific variants of Hemoglobin that are useful in developing prenatal and carrier genetic tests in risk families.

The distribution of total number of SNPs or indels identified with every new exome sequenced, follows a power series (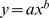), and indicates the need to sequence further samples to capture the genome diversity in the Saudi Arabian tribe substructure. The 2011 census for Kuwait (http://www.e.gov.kw/Documents/English/Forms/CSB/Statistical_Review_2013.pdf) states that the total population of Kuwait is 3.65 million, of which 35% are natives and the remaining expatriates. Per cent distribution of the 1.09 million Kuwaiti natives onto the three subgroups (of Saudi Arabian tribe, Persian, and “tent-dwelling” Bedouin ancestries) is not known. It is to be borne in mind that the current study reports genome variants from just one of these three subgroups and not from the entire Kuwaiti native population. It is expected that the presented repertoire of genome variants will enlarge as we embark upon, in our future works, sampling and sequencing subjects from the other two subgroups in Kuwait.

Both the samples sequenced at whole genome level are observed to have J1e [J-P58] Y-chromosome haplogroup, tracing back their ancestry to Caucasus and eastern Anatolian populations. The maternal haplogroups that the 15 samples belong to are T2c1d1, T2c; U6, U4a1; R0a1a, R0a2c, R0a1; H20, H6b; J1b3b, J1b2, J2b2; and N1b1a. The phylogenetic tree of these mitochondrial haplogroups together with those observed in native Saudi Arabian population by Abu-Amero et al. indicates that the haplogroups of the 15 KWS samples cluster with the haplogroups (nodes) seen in Saudi Arabia. This observation validates phylogenetic consistency and provides genetic evidence to demonstrate the similarity of the Kuwait S subgroup to Saudi Arabian natives. Here, it is worth mentioning that 81% of the surnames in Kuwait S subgroup are of Saudi Arabian tribe origin [42]. In addition, this observation of phylogenetic consistency helps in extending the inference (made by Abu-Amero et al.) of substantial gene flow from Africa and eastern provenance of Arabian Peninsula to the Kuwaiti population. A scattering of haplogroups is seen; such a scattering is expected as populations of the Arabian Peninsula have a complex genetic structure that reflects waves of migrations including the earliest human migrations from Africa and eastern Asia, migrations along ancient civilization trading routes and colonization history of recent centuries. The resultant diversity in mitochondrial haplogroups brings its share of health issues – for example, certain polymorphisms in Haplogroup J are associated with Leber's hereditary optic neuropathy, and the Haplogroup T is associated with increased risk for coronary artery disease and diabetic retinopathy [85], [86].

Listed below are findings (observed in this study) that indicate a certain extent of ancestry affinity between Europeans and the 15 participants from the KWS subgroup: (i) extent of European ancestry admixture in the KWS subgroup is seen at a mean value of 10.55% (see Table S1); (ii) some of the haplogroups identified in the 15 samples correspond to those seen in Europe; (iii) the KWS genomes are seen clustered along with European genomes in the neighbor-joining tree generated using intergenome distances; (iv) allele frequencies of exomic SNPs from the 15 samples exhibit a high correlation with Europeans (Pearson correlation coefficient at 0.86–0.87). The reasons for the observed affinity can be due to various events in the human history. Centrally located among three continents, the geography of the Peninsula (and Kuwait) has contributed to migration influx at different times such as the early human migrations out of Africa into Europe and Asia, migrations along ancient civilization trading routes and colonization history of recent centuries. ***From the perspective of migration outflux into Europe***: Recent reports, based on analysis of ancient European genomes, suggest that one of the three groups to which the present-day Europeans trace their ancestry is Middle Eastern farmers, who are thought to be descendents of humans who inhabited 100,000–120,000-year-old settlements in Israel and the Arabian Peninsula [87]–[88]. The three groups are: hunter-gatherers who arrived from Africa more than 40,000 years ago, Middle Eastern farmers who migrated to the west much more recently, and a third group whose range probably spanned between northern Europe and Siberia. The ancestry admixture due to Middle Eastern farmers in European ancestry may account, at least partially, to the affinity that we see between Europeans and the KWS participants. ***From the perspective of colonization history***: In 3^rd^ century, the ancient Greeks colonized the Bay of Kuwait under Alexander the Great; remains of Greek colonization excavated in Failaka (the island of Kuwait) include a large Hellenistic fort and two Greek temples [37]. States such as Kuwait, Qatar, Bahrain, and the United Arab Emirates are parts of a region that had been under British military and naval “protection” from the year of 1830 onward. Kuwait's initial contacts with the British were primarily through contact with the English East India Company, which was established around 1790 AD; Kuwait served as one of deep water ports in the route between India and Britain (& Europe) for the East India Company.

In conclusion, international efforts such as the HapMap and the 1000 Genomes projects, have considered genomes from continents that include Africa, Asia, Europe and North America, but the Arabian genome remains unexplored. Until now, genomic data of populations from Arabian Peninsula are poorly represented in databases, despite the implication for the study of the early human migrations out of Africa. The region is also a hot spot for the application of medical genetics [89]: the region has high prevalence of genetic disorders (at least partly due to the practice of consanguineous marriages and tribe/clan endogamy) and has seen a high incidence of lifestyle disorders due to rapid nutrition transition in the post-oil era. We report, for the first time, the genomes and exomes of Kuwaiti subgroup of inferred Saudi Arabian tribe ancestry obtained through next generation sequencing of two whole genomes and thirteen exomes at high coverage. Data of the two whole genome sequences and the identified variants is made publicly available to enable global genome comparisons for a better understanding of rare genetic disorders, and of genealogy and migration histories.

## Materials and Methods

### Ethics Statement

The study was approved by the Scientific Advisory Board and the Ethics Advisory Committee at Dasman Diabetes Institute, Kuwait. Written informed consents for the study were obtained from participants before blood samples were collected.

### Participant recruitment and sample collection

A group of 15 participants, belonging to genetically clustered Kuwait S group (as confirmed in our previous study [42]), were considered for sequencing. Two self-declared healthy male participants were selected for whole genome sequencing and the remaining participants were selected for exome sequencing. The participants for exome sequencing include males and females with varying disease phenotypes such as obesity, diabetes and hypertension. Delineation of ancestry elements in each of the three Kuwaiti groups and derivation of the ancestry estimates for each of the participants are carried out using model-based clustering approach (as implemented in STRUCTURE [90]), described in our previous study [42]. The ancestry estimates for the 15 samples sequenced are as extracted from this previous study.

Blood samples were collected in EDTA 4 ml tubes. Gentra Puregene kit (Qiagen, Valencia, CA, USA) was used to extract DNA as per manufacturer's protocols. DNA was quantified, with a requirement that the A260/A280 ratio is in the range of 1.8–2.1, using both the Quant-iT PicoGreen dsDNA Assay Kit (Life Technologies, NY, USA) and the Epoch Microplate Spectrophotometer. Frozen DNA stocks were diluted to a working solution of 50 ng/µl as recommended by Illumina (Illumina, CA, USA).

### Preparation of libraries for whole genome sequencing

Prior to library preparations, DNA was qualified by agarose gel analysis. DNA samples were sheared using Covaris E220 instrument (Covaris, Woburn, MA, USA) which delivers controlled and highly focused non-linear acoustic shock waves into each sample (with the parameters of Duty Cycle 10%; Intensity 4; Cycles per Burst 200;Time 55 seconds) allowing generation of uniformly sized fragments averaging at 400 base pairs in length. Sheared DNA was subsequently used to prepare sequencing libraries; the recommended protocols by the manufacturers (Illumina, CA, USA) for TruSeq DNA sample preparation and cBot Paired End (PE) cluster generation kits were adopted. Precise library concentration and base pair size were validated using Qubit 2.0 Fluorometer (Invitrogen, Life Technologies, USA) and the Bioanalyzer (Agilent Technologies, Inc. USA), respectively, to obtain adequate clustering density on the flow cells. Libraries were then normalized, for size and for optimal loading concentration on the Illumina sequencing flow cells, and for cluster generation using the cBOT. Libraries were loaded on separate lanes on the flow cells for cluster generation on the cBOT, at a final concentration of 12 pmol. Flow cells were loaded on the HiSeq 2000 for paired-end sequencing (with the setting that at least 80% of the called bases meet a quality score of ≥30) using the TruSeq SBS 200 cycles chemistry.

### Exome sequencing

Exome sequencing libraries were prepared using Illumina's TruSeq DNA Sample Preparation Kit. DNA was fragmented (200–300 bp) using Covaris E220 instrument (as mentioned earlier), and the sheared fragments were ligated to the sequencing adaptor oligonucleotides (Illumina Inc, USA). The adaptor-ligated fragments were amplified by PCR cycling and then enriched using the Illumina TruSeq Exome Enrichment Kit (Illumina Inc, USA) which covers 1.22% of human genomic regions corresponding to the CDS (coding sequence) exons. The TruSeq kit adopts an in-solution sequence capture method for isolating exomic regions of interest in the human genome using hybrid selection. Subsequently the ligated fragments were captured by streptavidin-coated magnetic beads and were quantified. Libraries were then clustered on the flow cell at a density of 15 pmol using the cBot as before, and finally were sequenced by the Hiseq2000 using the paired-end reads protocol of 2×100 cycles.

### Image analysis and alignment of reads from whole genome and exome sequencing

We used CASAVA (Consensus Assessment of Sequence And VAriation) v1.8.2 (Illumina Inc, USA) for demultiplexing and Bcl conversion. Sequenced paired-end reads in FASTQ format were aligned to human reference genome hg19 (UCSC) [91] using BWA v0.6.2 [92]. Default parameters were used with the exception of “–q 30 -t 20” for aln command, which allows trimming the reads at the 3′ ends and performs multithreading functionality across the 20 CPU cores of the computer system. The resulting SAM files were converted to BAM format using Sequence Alignment/Map (SAM) tools v0.1.18 [93]. We used Picard v1.86 (http://picard.sourceforge.net) to sort and index the input BAM files. Multiple files from a sample from different HiSeq runs were merged using MergeSamFiles functionality of Picard; the detailed alignment statistics for the 15 samples are presented in Table S6. The visualization of alignments was done using GenomeBrowse™ v1.1 by Golden Helix, Inc.

### SNP and Indel discovery

We used HugeSeq [94], a modular computational pipeline which automates and standardizes the variant discovery process. We modified the pipeline to parallelize the whole process by efficiently splitting the alignment file chromosome-wise into 24 files (22 autosomes +2 sex chromosomes). Alignment files were processed further with Genome Analysis Toolkit (GATK) v2.4-7-g5e89f01 before variant calling [95]. Duplicate removal, local realignment around known indels and base quality recalibration were performed. Details of these steps are as provided below: Potential PCR duplicates were removed by using the tool MarkDuplicates from GATK [96]. RealignerTargetCreator was used to emit intervals for IndelRealigner to target for realignment. For post realignment of indels, we used FixMateInformation tool from Picard to ensure that all mate-pair information is in sync between each read and its mate-pair. BaseRecalibrator tool from GATK then runs a by-locus traversal at sites that are not present in dbSNP v137 [47]. It generates a recalibration table based on several user-specified covariates such as read group, reported quality score, machine cycle and nucleotide context. PrintReads was used to create a recalibrated BAM file.

We used both SAMtools and GATK for variant calling. For the workflow with SAMtools, we used the SAMtools mpileup module in conjunction with Bcftools to generate variant calls. The vcfutils.pl script of SAMtools was used with the D option set to 4000 denoting maximum read depth to call a SNP. For variant calling with GATK, we used UnifiedGenotyper followed by VariantFiltration. In UnifiedGenotyper, we set dcov (downsampling coverage value) to 1000, stand_call_conf (minimum phred-scaled confidence threshold at which variants should be called) to 30, stand_emit_conf (minimum phred-scaled confidence threshold at which variants should be emitted) to 10, glm (Genotype likelihoods calculation model) to BOTH (calls both SNP and indel). We also specified A (annotations to apply to variant calls) as value for the parameters of AlleleBalance, BaseCounts and VariantType. We split variants into separate files for SNPs and indels. VariantFiltration was used to filter variant calls based on certain specific criteria. For SNPs, we set clusterSize to 8, QD<2.0, MQ<40, FS>60, HaplotypeScore >13, MQRankSum <−12.5, and ReadsPosRankSum <−8. For indels, we used the following parameters: clusterSize to 8, QD<2.0, FS>200, and ReadsPosRankSum <−20.

To reduce the likelihood of false discoveries due to the choice of the variant caller, we only utilized the consensus set of variants identified by both SAMtools and GATK workflows in all subsequent analysis. We used VCFtools v0.1.10 [97] to create VCF file (for each of the samples) listing the variants seen from both the workflows of SAMtools and GATK. The variants called in sequenced exomes were filtered to remove those from regions that are not covered by Illumina TruSeq Exome Enrichment Kit.

### Validation of SNP calls

The validity of the SNP calls was confirmed by utilizing the genotype data from the same samples derived using the Illumina HumanOmniExpress BeadChip (Illumina Inc, USA). The concordance rate of the SNP calls between the deep sequencing experiments and the genome-wide genotyping is >99.8% for whole genomes and >99.20% for exomes (Table S7). The observed concordance rates in our study is on par with reported genotype concordance rates in literature: Kenna et al. [98] reports a genotype concordance rate of 98.9% on comparing the accuracy of genotypes inferred for 567 samples across 85 variants using Illumina highthroughput sequencing platforms with genotypes ascertained using Illumina BeadChips. Furthermore, upon comparing a total of 98,113,070 genotypes in 530 individuals called using Illumina HumanExome BeadChip with whole exome sequence data, Grove et al. [99] report a concordance rate of 99.77%.

The discordance in SNP calls is seen in a small number of cases (KWS1: 193 out of 311152; and KWS2: 387 out of 309379). The disagreements in the SNP calls are seen more often with heterozygous SNPs than with homozygous SNPs in both exomes and whole genomes. As is the practice [51], we choose not to remove the inconsistent calls.

### Annotation of variants

We used the SNP & Variation Suite v7.7 (SVS) [100] to annotate the variants with reference to dbSNP 137 database; a variant is denoted as “novel” if either the variant is not annotated in dbSNP or the alternate alleles seen in the variant in the Kuwaiti samples are not a subset of alleles reported in dbSNP. We used Golden Helix software to classify variants according to their genomic position as coding, intronic etc. We further sub-classified coding variants into nonsynonymous, synonymous, gain of stop codon, loss of stop codon etc. The nonsynonymous variants were examined using SIFT [101] and PolyPhen2 [102] and were annotated as “potentially deleterious” based on the predicted possible impact of amino acid substitution (SIFT: Damaging OR PolyPhen2: Probably Damaging). Use of “OR” instead of “AND” was adopted here to maximize the number of deleterious variants [51] reported for future investigation. OMIM track was used to annotate variants for association with diseases. The SNPs were filtered using SVS GWAS track [103] and then annotated using Ensembl Variation database v72 for known human conditions [49].

### Detecting structural variations

We used four different algorithms implemented in HugeSeq pipeline, to detect structural variations from paired-end reads data. BreakDancer version 1.1 [104] was used for paired-end mapping. This method uses anonymously long or short span size between the paired-end reads to identify indels. Furthermore, it identifies both intra- and inter-chromosomal translocations. Pindel version0.2.4 [105] was used for split-read analysis. CNVnator version 0.2.7 [106] was used for read-depth analysis. BreakSeq Lite version 1.0 [107] was used for junction mapping. Calls from the different tools were merged using BEDTools if the reciprocator overlap between two variants is ≥0.5. Deletions were annotated using Annovar [108]. A detected deletion is defined to be ‘known’ if at least 50% of the detected deletion overlaps with annotated deletions in the Database of Genomic Variants [109]; otherwise, the deletion is considered to be “novel”. Repeat content of the identified deletions are estimated using rmsk database from UCSC [110].

### Correlating the allele frequencies of the identified deleterious SNPs with larger data set

We compared the allele frequencies of deleterious SNPs (predicted using SIFT score) that are seen in both the 15 exomes data set and genome-wide genotype data set from a larger number of samples. The allele frequencies were independently calculated in each of these data sets using SVS package; regression analysis was performed using the R package [111].

### Calculation of Intergenome distances between genomes of Saudi Arabian tribe substructure of Kuwaiti population and representative genomes from continental populations, and depiction of consensus neighbor-joining tree

We consider a total of 20 genomes (see Table S8), covering diverse ethnicities (African, Asian, European, and American), downloaded from the sites of 10Gen [52] and Complete Genomics diversity data [12] for comparing the intergenome similarities with the two genomes sequenced in our study.

We adopt the methods used by Moore et al. [52] to calculate intergenome distances based on information relating to shared variant locations between genomes, and to create a consensus neighbor-joining tree depicting intergenome similarities. The intergenome distance is based on location of variants rather than the nucleotide of the variant. The method to calculate the distance is robust with respect to the depth of coverage and hence works well with genomes even when they are sequenced using different sequencing technologies.

The distance metric is calculated as below: *D_i_j* =  (*N*_s_ - [*N*_s_ ∩_ *N*_L_])/*N*_s_, where *D_ij_* is the total intergenome distance, *N*_s_ is the total number of variants in the genome (*i* or *j*) having the fewest number of variants; *N*_L_ is the total number of variants in the genome (*i* or *j*) having the greatest number of variants; and *N*_s_ ∩ *N*_L_ denotes the number of variants in the intersection of the two genomes.

The distance was calculated for every (i,j) pair in the data set of 22 genomes (the two KWS genomes, and the 20 genomes representing Asian, African, and Europeans) on a chromosome-by-chromosome (considering only the 22 autosomes) basis. The resulting 22 distance matrices were then used to produce a neighbor-joining tree (using PHYLIP [112] with default parameters) for each of the 22 chromosomes. PHYLIP Consense was then used to produce the consensus tree. Each of the branches in the resulting tree was annotated by the number of autosomes for which the node was seen.

### Depiction of consensus neighbor-joining tree based on shared OMIM alleles

The tree depicting OMIM phylogenetic comparison is constructed using the same method (as above) adopted to construct the neighbor-joining tree using shared genome variants, but restricting the shared variant locations between genomes to only those locations where one of the genomes contain an OMIM allele. The tree was bootstrapped 50 times, and labels on nodes are the resulting bootstraps.

### Identifying SNPs with significant differences in allele frequencies between the considered cohort and other continent populations

The frequencies of the identified SNPs were compared with allele frequencies seen in 4 continent populations (Europe, Asia, Africa and America) from 1000 Genomes Project (ftp://ftp-trace.ncbi.nih.gov/1000genomes/ftp/release/20110521/) [14]. For each of the SNPs that are seen in both the data sets (the UE data set and the 1000 Genomes Project), two approaches were used to determine whether the frequency of the alternate allele is higher or lower than the continent populations. The allele seen in the UE data set was fixed as alternate allele. Single SNP Fst distances were calculated [113]. A binomial null sampling was assumed with parameter p equal to the continent allele frequencies from the 1000 Genomes Project. A p-value was calculated to assess the significance using two one-sided tests to discover the extreme allele frequencies in both ends of the spectrum. The p-values were FDR (False Discovery Rate) corrected for multiple testing (q-value) [114]. The combined approach was used to assess 103,187 SNPs (see the section on Data Availability), where a threshold of Fst ≥0.25 and q-value of ≤0.05 were applied to identify significantly different markers [51].

### Estimates for number of exomes needed to account for the complete spectrum of diversity in the tribe population

The data from the 100 Malay Project [25] was filtered for exomic SNPs/Indels. 48 random orders of sample merging were used to calculate error bars for both the UE data set and the data set of filtered variants from the Malay Project. The power series fitting was done using the R package.

### Mitochondrial Haplogroup Analysis

The paired-end reads aligned to hg19 mitochondrial sequence were realigned to rCRS (Revised Cambridge Reference Sequence [115] using all the quality control steps that we used for calling variants. The variants were used to call haplogroups using HaploGrep software [116]. The data conversion from VCF file to HaploGrep input file (.hsd file) was performed manually.

### Depiction of consensus neighbor-joining tree based on mitochondrial HVS1 region

Hypervariable segment (HVS1) sequence of 553 natives Saudi Arabia was generated using data provided by Abu-Amero et al. [46]. These along with sequences of HVS1 from 15 participants from this study were used to generate 100 bootstrap samples using seqboot program in PHYLIP package [112]. Dnadist program (from PHYLIP) was used to calculate distance matrices for 100 bootstrap samples. Neighbor program (from PHYLIP) was used to build 100 neighbor-joining trees. Consense program (from PHYLIP) was used to make consensus tree depicted in figure 6.

### Y-chromosome Haplogroup Analysis

The Y-chromosome variants were used to call haplogroups using AMY-tree software [117]. AMY-tree uses data from ISOGG (International Society of Genetic Genealogy). Haplogroups were assigned by using the Y-DNA phylogenetic chart from the resource of The Y-DNA Haplogroup Tree 2013 (Version: 8.59) (http://www.isogg.org/tree/) [Date of access: 7 July 2013]).

### Building the genome browser for visualizing the reference genome sequence

Genome browser is an effective means to share genome data to biomedical community; we have set up JBrowse (version 1.8.1), a graphical interface to enable access to the two reported whole genome sequences for the Saudi Arabian tribe subgroup of Kuwaiti population. JBrowse is an open-source project for genome browser [118]. We have facilitated visualization of external data (such as genome variants reported in DGV [50] and dbSNP [47]) along with the genomes from the two KWS individuals.

### Data Availability

The exomic SNPs and indels (known and novel) identified from the UE data set of the 15 samples are presented as supporting information online (Dataset S1 and Dataset S2, respectively). The non-exomic novel SNPs identified from the UW data set of the two whole genomes are presented as Dataset S3. The non-exomic indels (known and novel) identified from the two whole genomes are presented in Dataset S4. Further, the reported whole genome sequences of two samples for the Saudi Arabian tribe subgroup of Kuwaiti population and all the identified variants (known and novel) from both the UE and UW data sets are available on the ftp site (ftp://dgr.dasmaninstitute.org). The data can be visualized using genome browser with other annotations tracks from UCSC at http://dgr.dasmaninstitute.org/DGR/gb.html. Proper functionality of the web server requires Firefox version 6 (or later versions) or Internet Explorer version 10 (or later versions). Comparison of allele frequencies between the KWS data and continental populations from 1000 Genomes Project is also available at the above ftp site.

## Supporting Information

Figure S1 **Scatter plot representing the first two principal components of merged data sets of the three Kuwaiti groups.** The 15 samples considered for sequencing in this study are color-coded.
(TIF)

Figure S2 **Size distribution of biallelic indels from the UE and UW data sets.**
(TIF)

Figure S3 **Distribution of the observed (known and novel) exomic SNPs (from the 15 samples) as per minor allele frequencies.**
(TIF)

Figure S4 **Alignment of reads denoting the HBB gene fragment containing the SNP corresponding to the Hemoglobin Riyadh variant.**
(TIF)

Table S1 **Phenotype details on the 15 the samples sequenced.**
(DOCX)

Table S2 **Classification of triallelic SNP variants from the UE and UW data set.**
(PDF)

Table S3 **List of SNPs associated (GWAS-linked) with diseases and risk factors.**
(PDF)

Table S4 **Structural variations as seen in the two whole genome sequences of Saudi Arabian tribe ancestry.**
(PDF)

Table S5 **List of the 164 deleterious variants that show significant differences in allele frequencies between KWS samples and continental populations.**
(PDF)

Table S6 **Alignment statistics and genome coverage for the KWS1 and KWS2 (whole genome) samples.** For calculating the percentage of genome covered, the length of human genome is considered as 3,000,000,000 bps (for WGS) and 62,000,000 (for exomes).
(GIF)

Table S7 **Concordance rates for SNP calls between deep sequencing experiments and genome-wide genotyping.**
(PDF)

Table S8 **Details about genome sequences used to compare diversity of KWS genomes.**
(PDF)

Dataset S1 **Data set of all identified exomic SNPs (known and novel) from the UE data set of the 15 samples.** The data is presented in Variant Call Format (VCF).
(GZ) Click here for additional data file.

Dataset S2 **Data set of all identified exomic indels (known and novel) from the UE data set of the 15 samples.** The data is presented in Variant Call Format (VCF).
(GZ) Click here for additional data file.

Dataset S3 **Data set of identified novel non-exomic SNPs from the UW data set of the two whole genomes.** The data is presented in Variant Call Format (VCF).
(GZ) Click here for additional data file.

Dataset S4 **Data set of all identified non-exomic indels (known and novel) from the UW data set of the two whole genomes.** The data is presented in Variant Call Format (VCF).
(GZ) Click here for additional data file.
