# Correction: Genome at Juncture of Early Human Migration: A Systematic Analysis of Two Whole Genomes and Thirteen Exomes from Kuwaiti Population Subgroup of Inferred Saudi Arabian Tribe Ancestry

**DOI:** 10.1371/journal.pone.0103691

**Published:** 2014-07-21

**Authors:** The PLOS ONE Staff

## Notice of Republication

This article was republished on July 3rd, 2014, due to the publishing of an incorrect version of Figure 3 and the release of confidential information. Additionally, formatting errors were corrected in the “Annotation of variants” paragraph of the Materials and Methods section in the PDF version of the article. Please download the PDF again to view the corrected article. The originally published, uncorrected article and the republished, corrected article are provided here for reference.

## Supporting Information

File S1 Originally published, uncorrected article.
(PDF)

File S2 Republished, corrected article.
(PDF)
