# Supplementary figures and images for: Genome at Juncture of Early Human Migration: A Systematic Analysis of Two Whole Genomes and Thirteen Exomes from Kuwaiti Population Subgroup of Inferred Saudi Arabian Tribe Ancestry
Source: PLoS One. 2014 Jun 4;9(6):e99069. doi: 10.1371/journal.pone.0099069 (PMC4045902; doi:10.1371/journal.pone.0099069)

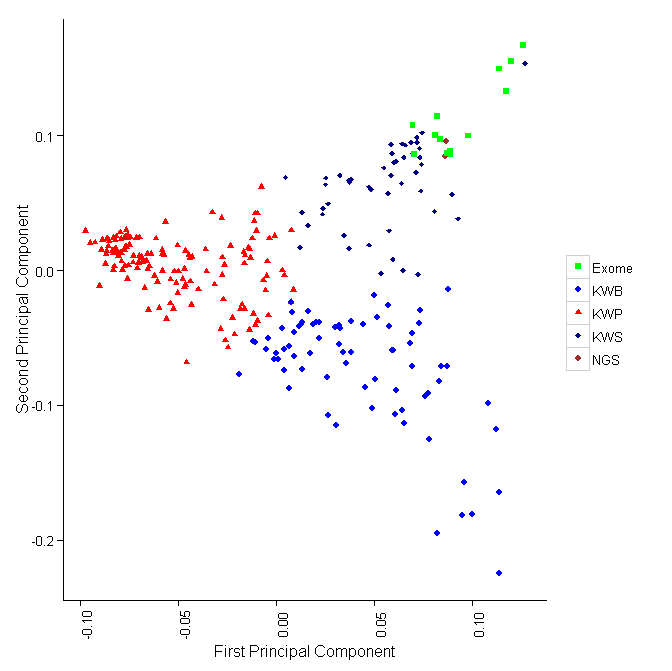

Supplement: Figure S1 — Scatter plot representing the first two principal components of merged data sets of the three Kuwaiti groups. The 15 samples considered for sequencing in this study are color-coded. (TIF) [file pone.0099069.s001.tif]

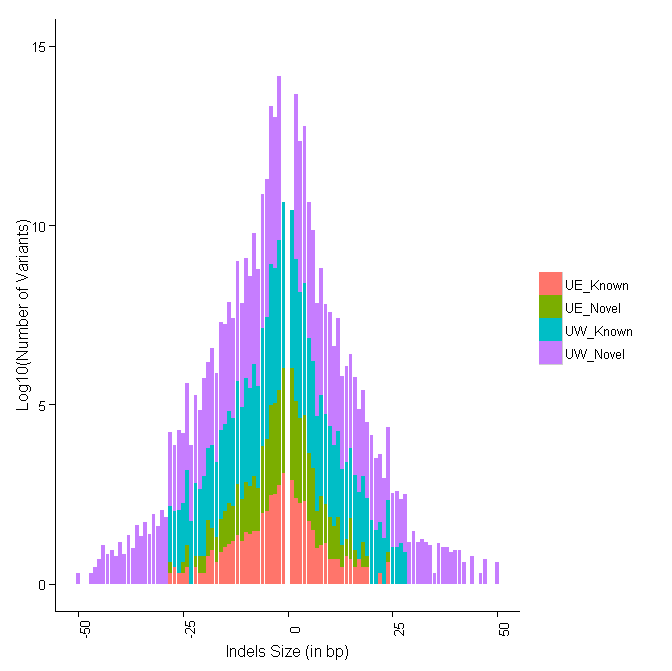

Supplement: Figure S2 — Size distribution of biallelic indels from the UE and UW data sets. (TIF) [file pone.0099069.s002.tif]

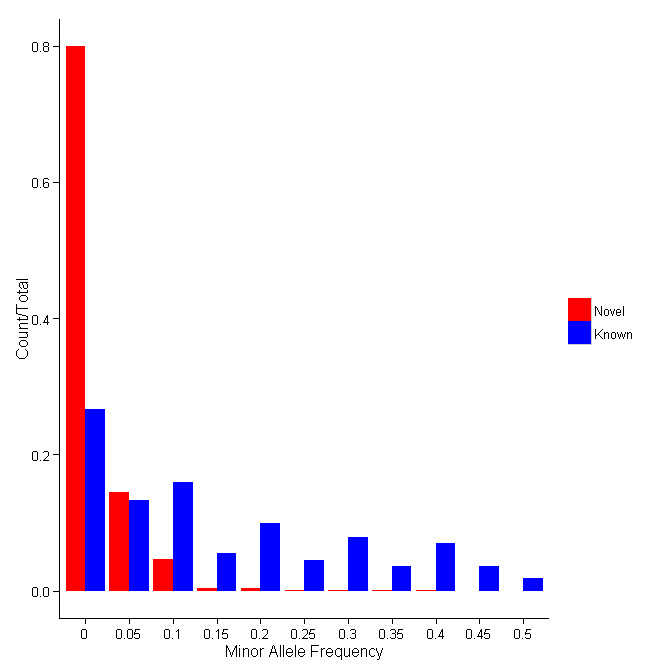

Supplement: Figure S3 — Distribution of the observed (known and novel) exomic SNPs (from the 15 samples) as per minor allele frequencies. (TIF) [file pone.0099069.s003.tif]

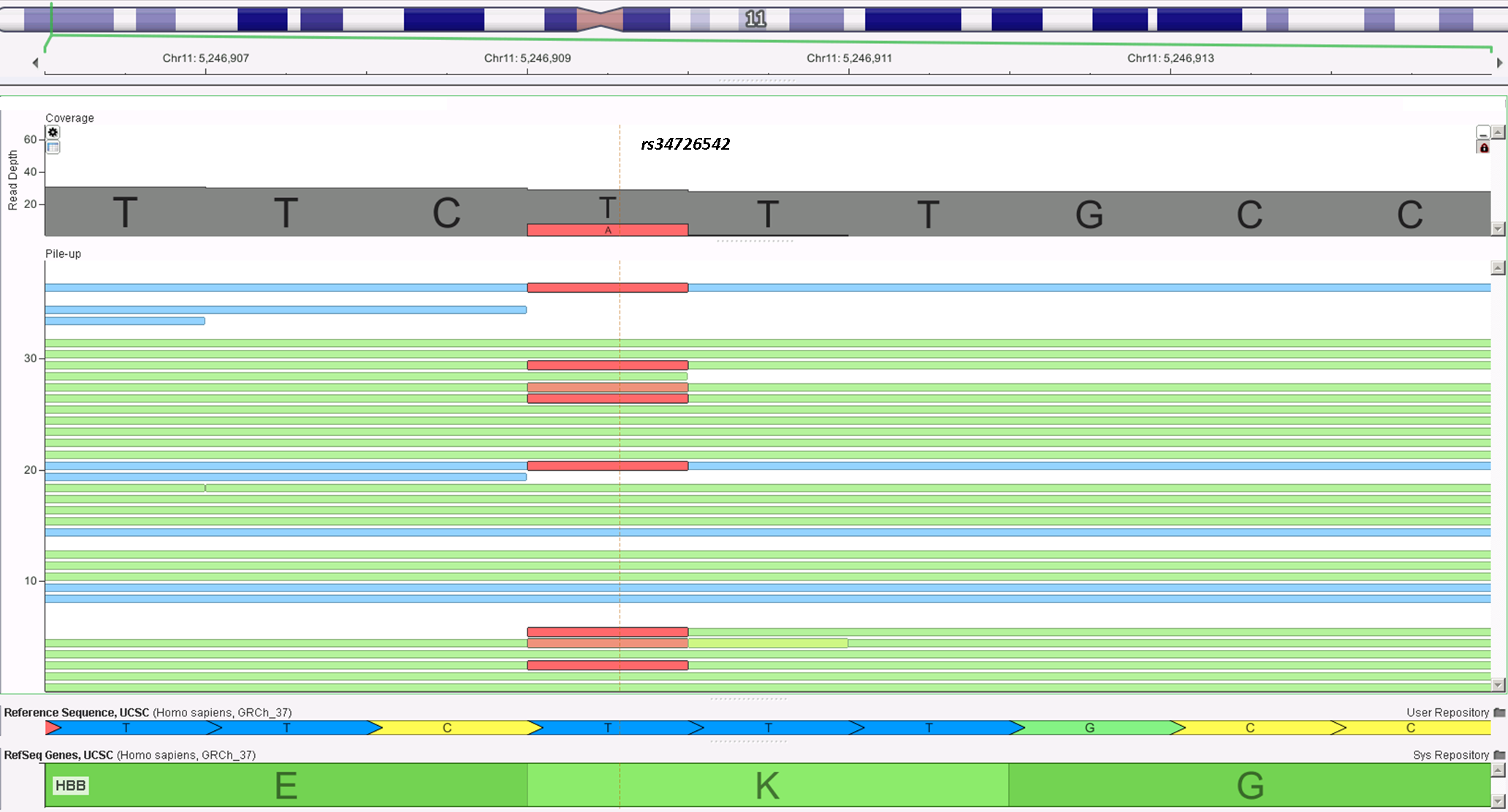

Supplement: Figure S4 — Alignment of reads denoting the HBB gene fragment containing the SNP corresponding to the Hemoglobin Riyadh variant. (TIF) [file pone.0099069.s004.tif]

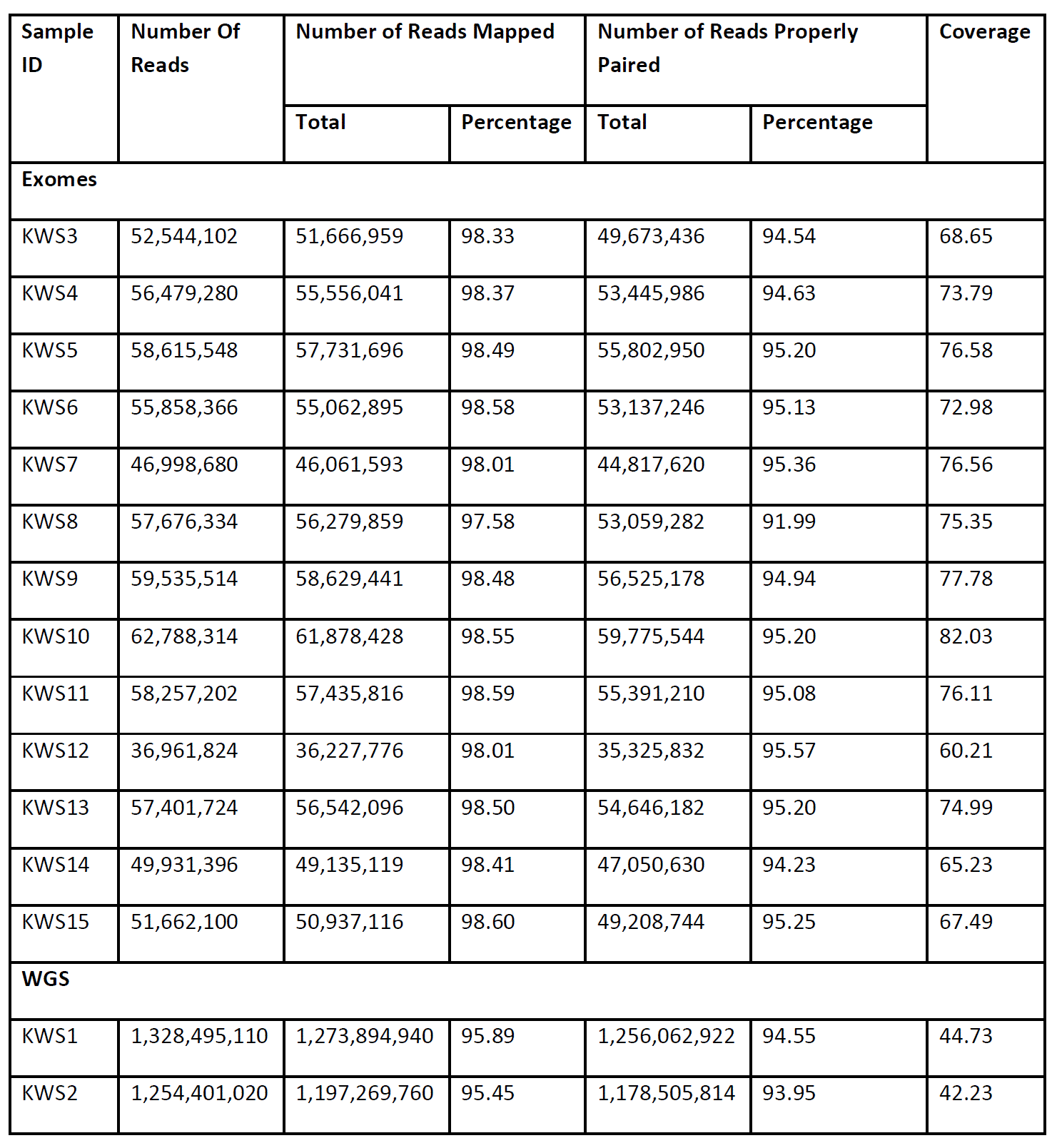

Supplement: Table S6 — Alignment statistics and genome coverage for the KWS1 and KWS2 (whole genome) samples. For calculating the percentage of genome covered, the length of human genome is considered as 3,000,000,000 bps (for WGS) and 62,000,000 (for exomes). (GIF) [file pone.0099069.s010.gif]
