# Supplementary material for: Genome at Juncture of Early Human Migration: A Systematic Analysis of Two Whole Genomes and Thirteen Exomes from Kuwaiti Population Subgroup of Inferred Saudi Arabian Tribe Ancestry
Source: PLoS One. 2014 Jun 4;9(6):e99069. doi: 10.1371/journal.pone.0099069 (PMC4045902; doi:10.1371/journal.pone.0099069)
Supplement: Table S1 — Phenotype details on the 15 the samples sequenced. (DOCX) [file pone.0099069.s005.docx]

| Sample ID | Ancestry composition (Arab; West Asian, European; African) |
| --- | --- |
| **Whole Genome Sequencing** | |
| KWS1: Male | 77.0%; 9.5%; 11.6%; 0.9% |
| KWS2: Male | 71.0%; 13.5%; 12%; 3.1% |
| **Exome Sequencing** | |
| KWS3: Female | 67.6%; 14.1%; 12.3%; 5.1% |
| KWS4: Female | 73.1%; 8.2%; 12%; 3.1% |
| KWS5: Male | 72.2%; 11%; 12.8%; 3.6% |
| KWS6: Female | 83.5%; 7.6%; 8.1%; 0.3% |
| KWS7: Male | 71.4%; 11.6%; 13%; 1.7% |
| KWS8: Male | 67.3%; 8.2%; 10.8%; 0.1% |
| KWS9: Male | 81%; 9.3%; 7.5%; 1.3% |
| KWS10: Male | 80.9%; 9.1%; 6.3%; 2.0% |
| KWS11: Female | 74.3%; 12.3%; 11.5%; 0.9% |
| KWS12: Female | 73.2%; 11.8%; 9.5%; 3.7% |
| KWS13: Male | 86.5%; 6.2%; 5.8%; 0.1% |
| KWS14: Male | 70.4%; 15.8%; 11.6%; 1.9% |
| KWS15: Female | 66.1%; 18.5%; 13.4%; 1.6% |

For the sake of confidentiality with respect to the identity of the participants, the names of the tribes to which they participants belong to are not presented in the table.
