# Supplementary material for: Genome at Juncture of Early Human Migration: A Systematic Analysis of Two Whole Genomes and Thirteen Exomes from Kuwaiti Population Subgroup of Inferred Saudi Arabian Tribe Ancestry
Source: PLoS One. 2014 Jun 4;9(6):e99069. doi: 10.1371/journal.pone.0099069 (PMC4045902; doi:10.1371/journal.pone.0099069)
Supplement: Table S2 — Classification of triallelic SNP variants from the UE and UW data set. (PDF) [file pone.0099069.s006.pdf]

| Class$ | UE dataset | | | | UW dataset | | | |
|---|---|---|---|---|---|---|---|---|
| | SNP | | Indel | | SNP | | Indel | |
| | Known | Novel | Known | Novel | Known | Novel | Known | Novel |
| Coding | 38 | 7 | 0 | 4 | 0 | 0 | 0 | 0 |
| Coding, Splicing | 0 | 0 | 0 | 0 | 0 | 0 | 0 | 0 |
| Downstream | 0 | 0 | 0 | 0 | 5 | 0 | 0 | 28 |
| Downstream, Upstream | 0 | 0 | 0 | 0 | 0 | 0 | 0 | 0 |
| Intergenic | 1 | 0 | 0 | 0 | 531 | 188 | 0 | 2107 |
| Intronic | 0 | 0 | 0 | 4 | 262 | 72 | 0 | 1272 |
| NCExonic | 11 | 3 | 0 | 14 | 1 | 0 | 0 | 1 |
| NCSplicing | 0 | 0 | 0 | 0 | 0 | 0 | 0 | 0 |
| Splicing | 0 | 0 | 0 | 0 | 0 | 0 | 0 | 0 |
| 3’ UTR | 27 | 13 | 0 | 266 | 0 | 0 | 0 | 2 |
| 3’ UTR, 5’ UTR | 0 | 0 | 0 | 0 | 0 | 0 | 0 | 0 |
| 5’ UTR | 8 | 1 | 0 | 18 | 0 | 0 | 0 | 1 |
| Upstream | 0 | 0 | 0 | 0 | 5 | 1 | 0 | 17 |

**$, Legends to the class types**

**Coding -** Variant is in the coding exonic region of a protein coding transcript.
**Splicing -** Variant affects a nucleotide that is in a splicing region of a coding transcript.

**Downstream -** Variant is within 1000 bp of the transcript stop site on the 3’ side.

**Upstream -** Variant is within 1000 bp of the transcript start site on the 5’ side.

**Intergenic -** Variant does not interact with any gene transcripts.
**Intronic -** Variant lies within an intron.

**NCSplicing -** Variant affects a nucleotide that is in a splicing region of a non-coding transcript.

**NCExonic -** Variant is in an exon for a non-coding transcript.
**UTR5 -** Variant is in an exon of a coding transcript but is on the 5’ side of the start codon.
**UTR3 -** Variant is in an exon of a coding transcript but is on the 3’ side of the stop codon.
