# Supplementary material for: Genome at Juncture of Early Human Migration: A Systematic Analysis of Two Whole Genomes and Thirteen Exomes from Kuwaiti Population Subgroup of Inferred Saudi Arabian Tribe Ancestry
Source: PLoS One. 2014 Jun 4;9(6):e99069. doi: 10.1371/journal.pone.0099069 (PMC4045902; doi:10.1371/journal.pone.0099069)
Supplement: Table S3 — List of SNPs associated (GWAS-linked) with diseases and risk factors. (PDF) [file pone.0099069.s007.pdf]

**UE dataset**

| SNPs | Human Condition |
|---|---|
| rs3748816 | Celiac disease |
| rs846111 | QT interval |
| rs1801133 | Homocysteine levels |
| rs2240335 | Rheumatoid arthritis |
| rs9253 | Hemostatic factors and hematological phenotypes |
| rs11209026 | Ankylosing spondylitis,Crohn's disease,Ulcerative colitis,Psoriasis,Inflammatory bowel disease |
| rs10889677 | Crohn's disease,Ulcerative colitis |
| rs12740374 | LDL cholesterol,Coronary heart disease,Lipoprotein-associated phospholipase A2 activity and mass |
| rs660240 | Cardiovascular disease risk factors,LDL cholesterol |
| rs629301 | Cholesterol, total,LDL cholesterol |
| rs2476601 | DIABETES MELLITUS INSULIN-DEPENDENT,DIABETES MELLITUS INSULIN-DEPENDENT SUSCEPTIBILITY TO,HYPOADRENOCORTICISM FAMILIAL,PROTEIN TYROSINE PHOSPHATASE NONRECEPTOR-TYPE 22,SYSTEMIC LUPUS ERYTHEMATOSUS,Myasthenia gravis ,Crohn's disease,Crohn's disease,RHEUMATOID ARTHRITIS,Systemic Lupus Erythematosus (SLE) gender differentiated in women,Type I Diabetes,BODY MASS INDEX,Height,Two-hour glucose (BMI adjusted),Fasting plasma glucose,Fasting insulin,Fasting proinsulin,Beta-cell function (HOMA-B),Insulin resistance (HOMA-IR),HbA1c,RHEUMATOID ARTHRITIS,RHEUMATOID ARTHRITIS,RHEUMATOID ARTHRITIS,Type 1 diabetes,Type 1 diabetes,Type 1 diabetes,Type 1 diabetes autoantibodies,Vitiligo,Ulcerative colitis,Crohns Disease,DIABETES MELLITUS INSULIN-DEPENDENT SUSCEPTIBILITY TO,RHEUMATOID ARTHRITIS,SYSTEMIC LUPUS ERYTHEMATOSUS SUSCEPTIBILITY TO,HASHIMOTO THYROIDITIS SUSCEPTIBILITY TO,Addison_disease_susceptibility_to,Advanced age related macular degeneration,Total Cholesterol,Triglycerides,High Density Lipoprotein Cholesterol,Low-density lipoprotein cholesterol |
| rs4074536 | Ventricular conduction |
| rs11803731 | Hair morphology |
| rs4072037 | Magnesium levels,Esophageal cancer and gastric cancer |
| rs2282301 | Conduct disorder (interaction) |
| rs6684514 | mean corpuscular hemoglobin concentration |
| rs1801274 | Kawasaki disease,Ulcerative colitis,Inflammatory bowel disease |
| rs6136 | Soluble levels of adhesion molecules |
| rs2274432 | Height |
| rs1061170 | Age-related macular degeneration,Age-related macular degeneration (CNV),Age-related macular degeneration (GA) |
| rs4950928 | YKL-40 levels |
| rs6683071 | Cognitive performance |
| rs693 | Triglycerides,Cholesterol, total,LDL cholesterol |

| | |
|---|---|
| rs1260326 | Urate levels,Cardiovascular disease risk factors,Serum albumin level,Chronic kidney disease,Cholesterol, total,Two-hour glucose challenge,Lipid metabolism phenotypes,Hypertriglyceridemia,Platelet counts,Metabolite levels,Non-albumin protein levels,Waist circumference and related phenotypes,Metabolic traits,C-reactive protein,Serum total protein level,Triglycerides,Liver enzyme levels (gamma-glutamyl transferase),Hematological and biochemical traits,Lipoprotein-associated phospholipase A2 activity and mass |
| rs3099950 | Amyotrophic lateral sclerosis |
| rs7578597 | Type 2 diabetes |
| rs6756629 | Cholesterol, total,LDL cholesterol |
| rs11887534 | Gallstones |
| rs13538 | Chronic kidney disease |
| rs6761276 | Protein quantitative trait loci |
| rs17727261 | Response to antipsychotic treatment |
| rs1799810 | Self-rated health |
| rs1990760 | Immunoglobulin A,Type 1 diabetes,Type 1 diabetes autoantibodies |
| rs4668356 | Cognitive performance |
| rs11888559 | Height |
| rs2286963 | Metabolite levels |
| rs2571445 | Pulmonary function,Pulmonary function (interaction) |
| rs12621643 | Acute lymphoblastic leukemia (childhood) |
| rs2241880 | Crohn's disease |
| rs3792109 | Crohn's disease |
| rs757978 | Chronic lymphocytic leukemia |
| rs3749380 | Panic disorder |
| rs9036 | Cognitive performance |
| rs4973768 | Breast cancer |
| rs6795970 | Electrocardiographic conduction measures,Electrocardiographic traits |
| rs864643 | Attention deficit hyperactivity disorder |
| rs2286720 | Reasoning |
| rs9858542 | Crohn's disease,Ulcerative colitis |
| rs3197999 | Crohn's disease,Ulcerative colitis,Primary sclerosing cholangitis,Inflammatory bowel disease |
| rs2251219 | Major mood disorders |
| rs1042779 | Bipolar disorder |
| rs9835332 | Height |
| rs1801725 | Calcium levels |
| rs1799852 | Iron status biomarkers |
| rs9818870 | Coronary heart disease |
| rs10936599 | Celiac disease,Colorectal cancer,Multiple sclerosis,Telomere length |
| rs572169 | Height |
| rs3914188 | Menarche (age at onset) |
| rs9898 | Activated partial thromboplastin time |
| rs710446 | Activated partial thromboplastin time |
| rs6773957 | Adiponectin levels |
| rs1801214 | Type 2 diabetes |

| | |
|---|---|
| rs16890979 | Urate levels,Uric acid levels |
| rs1379659 | Echocardiographic traits |
| rs2231142 | Urate levels,Uric acid levels |
| rs1229984 | Esophageal cancer,Upper aerodigestive tract cancers,Alcohol dependence |
| rs10516487 | Systemic lupus erythematosus |
| rs2285714 | Age-related macular degeneration |
| rs6056 | Fibrinogen |
| rs8396 | Metabolite levels,Metabolic traits |
| rs16891982 | Hair color,Eye color,Skin pigmentation |
| rs6897932 | Type 1 diabetes,Multiple sclerosis |
| rs3194051 | Ulcerative colitis |
| rs1445898 | Type 1 diabetes |
| rs12916 | Cholesterol, total,LDL cholesterol |
| rs13160562 | Alcohol dependence |
| rs27434 | Ankylosing spondylitis |
| rs10089 | Ileal carcinoids |
| rs20541 | Hodgkin's lymphoma,Psoriasis,IgE levels |
| rs365132 | Menarche and menopause (age at onset),Menopause (age at onset) |
| rs872071 | Chronic lymphocytic leukemia |
| rs16889440 | Radiation response |
| rs1165196 | Urate levels |
| rs1799945 | Diastolic blood pressure,Iron levels,Hypertension,Systolic blood pressure |
| rs145004927 | Hematological parameters |
| rs1405069 | Chemerin levels |
| rs1805017 | Lipoprotein-associated phospholipase A2 activity and mass |
| rs783396 | Stroke |
| rs458017 | Psoriasis |
| rs6901250 | C-reactive protein |
| rs9390459 | Coagulation factor levels |
| rs6929137 | Bone mineral density (spine) |
| rs3734805 | Breast cancer |
| rs1738074 | Celiac disease,Multiple sclerosis |
| rs4132601 | Acute lymphoblastic leukemia (childhood) |
| rs875971 | Aortic root size |
| rs3812316 | Triglycerides |
| rs12666989 | Resting heart rate |
| rs713598 | Bitter taste response |
| rs328 | HDL cholesterol,Triglycerides |
| rs9331888 | Alzheimer's disease |
| rs13266634 | Type 2 diabetes,Glycated hemoglobin levels,Type 2 diabetes and other traits |
| rs3802177 | Type 2 diabetes |
| rs11558471 | Proinsulin levels,Fasting glucose-related traits (interaction with BMI),Fasting glucose-related traits |
| rs2294015 | Event-related brain oscillations |

| | |
|---|---|
| rs1036819 | BODY MASS INDEX,Height,Two-hour glucose (BMI adjusted),Fasting plasma glucose,Fasting insulin,Fasting proinsulin,Beta-cell function (HOMA-B),Insulin resistance (HOMA-IR),HbA1c,Ulcerative colitis,Crohns Disease,Advanced age related macular degeneration,Total Cholesterol,Triglycerides,High Density Lipoprotein Cholesterol,Low-density lipoprotein cholesterol |
| rs2294008 | Duodenal ulcer,Bladder cancer |
| rs2290416 | Attention deficit hyperactivity disorder |
| rs17641078 | Hyperactive-impulsive symptoms |
| rs1063192 | Glaucoma (primary open-angle),Vertical cup-disc ratio |
| rs564398 | Type 2 diabetes |
| rs10122902 | Amyotrophic lateral sclerosis |
| rs774359 | Amyotrophic lateral sclerosis |
| rs3810936 | Crohn's disease |
| rs8176746 | mean corpuscular hemoglobin concentration |
| rs10781499 | Ulcerative colitis,Inflammatory bowel disease |
| rs4077515 | Crohn's disease,Ulcerative colitis |
| rs3739998 | Coronary heart disease |
| rs1148259 | Metabolite levels |
| rs7097397 | Systemic lupus erythematosus |
| rs2244967 | Uric acid levels |
| rs7916697 | Optic disc parameters |
| rs2234978 | Immunoglobulin A |
| rs3765524 | Dengue shock syndrome,Esophageal cancer and gastric cancer |
| rs1799853 | Warfarin maintenance dose |
| rs1057910 | Warfarin maintenance dose |
| rs4387287 | Telomere length |
| rs10490924 | Age-related macular degeneration,Age-related macular degeneration (GA),Age-related macular degeneration (CNV),Age-related macular degeneration (CNV vs. GA) |
| rs3741208 | Type 1 diabetes |
| rs231362 | Type 2 diabetes |
| rs4929923 | Menarche (age at onset) |
| rs5215 | Type 2 diabetes |
| rs193929332 | Type 2 diabetes |
| rs174546 | Metabolic syndrome,Cholesterol, total,HDL cholesterol,Triglycerides,LDL cholesterol |
| rs6591182 | Non-alcoholic fatty liver disease histology (lobular) |
| rs3736228 | Bone mineral density |
| rs3750965 | Hair color |
| rs35264875 | Blond vs. brown hair color |
| rs1552224 | Type 2 diabetes |
| rs1042602 | Freckles,Skin pigmentation |
| rs28927680 | Triglycerides |
| rs498872 | Glioma |
| rs2126709 | Response to antipsychotic therapy (extrapyramidal side effects) |
| rs887304 | Non-alcoholic fatty liver disease histology (lobular) |
| rs3764021 | Type 1 diabetes |

| | |
|---|---|
| rs4149056 | Sex hormone-binding globulin levels,Bilirubin levels,Response to statin therapy |
| rs703842 | Multiple sclerosis |
| rs1042725 | Head circumference (infant),Birth weight,Height |
| rs8756 | Height |
| rs995030 | Testicular germ cell tumor |
| rs2292354 | Metabolic syndrome |
| rs3184504 | Beta-2 microglubulin plasma levels,Diastolic blood pressure,Eosinophil counts,Rheumatoid arthritis,Red blood cell traits,Coronary heart disease,Type 1 diabetes,Platelet counts,Systolic blood pressure,Type 1 diabetes autoantibodies,Hypothyroidism |
| rs883079 | Ventricular conduction |
| rs1169310 | C-reactive protein |
| rs4770433 | Protein quantitative trait loci |
| rs3764147 | Crohn's disease,Leprosy |
| rs2031532 | Cardiac hypertrophy |
| rs3742207 | Arterial stiffness |
| rs365990 | Electrocardiographic traits,Heart rate |
| rs8192917 | Vitiligo |
| rs7157940 | Anthropometric traits |
| rs2229116 | Carotid atherosclerosis in HIV infection |
| rs3743266 | Menarche (age at onset) |
| rs3825942 | Glaucoma (exfoliation) |
| rs5742915 | Height,Paget's disease |
| rs13180 | Chronic obstructive pulmonary disease |
| rs1051730 | Lung adenocarcinoma,Nicotine dependence,Smoking behavior,Lung cancer |
| rs4842838 | Height |
| rs8040009 | Bipolar disorder,Alcoholism (heaviness of drinking) |
| rs1065656 | Insulin-like growth factors |
| rs1136001 | Height |
| rs7498665 | Body mass index,Weight,Obesity |
| rs2217332 | Metabolic syndrome |
| rs12449157 | HDL cholesterol |
| rs17689437 | HIV-1 viral setpoint,Reasoning |
| rs4785763 | Melanoma |
| rs12150338 | Calcium levels |
| rs6065 | Platelet counts |
| rs6761 | Protein quantitative trait loci |
| rs907092 | Primary biliary cirrhosis |
| rs2305480 | Ulcerative colitis,Asthma |
| rs3894194 | Asthma |
| rs227584 | Bone mineral density (hip),Bone mineral density |
| rs199533 | Parkinson's disease |
| rs2257205 | Pancreatic cancer |
| rs4343 | Angiotensin-converting enzyme activity |
| rs11077773 | Information processing speed |
| rs3744064 | Cognitive performance |

| | |
|---|---|
| rs9894429 | Eye color traits |
| rs1046896 | Glycated hemoglobin levels |
| rs1805081 | Obesity |
| rs763361 | Type 1 diabetes,Type 1 diabetes autoantibodies |
| rs7250872 | Bipolar disorder |
| rs2230199 | Age-related macular degeneration,Age-related macular degeneration (GA),Age-related macular degeneration (CNV) |
| rs1799969 | Soluble ICAM-1 |
| rs5498 | Soluble ICAM-1 |
| rs281437 | Soluble ICAM-1 |
| rs2304256 | Type 1 diabetes,Type 1 diabetes autoantibodies |
| rs2228671 | Cholesterol, total,LDL cholesterol |
| rs3745672 | Multiple sclerosis |
| rs17638629 | Cognitive performance |
| rs2108622 | Vitamin E levels,Warfarin maintenance dose,Response to Vitamin E supplementation,Metabolite levels,Acenocoumarol maintenance dosage |
| rs8170 | Breast Cancer in BRCA1 mutation carriers,Ovarian cancer,Breast cancer |
| rs2363956 | Ovarian cancer |
| rs892055 | Asperger disorder |
| rs1434579 | Tuberculosis |
| rs6859 | Alzheimer's disease (late onset),Alzheimer's disease |
| rs429358 | Alzheimer's disease biomarkers,Brain imaging |
| rs3810291 | Body mass index |
| rs492602 | Cholesterol, total,Vitamin B12 levels |
| rs602662 | Folate pathway vitamin levels |
| rs504963 | Crohn's disease |
| rs17632542 | Prostate-specific antigen levels |
| rs1671152 | Platelet aggregation |
| rs1126757 | Response to antidepressants |
| rs2014572 | Hyperactive-impulsive symptoms |
| rs1799990 | Creutzfeldt-Jakob disease,Prion diseases |
| rs16991615 | Menarche and menopause (age at onset),Menopause (age at onset) |
| rs867186 | Hemostatic factors and hematological phenotypes,Anticoagulant levels,Protein C levels,D-dimer levels,Coagulation factor levels |
| rs8115854 | Hippocampal atrophy |
| rs7679 | HDL cholesterol,Triglycerides |
| rs495337 | Psoriasis |
| rs2023454 | Functional MRI |
| rs2073145 | Hippocampal atrophy |
| rs2229741 | Cognitive performance |
| rs219780 | Kidney stones |
| rs4819388 | Celiac disease |
| rs2298428 | Celiac disease,Celiac disease and Rheumatoid arthritis |
| rs4823006 | Waist-hip ratio |
| rs2239785 | Glomerulosclerosis |

| | |
|---|---|
| rs855791 | Mean corpuscular hemoglobin concentration,Mean corpuscular hemoglobin,Mean corpuscular volume,mean corpuscular hemoglobin concentration,Red blood cell traits,Iron status biomarkers,Hemoglobin,Hematology traits,Glycated hemoglobin levels,Hepcidin levels |
| rs4820268 | Red blood cell traits,Iron status biomarkers,Iron levels,Hematology traits |
| rs229527 | Vitiligo,Graves' disease |
| rs5771069 | Ulcerative colitis |
| **UW Dataset** | |
| rs3934834 | Body mass index |
| rs3753242 | Reasoning |
| rs734999 | Ulcerative colitis |
| rs966321 | Factor VII |
| rs12025126 | Vertical cup-disc ratio |
| rs10492972 | Multiple sclerosis |
| rs17401966 | Hepatocellular carcinoma |
| rs12741973 | Optic disc size (rim) |
| rs12046278 | Systolic blood pressure |
| rs4846033 | Schizophrenia |
| rs17367504 | Systolic blood pressure,Blood pressure |
| rs2901964 | Erectile dysfunction and prostate cancer treatment |
| rs9442235 | Cognitive performance |
| rs2284746 | Pulmonary function,Pulmonary function (interaction),Height |
| rs3738814 | Height |
| rs12063142 | Parkinson's disease |
| rs1317209 | Ulcerative colitis |
| rs3806308 | Ulcerative colitis |
| rs6426833 | Ulcerative colitis |
| rs4654925 | Ulcerative colitis |
| rs4654748 | Folate pathway vitamin levels |
| rs1780324 | Liver enzyme levels |
| rs1697421 | Phosphorus levels |
| rs7524102 | Bone mineral density (hip),Bone mineral density (spine),Bone mineral density,Ulcerative colitis,Dupuytren's disease |
| rs4649203 | Psoriasis |
| rs10903122 | Celiac disease |
| rs10903129 | Erythrocyte sedimentation rate,Cholesterol, total |
| rs11809207 | Height |
| rs4949526 | Bipolar disorder and schizophrenia |
| rs910696 | Smoking behavior |
| rs2180233 | Attention deficit hyperactivity disorder and conduct disorder |
| rs476463 | Brain structure |
| rs2281597 | Attention deficit hyperactivity disorder |
| rs12131057 | Rheumatoid arthritis |
| rs12037222 | C-reactive protein |
| rs873917 | Amyotrophic lateral sclerosis |
| rs6686842 | Height |

| | |
|---|---|
| rs6588480 | Response to statin therapy |
| rs11206510 | Myocardial infarction (early onset),LDL cholesterol,Coronary heart disease |
| rs11206801 | AB1-42 |
| rs527409 | Kawasaki disease |
| rs2811893 | Diabetic retinopathy |
| rs472913 | Bipolar disorder |
| rs6691768 | Celiac disease |
| rs9436640 | Ventricular conduction |
| rs1167998 | Triglycerides,Lipid metabolism phenotypes |
| rs1168013 | Triglycerides |
| rs1748195 | Triglycerides |
| rs10889353 | Triglycerides,Cholesterol, total,LDL cholesterol,Lipid metabolism phenotypes |
| rs10493340 | Blood pressure |
| rs1751492 | Soluble leptin receptor levels |
| rs4420065 | C-reactive protein |
| rs12753193 | Metabolic traits |
| rs11209002 | Crohn's disease |
| rs2064689 | Crohn's disease |
| rs1004819 | Crohn's disease |
| rs11805303 | Crohn's disease |
| rs11465802 | Crohn's disease |
| rs2201841 | Crohn's disease,Ulcerative colitis,Psoriasis |
| rs11465804 | Crohn's disease |
| rs1343151 | Crohn's disease |
| rs10889676 | Crohn's disease |
| rs9988642 | Crohn's disease |
| rs12567232 | Crohn's disease |
| rs6669582 | Crohn's disease |
| rs10789230 | Crohn's disease |
| rs1495965 | Behcet's disease |
| rs924080 | Behcet's disease |
| rs3790567 | Primary biliary cirrhosis |
| rs4147141 | Inattentive symptoms,Attention deficit hyperactivity disorder |
| rs10493485 | Hemostatic factors and hematological phenotypes |
| rs2568958 | Body mass index,Obesity,Weight |
| rs2815752 | Body mass index |
| rs11210359 | Bipolar disorder and schizophrenia |
| rs1514175 | Body mass index |
| rs211718 | Metabolite levels,Metabolic traits |
| rs12753569 | Personality dimensions |
| rs10873876 | Acute lymphoblastic leukemia (childhood) |
| rs7533906 | Bipolar disorder and schizophrenia |
| rs12024204 | Endometriosis |
| rs11163585 | Response to antipsychotic treatment |
| rs7552393 | Select biomarker traits |
| rs7539409 | Alzheimer's disease |
| rs1085093 | Metabolic syndrome |

| | |
|---|---|
| rs1983853 | Type 1 diabetes |
| rs7553864 | Smoking behavior |
| rs983332 | Response to TNF antagonist treatment |
| rs2136093 | Response to antidepressants |
| rs164898 | Metabolic syndrome |
| rs1192415 | Optic disc parameters,Optic disc size (disc) |
| rs6604026 | Multiple sclerosis |
| rs12745968 | Bipolar disorder and schizophrenia |
| rs1414896 | Non-alcoholic fatty liver disease histology (AST) |
| rs6693882 | Pain |
| rs1973993 | Weight |
| rs10783050 | Body mass index |
| rs7544736 | Schizophrenia |
| rs7543130 | Aortic root size |
| rs10874639 | Protein quantitative trait loci |
| rs12726652 | Working memory |
| rs4118325 | AIDS |
| rs646776 | Cholesterol, total,Myocardial infarction (early onset),LDL cholesterol,Progranulin levels,Lipid metabolism phenotypes,Response to statin therapy,Coronary heart disease |
| rs599839 | Metabolite levels,LDL cholesterol,Lipoprotein-associated phospholipase A2 activity and mass,Coronary heart disease |
| rs1933182 | Chronic kidney disease |
| rs484959 | Paget's disease |
| rs958798 | Self-rated health |
| rs12061304 | Panic disorder |
| rs494453 | Osteoporosis |
| rs7555668 | Cognitive performance |
| rs17038182 | Height |
| rs12735613 | Height |
| rs984222 | Waist-hip ratio |
| rs11249433 | Breast cancer |
| rs12129861 | Uric acid levels |
| rs12122100 | HIV-1 control |
| rs4950322 | Protein quantitative trait loci |
| rs11205277 | Height |
| rs267734 | Chronic kidney disease |
| rs10788819 | Hair morphology |
| rs3124314 | Hair morphology |
| rs17646946 | Common traits (Other) |
| rs3120665 | Personality dimensions |
| rs908922 | Hair morphology |
| rs499697 | Common traits (Other) |
| rs10888501 | Response to antipsychotic treatment |
| rs4085613 | Psoriasis |
| rs4112788 | Psoriasis |
| rs4845552 | Hippocampal atrophy |

| | |
|---|---|
| rs4434872 | Conduct disorder (symptom count) |
| rs4537545 | C-reactive protein |
| rs4129267 | Asthma,Protein quantitative trait loci,Pulmonary function,C-reactive protein |
| rs13376333 | Atrial fibrillation |
| rs6427356 | Attention deficit hyperactivity disorder and conduct disorder |
| rs2779116 | Glycated hemoglobin levels |
| rs857721 | Other erythrocyte phenotypes |
| rs1474747 | Select biomarker traits |
| rs10489849 | Select biomarker traits |
| rs2494250 | Select biomarker traits |
| rs6687840 | Chemerin levels |
| rs4446959 | Chemerin levels |
| rs12093699 | Protein quantitative trait loci |
| rs2794520 | C-reactive protein,Select biomarker traits,Metabolic traits |
| rs2808630 | Lung cancer |
| rs3091244 | C-reactive protein |
| rs3093059 | C-reactive protein |
| rs7553007 | C-reactive protein |
| rs2274910 | Crohn's disease |
| rs10800309 | Ulcerative colitis |
| rs10918270 | Parkinson's disease (age of onset) |
| rs2880058 | QT interval |
| rs12143842 | QT interval |
| rs1415259 | Electrocardiographic traits |
| rs10494366 | Electrocardiographic traits,QT interval |
| rs6670655 | Height |
| rs1532815 | Response to acetaminophen (hepatotoxicity) |
| rs466639 | Menarche (age at onset) |
| rs4657482 | Testicular germ cell tumor |
| rs9803659 | Liver enzyme levels |
| rs840016 | Rheumatoid arthritis |
| rs864537 | Celiac disease,Celiac disease and Rheumatoid arthritis |
| rs2056626 | Systemic sclerosis |
| rs1412337 | Morbidity-free survival |
| rs10919071 | QT interval |
| rs2235302 | Soluble levels of adhesion molecules |
| rs1569476 | Total ventricular volume |
| rs1541160 | Amyotrophic lateral sclerosis |
| rs10914144 | Platelet counts,Mean platelet volume |
| rs678962 | Height |
| rs1011731 | Waist-hip ratio |
| rs75699797 | Celiac disease |
| rs9286879 | Crohn's disease |
| rs12035082 | Crohn's disease |
| rs2205960 | Systemic lupus erythematosus |
| rs10798269 | Systemic lupus erythematosus |
| rs3766680 | Tonometry |

| | |
|---|---|
| rs633715 | Menarche (age at onset),Body mass index,Obesity |
| rs543874 | Body mass index |
| rs10913469 | Body mass index,Weight |
| rs4651156 | Response to antidepressants |
| rs10797919 | Attention deficit hyperactivity disorder and conduct disorder |
| rs4140564 | Knee osteoarthritis |
| rs1400544 | Subclinical atherosclerosis traits (other) |
| rs2053302 | Response to statin therapy |
| rs10801047 | Crohn's disease |
| rs2816316 | Celiac disease |
| rs606149 | Major depressive disorder |
| rs1890645 | Neonatal lupus |
| rs1329424 | Age-related macular degeneration |
| rs10737680 | Age-related macular degeneration |
| rs1410996 | Age-related macular degeneration,End-stage coagulation |
| rs380390 | Age-related macular degeneration |
| rs1329428 | Age-related macular degeneration |
| rs426736 | Meningococcal disease |
| rs6428370 | Acute lymphoblastic leukemia (childhood) |
| rs2786098 | Asthma |
| rs1998598 | Crohn's disease |
| rs12127588 | Mean corpuscular hemoglobin |
| rs7512898 | Electrocardiographic conduction measures |
| rs7554511 | Crohn's disease,Ulcerative colitis,Inflammatory bowel disease |
| rs296547 | Celiac disease |
| rs11584383 | Crohn's disease,Ulcerative colitis |
| rs860554 | Panic disorder |
| rs12143943 | Cognitive performance |
| rs823128 | Parkinson's disease |
| rs947211 | Parkinson's disease |
| rs11240594 | Response to antipsychotic treatment |
| rs1518111 | Behcet's disease |
| rs1800871 | Behcet's disease |
| rs3813948 | C4b binding protein levels |
| rs6656401 | Alzheimer's disease |
| rs3818361 | Alzheimer's disease |
| rs4844614 | LDL cholesterol |
| rs2745967 | Resting heart rate |
| rs642961 | Orofacial clefts |
| rs340874 | Fasting glucose-related traits,Fasting glucose-related traits (interaction with BMI) |
| rs12757165 | Cardiac hypertrophy |
| rs1436900 | Optic disc size (cup) |
| rs2605100 | Adiposity |
| rs4846567 | Waist-hip ratio |
| rs337161 | Response to antipsychotic therapy (extrapyramidal side effects) |
| rs1494373 | Response to antipsychotic therapy (extrapyramidal side effects) |

| | |
|---|---|
| rs6691170 | Colorectal cancer |
| rs6687758 | Colorectal cancer,Progressive supranuclear palsy |
| rs873549 | Keloid |
| rs17465637 | Myocardial infarction (early onset),Coronary heart disease |
| rs11579964 | Major depressive disorder |
| rs1390401 | Height |
| rs801114 | Basal cell carcinoma |
| rs2144300 | HDL cholesterol,Triglycerides,Circulating myeloperoxidase levels (serum) |
| rs4846914 | HDL cholesterol,Triglycerides |
| rs10489615 | HDL cholesterol |
| rs701157 | Attention deficit hyperactivity disorder and conduct disorder |
| rs10746514 | Response to statin therapy |
| rs669408 | Immunoglobulin A |
| rs2069084 | HIV-1 control |
| rs6429082 | Adiposity |
| rs291353 | Aging traits |
| rs7554607 | Acute lymphoblastic leukemia (childhood) |
| rs2819770 | Exercise treadmill test traits |
| rs2499604 | Non-alcoholic fatty liver disease histology (AST) |
| rs2820037 | Hypertension |
| rs12569163 | Radiation response |
| rs1539019 | Fibrinogen |
| rs12239046 | C-reactive protein |
| rs1881797 | Acute lymphoblastic leukemia (childhood) |
| rs11204538 | Mean corpuscular volume |
| rs4481887 | Common traits (Other) |
| rs4643574 | Cognitive performance |
| rs2947411 | Menarche (age at onset) |
| rs2867125 | Body mass index |
| rs6548238 | Body mass index |
| rs7561317 | Body mass index,Weight |
| rs6735179 | Response to antipsychotic treatment |
| rs1377638 | Low-density lipoprotein cholesterol |
| rs1405262 | HIV-1 control |
| rs2357266 | Self-rated health |
| rs6739054 | Cognitive performance |
| rs10929808 | Bipolar disorder and schizophrenia |
| rs1534422 | Type 1 diabetes |
| rs7581919 | Conduct disorder (case status) |
| rs7586898 | Hair morphology |
| rs7580332 | Amyotrophic lateral sclerosis |
| rs13385191 | Prostate cancer |
| rs11902417 | HDL cholesterol |
| rs6544366 | Triglycerides |
| rs6754295 | HDL cholesterol,Triglycerides,Lipid metabolism phenotypes |
| rs7557067 | Triglycerides |

| | |
|---|---|
| rs673548 | Metabolic syndrome,Metabolite levels,Metabolic traits,Lipid metabolism phenotypes |
| rs515135 | LDL cholesterol |
| rs562338 | LDL cholesterol |
| rs541041 | Response to statin therapy |
| rs219553 | Erectile dysfunction and prostate cancer treatment |
| rs1117324 | Response to antipsychotic treatment |
| rs7561273 | Quantitative traits |
| rs6707600 | Working memory |
| rs2165738 | Type 1 diabetes |
| rs713586 | Body mass index |
| rs4665736 | Height |
| rs6733301 | Height |
| rs13428812 | Crohn's disease |
| rs780094 | Uric acid levels,Metabolic syndrome,Urate levels,Metabolic traits,Fasting insulin-related traits (interaction with BMI),Fasting glucose-related traits (interaction with BMI),Phospholipid levels (plasma),C-reactive protein,Triglycerides,LDL cholesterol,Fasting glucose-related traits,Fasting insulin-related traits |
| rs780093 | Triglycerides-Blood Pressure (TG-BP),Crohn's disease,Waist Circumference - Triglycerides (WC-TG),Sex hormone-binding globulin levels,Urate levels,Palmitoleic acid (16:1n-7) plasma levels,Calcium levels,Palmitic acid (16:0) plasma levels,Breast size |
| rs1260333 | Triglycerides |
| rs882632 | Major depressive disorder |
| rs647316 | Mean platelet volume |
| rs41464348 | Height |
| rs13385731 | Systemic lupus erythematosus |
| rs9308945 | Hypertension (young onset) |
| rs6711736 | Hypertension (young onset) |
| rs10495809 | Hypertension (young onset) |
| rs6733379 | Attention deficit hyperactivity disorder and conduct disorder |
| rs7562790 | Ventricular conduction |
| rs17020136 | Ventricular conduction |
| rs4352210 | RR interval (heart rate) |
| rs17511102 | Height |
| rs604381 | Conduct disorder (interaction) |
| rs4670779 | Bone mineral density (spine) |
| rs6741148 | Erectile dysfunction and prostate cancer treatment |
| rs2540226 | Personality dimensions |
| rs719593 | Conduct disorder (interaction) |
| rs930421 | Attention deficit hyperactivity disorder |
| rs6719977 | Hyperactive-impulsive symptoms |
| rs7590268 | Orofacial clefts |
| rs1465618 | Prostate cancer |
| rs6732426 | Hair morphology |
| rs12478601 | Polycystic ovary syndrome |

| | |
|---|---|
| rs6544713 | LDL cholesterol |
| rs4245791 | Phytosterol levels,LDL cholesterol |
| rs10495928 | Hemoglobin,Red blood cell count,Hematological and biochemical traits |
| rs10168349 | Hematocrit,Hematological and biochemical traits |
| rs7579899 | Renal cell carcinoma |
| rs6544997 | Vitiligo |
| rs7591064 | Radiation response |
| rs2268363 | Erectile dysfunction and prostate cancer treatment |
| rs1160297 | Hemostatic factors and hematological phenotypes |
| rs2357013 | Hemostatic factors and hematological phenotypes |
| rs11898505 | Bone mineral density (spine) |
| rs6726292 | Obesity (extreme) |
| rs7577894 | Amyotrophic lateral sclerosis |
| rs3791679 | Height |
| rs3791675 | Height |
| rs6751715 | HIV-1 control |
| rs2312147 | Schizophrenia |
| rs887912 | Body mass index,Obesity |
| rs10202231 | Response to antipsychotic treatment |
| rs243021 | Type 2 diabetes |
| rs1427407 | F-cell distribution |
| rs766432 | Beta thalassemia/hemoglobin E disease,Fetal hemoglobin levels,F-cell distribution,Sickle cell anemia (haemolysis) |
| rs11886868 | Fetal hemoglobin levels,F-cell distribution |
| rs1432295 | Hodgkin's lymphoma |
| rs702873 | Psoriasis,Psoriatic arthritis |
| rs842636 | Psoriasis |
| rs13031237 | Rheumatoid arthritis |
| rs13017599 | Psoriatic arthritis,Rheumatoid arthritis |
| rs13003464 | Celiac disease,Crohn's disease,Ulcerative colitis |
| rs7608910 | Ulcerative colitis,Inflammatory bowel disease |
| rs10188217 | Crohn's disease and celiac disease |
| rs10181042 | Crohn's disease |
| rs6545883 | Tuberculosis |
| rs10865331 | Ankylosing spondylitis,Crohn's disease |
| rs721048 | Prostate cancer |
| rs6545977 | Prostate cancer |
| rs7604693 | Kawasaki disease |
| rs934734 | Rheumatoid arthritis |
| rs2300478 | Restless legs syndrome |
| rs724568 | Major depressive disorder (broad) |
| rs9309413 | Metabolite levels |
| rs10496166 | RR interval (heart rate) |
| rs4254535 | Lung cancer |
| rs7577851 | Parkinson's disease (age of onset) |
| rs7595103 | Attention deficit hyperactivity disorder and conduct disorder |
| rs13409348 | Bipolar disorder |

| | |
|---|---|
| rs399885 | Response to antipsychotic treatment |
| rs7570469 | Response to antipsychotic treatment |
| rs11695685 | Protein quantitative trait loci |
| rs1447537 | RR interval (heart rate) |
| rs1534238 | Response to antipsychotic treatment |
| rs7577642 | Protein quantitative trait loci |
| rs12714207 | Bilirubin levels |
| rs11684404 | Height |
| rs4907240 | Event-related brain oscillations |
| rs6733011 | Bipolar disorder |
| rs11676922 | Rheumatoid arthritis |
| rs9653442 | Type 1 diabetes |
| rs10865035 | Rheumatoid arthritis |
| rs2278729 | Osteoporosis |
| rs6711606 | Pancreatic cancer |
| rs2310173 | Ankylosing spondylitis,Autism spectrum disorder, attention deficit-hyperactivity disorder, bipolar disorder, major depressive disorder, and schizophrenia (combined),Ulcerative colitis |
| rs1420101 | Eosinophil counts |
| rs13015714 | Celiac disease,Atopic dermatitis |
| rs3771166 | Asthma |
| rs2058660 | Crohn's disease |
| rs917997 | Celiac disease,Inflammatory bowel disease |
| rs6735786 | Bone mineral density (hip) |
| rs12615966 | Pancreatic cancer |
| rs6712932 | Type 2 diabetes |
| rs1020064 | AIDS |
| rs4676049 | Alzheimer's disease (late onset) |
| rs6542095 | Endometriosis |
| rs11677416 | Working memory |
| rs6734238 | C-reactive protein |
| rs272000 | Attention deficit hyperactivity disorder |
| rs17189298 | T-tau |
| rs2587695 | Attention deficit hyperactivity disorder |
| rs11122834 | Erectile dysfunction and prostate cancer treatment |
| rs1527243 | Erectile dysfunction and prostate cancer treatment |
| rs17367118 | Attention deficit hyperactivity disorder |
| rs1367248 | Tonometry |
| rs2901331 | Response to statin therapy |
| rs13418717 | Heart failure |
| rs7567389 | Self-rated health |
| rs1158867 | Protein C levels |
| rs4662834 | Bleomycin sensitivity |
| rs16831128 | Conduct disorder (symptom count),Conduct disorder (case status) |
| rs1437898 | Multiple sclerosis (age of onset) |
| rs10496702 | Bipolar disorder and schizophrenia |
| rs7577925 | Attention deficit hyperactivity disorder |

| | |
|---|---|
| rs882300 | Electrocardiographic traits,Multiple sclerosis |
| rs1427593 | Pancreatic cancer |
| rs1399645 | Male infertility |
| rs10210358 | Erectile dysfunction and prostate cancer treatment |
| rs12472911 | Menarche (age at onset) |
| rs10928195 | Quantitative traits |
| rs10427255 | Common traits (Other) |
| rs10928302 | HIV-1 control |
| rs7584099 | Response to statin therapy |
| rs10191411 | Protein quantitative trait loci |
| rs2121433 | Alzheimer's disease biomarkers |
| rs11889862 | Menopause (age at onset) |
| rs958672 | Aging traits |
| rs1918172 | Attention deficit hyperactivity disorder |
| rs7601713 | QT interval |
| rs7593730 | Type 2 diabetes |
| rs12476047 | Response to antipsychotic treatment |
| rs13015447 | Amyotrophic lateral sclerosis |
| rs6749447 | Blood pressure |
| rs1402837 | Glycated hemoglobin levels |
| rs560887 | Metabolic syndrome,Metabolite levels,Metabolic traits,Fasting glucose-related traits (interaction with BMI),Fasting plasma glucose,Fasting glucose-related traits |
| rs563694 | Fasting plasma glucose |
| rs552976 | Glycated hemoglobin levels |
| rs830998 | Anorexia nervosa |
| rs2544390 | Urate levels |
| rs4668338 | Uric acid levels |
| rs6433379 | Low-density lipoprotein cholesterol |
| rs7590983 | Anthropometric traits |
| rs4972755 | Bipolar disorder and major depressive disorder (combined) |
| rs2461751 | Electrocardiographic conduction measures |
| rs7566934 | Information processing speed |
| rs2592394 | Magnesium levels |
| rs2072590 | Ovarian cancer |
| rs1529093 | Non-alcoholic fatty liver disease histology (other) |
| rs7602460 | Atrioventricular conduction |
| rs3821236 | Systemic sclerosis,Systemic lupus erythematosus |
| rs7574865 | Systemic sclerosis,Hepatocellular carcinoma (hepatitis B virus related),Celiac disease and Rheumatoid arthritis,Systemic lupus erythematosus,Rheumatoid arthritis,Primary biliary cirrhosis |
| rs10497721 | Diabetes (incident) |
| rs1385351 | HIV-1 control |
| rs700651 | Intracranial aneurysm |
| rs6738825 | Crohn's disease |
| rs7595412 | Hip bone size |
| rs12617311 | Menarche (age at onset) |

| | |
|---|---|
| rs1992950 | Ulcerative colitis |
| rs1569175 | Response to treatment for acute lymphoblastic leukemia |
| rs17385675 | Response to antipsychotic treatment |
| rs1521882 | Attention deficit hyperactivity disorder and conduct disorder |
| rs1376877 | Subclinical atherosclerosis traits (other) |
| rs231735 | Rheumatoid arthritis |
| rs1024161 | Alopecia areata,Graves' disease |
| rs3087243 | Type 1 diabetes,Rheumatoid arthritis,Type 1 diabetes autoantibodies |
| rs4675374 | Celiac disease |
| rs2335704 | Tuberculosis |
| rs1207421 | Knee osteoarthritis |
| rs2277912 | Height |
| rs2242073 | Attention deficit hyperactivity disorder |
| rs12619285 | Eosinophil counts |
| rs3768716 | Neuroblastoma |
| rs6435862 | Neuroblastoma (high-risk) |
| rs7590720 | Alcohol dependence |
| rs13387042 | Breast cancer |
| rs1351164 | Height |
| rs11676348 | Ulcerative colitis |
| rs7349332 | Hair morphology,Common traits (Other) |
| rs6724465 | Height |
| rs10932886 | Brain imaging |
| rs824931 | Body mass index |
| rs1440072 | Waist circumference,Body mass index |
| rs16864755 | Osteoporosis-related phenotypes |
| rs1517484 | Attention deficit hyperactivity disorder (time to onset) |
| rs7578326 | Type 2 diabetes |
| rs2943634 | Fasting insulin-related traits (interaction with BMI),Coronary heart disease |
| rs2943641 | Type 2 diabetes and other traits |
| rs7558386 | P-tau181p |
| rs3820928 | Pulmonary function |
| rs7591163 | Blood pressure |
| rs6436839 | Reasoning |
| rs13397985 | Chronic lymphocytic leukemia |
| rs7423615 | Crohn's disease |
| rs17275498 | Cognitive performance |
| rs2580816 | Height |
| rs10210302 | Crohn's disease |
| rs3828309 | Crohn's disease |
| rs838705 | Calcium levels |
| rs13394720 | HIV-1 control |
| rs11892031 | Bladder cancer |
| rs2602381 | Attention deficit hyperactivity disorder |
| rs887829 | Cholelithiasis-related traits in sickle cell anemia,Metabolite levels,Metabolic traits,Bilirubin levels |
| rs6742078 | Circulating cell-free DNA,Bilirubin levels |

| | |
|---|---|
| rs579327 | Low-density lipoprotein cholesterol |
| rs12151790 | Osteoporosis |
| rs6736997 | Pancreatic cancer |
| rs2042831 | Low-density lipoprotein cholesterol |
| rs13390159 | Response to statin therapy |
| rs1574192 | Brain imaging in schizophrenia (interaction) |
| rs2953145 | Bipolar disorder |
| rs4676406 | Ulcerative colitis |
| rs12479254 | Brain structure |
| rs10510217 | Total ventricular volume |
| rs2619566 | Amyotrophic lateral sclerosis |
| rs1601875 | Bipolar disorder |
| rs794185 | Multiple sclerosis--Brain Glutamate Levels |
| rs9870680 | Major depressive disorder (broad) |
| rs4591494 | Factor VII |
| rs6807064 | Metabolite levels |
| rs9990174 | Conduct disorder (interaction) |
| rs3729931 | Cardiac hypertrophy |
| rs294636 | Low-density lipoprotein cholesterol |
| rs6442522 | Uric acid levels |
| rs690037 | Optic disc size (cup) |
| rs12635698 | Obesity (extreme) |
| rs13073817 | Crohn's disease |
| rs6809854 | Psoriasis |
| rs2053506 | Hip geometry |
| rs11720452 | Bipolar disorder |
| rs1490157 | Partial epilepsies |
| rs2593321 | HIV-1 control |
| rs9310709 | Chronic kidney disease and serum creatinine levels |
| rs9310736 | Mean corpuscular hemoglobin,Mean corpuscular volume,Red blood cell traits |
| rs993804 | Bipolar disorder and schizophrenia |
| rs7617877 | Parkinson's disease |
| rs3773643 | Tonometry |
| rs1902341 | Peripheral artery disease |
| rs4380451 | Bipolar disorder |
| rs9845475 | Attention deficit hyperactivity disorder (time to onset) |
| rs13314993 | Celiac disease |
| rs4678680 | Hepatocellular carcinoma |
| rs7631605 | P-tau181p |
| rs9311171 | Prostate cancer |
| rs2070488 | Electrocardiographic conduction measures |
| rs11129795 | Electrocardiographic traits,QT interval |
| rs12053903 | Electrocardiographic traits,QT interval |
| rs7638909 | Electrocardiographic conduction measures |
| rs3922844 | PR interval |
| rs11708996 | Ventricular conduction,PR interval |
| rs11710077 | Ventricular conduction |

| | |
|---|---|
| rs9851724 | Ventricular conduction |
| rs6801957 | Ventricular conduction,PR interval |
| rs6800541 | Atrioventricular conduction,PR interval |
| rs816488 | Cognitive performance |
| rs6599077 | Sleep duration |
| rs10490823 | Bone mineral density (hip) |
| rs87938 | Bone mineral density (hip) |
| rs9815354 | Diastolic blood pressure |
| rs9990343 | Brain structure |
| rs6441961 | Celiac disease |
| rs7617480 | Menarche (age at onset) |
| rs6762477 | Menarche (age at onset) |
| rs6784615 | Waist-hip ratio |
| rs2336725 | Height |
| rs4687718 | Ventricular conduction |
| rs358806 | Type 2 diabetes |
| rs1795648 | Bipolar disorder and schizophrenia |
| rs2054989 | Hip geometry |
| rs12485738 | Mean platelet volume |
| rs2062583 | Rheumatoid arthritis |
| rs6445975 | Systemic lupus erythematosus |
| rs1825630 | Aortic root size |
| rs652889 | QT interval |
| rs153734 | Folate pathway vitamin levels |
| rs6795735 | Age-related macular degeneration,Waist-hip ratio |
| rs4607103 | Type 2 diabetes |
| rs11709625 | Hematological and biochemical traits |
| rs922948 | Hip geometry |
| rs7647307 | Brain imaging |
| rs1024889 | Body mass index |
| rs17651978 | Attention deficit hyperactivity disorder |
| rs9836484 | Brain imaging in schizophrenia (interaction) |
| rs9831754 | Calcium levels |
| rs9834373 | Protein quantitative trait loci |
| rs13078807 | Body mass index,Obesity |
| rs7642134 | Menarche (age at onset) |
| rs2660753 | Prostate cancer |
| rs17181170 | Prostate cancer |
| rs2880961 | Response to acetaminophen (hepatotoxicity) |
| rs4390941 | Low-density lipoprotein cholesterol |
| rs13095226 | Age-related macular degeneration |
| rs991258 | Hip geometry |
| rs9657904 | Multiple sclerosis |
| rs4450776 | Cognitive performance |
| rs6438424 | Menarche (age at onset) |
| rs11712165 | Celiac disease |
| rs3772130 | Cognitive performance |

| | |
|---|---|
| rs2650951 | QT interval |
| rs11708067 | Fasting glucose-related traits (interaction with BMI),Type 2 diabetes,Fasting glucose-related traits |
| rs2877716 | Two-hour glucose challenge |
| rs9883204 | Birth weight |
| rs9871760 | MRI atrophy measures |
| rs2687729 | Menarche (age at onset) |
| rs10934853 | Prostate cancer |
| rs4857855 | Eosinophil counts |
| rs6439167 | Height |
| rs6439371 | Menarche (age at onset) |
| rs2718812 | Iron status biomarkers |
| rs1867504 | Iron status biomarkers |
| rs3811647 | Iron levels,Alcohol consumption (transferrin glycosylation),Hepcidin levels,Iron status biomarkers |
| rs1830084 | Iron status biomarkers |
| rs10935120 | Height |
| rs511154 | Fibrinogen |
| rs10935268 | Acenocoumarol maintenance dosage |
| rs9846480 | MRI atrophy measures |
| rs1511412 | Keloid |
| rs908821 | Multiple sclerosis |
| rs6440003 | Height |
| rs6763931 | Height,Prostate cancer |
| rs724016 | Height |
| rs347685 | Chronic kidney disease |
| rs894177 | Non-alcoholic fatty liver disease histology (other) |
| rs9810857 | Attention deficit hyperactivity disorder |
| rs2800 | Non-alcoholic fatty liver disease histology (other) |
| rs800082 | Smoking behavior |
| rs345013 | Prostate cancer |
| rs1841770 | Multiple sclerosis |
| rs6798928 | Immunoglobulin A |
| rs3772255 | Aging traits |
| rs2665390 | Ovarian cancer in BRCA1 mutation carriers,Ovarian cancer |
| rs12638253 | Multiple sclerosis (severity) |
| rs900400 | Birth weight |
| rs2222328 | Quantitative traits |
| rs6441286 | Primary biliary cirrhosis |
| rs6808138 | Hyperactive-impulsive symptoms |
| rs7427021 | Bipolar disorder |
| rs1523288 | Heart failure |
| rs448378 | Magnesium levels,Systolic blood pressure |
| rs1918974 | Diastolic blood pressure |
| rs12696304 | Telomere length |
| rs6444931 | Bipolar disorder and schizophrenia |
| rs4955755 | Menopause (age at onset) |

| | |
|---|---|
| rs2088885 | Brain imaging in schizophrenia (interaction) |
| rs13074924 | Major depressive disorder |
| rs17531088 | Kawasaki disease |
| rs6799767 | Eosinophilic esophagitis (pediatric) |
| rs644695 | Major depressive disorder (broad) |
| rs9290663 | Acute lymphoblastic leukemia (childhood) |
| rs6782299 | Schizophrenia |
| rs11711441 | Parkinson's disease |
| rs683395 | Bipolar disorder |
| rs7647854 | Major depressive disorder |
| rs7374394 | Cognitive performance |
| rs4402960 | Type 2 diabetes |
| rs1470579 | Fasting glucose-related traits (interaction with BMI),Diabetes (gestational),Type 2 diabetes |
| rs6769511 | Type 2 diabetes |
| rs720390 | Height |
| rs6444087 | Smoking behavior |
| rs2002675 | Menarche (age at onset) |
| rs10049246 | Attention deficit hyperactivity disorder symptoms (interaction) |
| rs7647305 | Weight,Body mass index |
| rs9816226 | Body mass index,Obesity |
| rs11924390 | Adiponectin levels |
| rs266717 | Adiponectin levels |
| rs1648707 | Adiponectin levels |
| rs864265 | Adiponectin levels |
| rs17366568 | Adiponectin levels |
| rs10937275 | Drug-induced liver injury (flucloxacillin) |
| rs1152846 | Weight,Body mass index |
| rs7610017 | Brain imaging |
| rs10937405 | Lung adenocarcinoma |
| rs7626795 | Lung cancer |
| rs10937470 | Total ventricular volume |
| rs975121 | Ileal carcinoids |
| rs4453795 | Metabolite levels |
| rs789852 | QT interval |
| rs3892715 | Attention deficit hyperactivity disorder (time to onset) |
| rs2131877 | Non-small cell lung cancer |
| rs9859260 | Mean corpuscular volume |
| rs11924930 | HIV-1 susceptibility |
| rs2084385 | Total ventricular volume |
| rs1564282 | Parkinson's disease (familial) |
| rs11248051 | Parkinson's disease |
| rs6599388 | Parkinson's disease |
| rs11248060 | Parkinson's disease |
| rs1670533 | Recombination rate (females) |
| rs3796619 | Recombination rate (males) |
| rs798766 | Urinary bladder cancer,Bladder cancer |

| | |
|---|---|
| rs2008242 | Electrocardiographic conduction measures |
| rs4689388 | Type 2 diabetes and other traits |
| rs4234798 | Insulin-like growth factors |
| rs734553 | Uric acid levels |
| rs13129697 | Uric acid levels,Urate levels,Biochemical measures |
| rs737267 | Urate levels |
| rs6855911 | Urate levels |
| rs7442295 | Urate levels |
| rs3775948 | Uric acid levels,Renal function-related traits (urea) |
| rs717615 | Uric acid levels |
| rs11724635 | Parkinson's disease |
| rs4698412 | Parkinson's disease |
| rs4538475 | Parkinson's disease |
| rs7678436 | Height |
| rs16896068 | Height |
| rs6830062 | Height |
| rs666088 | Echocardiographic traits |
| rs358592 | Suicidal ideation |
| rs3816587 | Rheumatoid arthritis |
| rs959903 | Non-alcoholic fatty liver disease histology (other) |
| rs10517086 | Type 1 diabetes |
| rs874040 | Rheumatoid arthritis |
| rs4692256 | Brain imaging |
| rs17390445 | Response to antipsychotic treatment |
| rs437943 | Response to TNF antagonist treatment |
| rs1533317 | QT interval |
| rs13117816 | Multiple sclerosis--Brain Glutamate Levels |
| rs10938397 | Body mass index,Obesity |
| rs10938494 | Sphingolipid levels |
| rs2898681 | Optic disc size (cup) |
| rs17690232 | Height |
| rs218237 | Mean corpuscular hemoglobin,Mean corpuscular volume,Red blood cell count |
| rs172629 | Mean corpuscular volume |
| rs2537859 | Bipolar disorder |
| rs6832769 | Personality dimensions |
| rs708547 | Bleomycin sensitivity |
| rs10517437 | Cognitive performance |
| rs10517480 | Metabolite levels |
| rs4599440 | Biochemical measures |
| rs6856328 | Working memory |
| rs2172802 | Partial epilepsies |
| rs1155865 | Cognitive test performance |
| rs2242330 | Parkinson's disease |
| rs7697556 | Height |
| rs1350666 | Attention deficit hyperactivity disorder |
| rs6812193 | Parkinson's disease |
| rs9992101 | Creatinine levels |

| | |
|---|---|
| rs17319721 | Renal function and chronic kidney disease,Chronic kidney disease |
| rs13146355 | Renal function-related traits (sCR),Magnesium levels,Renal function-related traits (eGRFcrea) |
| rs13106227 | Eosinophilic esophagitis (pediatric) |
| rs1986734 | Eosinophilic esophagitis (pediatric) |
| rs1268789 | Hair morphology |
| rs4333130 | Ankylosing spondylitis |
| rs7692808 | PR interval |
| rs7660702 | Electrocardiographic traits |
| rs442177 | Triglycerides |
| rs1471403 | Bone mineral density (hip),Bone mineral density (spine) |
| rs893971 | Conduct disorder (interaction) |
| rs2869967 | Pulmonary function,Pulmonary function (interaction) |
| rs7671167 | Chronic obstructive pulmonary disease |
| rs356219 | Parkinson's disease |
| rs11931074 | Parkinson's disease |
| rs356220 | Parkinson's disease |
| rs2736990 | Parkinson's disease |
| rs6532197 | Parkinson's disease |
| rs11097407 | Bipolar disorder and schizophrenia |
| rs12500426 | Prostate cancer |
| rs17021918 | Prostate cancer |
| rs12643654 | P-tau181p |
| rs10516430 | Biochemical measures |
| rs991316 | Hypertension |
| rs11735070 | Response to antipsychotic treatment |
| rs1405687 | Response to antipsychotic treatment |
| rs4699052 | Testicular germ cell tumor |
| rs7679673 | Prostate cancer |
| rs7669317 | Response to antipsychotic therapy (extrapyramidal side effects) |
| rs10516541 | Mean forced vital capacity from 2 exams |
| rs10033900 | Age-related macular degeneration,Age-related macular degeneration (CNV),Age-related macular degeneration (GA) |
| rs6847149 | Exercise treadmill test traits |
| rs10011926 | Attention deficit hyperactivity disorder |
| rs6843082 | Atrial fibrillation,Stroke (ischemic) |
| rs10033464 | Atrial fibrillation/atrial flutter |
| rs10433903 | Obesity (extreme) |
| rs4318720 | QT interval |
| rs180730 | Fasting plasma glucose |
| rs7659604 | Type 2 diabetes |
| rs6534347 | Type 1 diabetes |
| rs7682241 | Alopecia areata |
| rs1395241 | Bipolar disorder and major depressive disorder (combined) |
| rs12639834 | Cognitive performance |
| rs950063 | Smoking behavior |
| rs1320267 | Subclinical atherosclerosis traits (other) |

| | |
|---|---|
| rs1024020 | RR interval (heart rate) |
| rs1478091 | Multiple sclerosis (severity) |
| rs2313132 | Systemic lupus erythematosus |
| rs17050782 | Smoking behavior |
| rs1550057 | Conduct disorder (case status) |
| rs17007017 | Conduct disorder (case status) |
| rs12504628 | Pulmonary function |
| rs13147758 | Pulmonary function |
| rs1828591 | Chronic obstructive pulmonary disease |
| rs1980057 | Pulmonary function,Pulmonary function (interaction) |
| rs13118928 | Chronic obstructive pulmonary disease |
| rs1812175 | Height |
| rs6854783 | Height |
| rs1492820 | Height |
| rs6845865 | QT interval |
| rs1490453 | Biochemical measures |
| rs2358462 | Non-alcoholic fatty liver disease histology (other) |
| rs360929 | Volumetric brain MRI |
| rs11099864 | Amyotrophic lateral sclerosis |
| rs17030434 | Electrocardiographic conduction measures |
| rs1800789 | Fibrinogen |
| rs727153 | Alzheimer's disease |
| rs4234898 | Speech perception in dyslexia |
| rs17291045 | HIV-1 control |
| rs4470583 | Diabetic retinopathy |
| rs17638464 | Height |
| rs2710833 | Non-alcoholic fatty liver disease histology (AST) |
| rs17067123 | Response to hepatitis C treatment |
| rs2383393 | Body mass index |
| rs7672826 | Multiple sclerosis |
| rs1431005 | Response to statin therapy |
| rs7659062 | Cognitive performance |
| rs11739663 | Ulcerative colitis |
| rs4580814 | mean corpuscular hemoglobin concentration |
| rs2736100 | Glioma,Interstitial lung disease,Lung adenocarcinoma,Idiopathic pulmonary fibrosis,Red blood cell count,Testicular germ cell cancer,Lung cancer |
| rs2853676 | Glioma |
| rs4635969 | Testicular germ cell cancer,Testicular germ cell tumor |
| rs4975616 | Lung cancer |
| rs402710 | Lung cancer |
| rs401681 | Melanoma,Prostate-specific antigen levels,Pancreatic cancer,Lung cancer,Bladder cancer |
| rs31489 | Lung adenocarcinoma |
| rs12653946 | Prostate cancer |
| rs6879627 | Pancreatic cancer |
| rs17586674 | Cognitive performance |
| rs11748327 | Myocardial infarction |

| | |
|---|---|
| rs7727102 | Bipolar disorder and schizophrenia |
| rs32566 | Morbidity-free survival |
| rs11134178 | Attention deficit hyperactivity disorder |
| rs7702187 | Parkinson's disease |
| rs267939 | Ulcerative colitis |
| rs6885224 | Myopia (pathological) |
| rs2896103 | Subclinical atherosclerosis traits (other) |
| rs7715811 | Subclinical atherosclerosis traits (other) |
| rs1502050 | Subclinical atherosclerosis traits (other) |
| rs682748 | Hippocampal atrophy |
| rs349475 | Depression (quantitative trait) |
| rs4701252 | Waist circumference |
| rs2047267 | Uric acid levels |
| rs7727656 | Hippocampal atrophy |
| rs10038113 | Autism |
| rs4307059 | Autism |
| rs4479806 | Anorexia nervosa |
| rs2548003 | Hip geometry |
| rs9292394 | Emphysema-related traits |
| rs10472828 | Height |
| rs6868223 | Mortality among heart failure patients |
| rs35391 | Tanning |
| rs28777 | Black vs. blond hair color,Black vs. red hair color |
| rs409045 | Left ventricular mass |
| rs267759 | Response to treatment for acute lymphoblastic leukemia |
| rs11959928 | Chronic kidney disease |
| rs11742570 | Crohn's disease,Inflammatory bowel disease |
| rs6451493 | Ulcerative colitis |
| rs1992660 | Crohn's disease |
| rs6896969 | Multiple sclerosis |
| rs1373692 | Crohn's disease |
| rs9292777 | Crohn's disease,Multiple sclerosis |
| rs979233 | Systemic lupus erythematosus |
| rs13188386 | Iron status biomarkers |
| rs4296809 | Brain structure |
| rs4415084 | Breast cancer |
| rs7716600 | Breast cancer |
| rs10941694 | Chronic kidney disease and serum creatinine levels |
| rs981782 | Breast cancer |
| rs415407 | Low-density lipoprotein cholesterol |
| rs6859219 | Rheumatoid arthritis |
| rs16886165 | Breast cancer |
| rs889312 | Breast cancer |
| rs1844437 | Hoarding |
| rs1823068 | Sleepiness |
| rs702543 | Neuroticism |
| rs10514995 | RR interval (heart rate) |

| | |
|---|---|
| rs10069397 | Low-density lipoprotein cholesterol |
| rs2199161 | Attention deficit hyperactivity disorder |
| rs10515148 | Hip geometry |
| rs7702331 | Crohn's disease |
| rs7703051 | LDL cholesterol |
| rs12654264 | Metabolite levels,LDL cholesterol |
| rs3846662 | Cholesterol, total,LDL cholesterol |
| rs3846663 | Quantitative traits,LDL cholesterol |
| rs2112347 | Body mass index,Obesity |
| rs457717 | Hearing impairment |
| rs4457053 | Type 2 diabetes |
| rs6881634 | Hippocampal atrophy |
| rs337847 | Hippocampal atrophy |
| rs2052550 | Iron status biomarkers |
| rs10078095 | Height |
| rs7713917 | Major depressive disorder |
| rs410644 | Anorexia nervosa |
| rs1032757 | Eosinophilic esophagitis (pediatric) |
| rs310501 | Major depressive disorder |
| rs4466137 | Prostate cancer |
| rs347344 | Chemerin levels |
| rs7717527 | Low-density lipoprotein cholesterol |
| rs4920799 | Echocardiographic traits |
| rs770189 | Tonometry |
| rs10037512 | Height |
| rs1366594 | Bone mineral density (hip),Bone mineral density |
| rs17376456 | Diabetic retinopathy |
| rs17418283 | Bipolar disorder |
| rs27524 | Hodgkin's lymphoma,Psoriasis |
| rs2549794 | Crohn's disease |
| rs4703129 | Asperger disorder |
| rs1829883 | Hemostatic factors and hematological phenotypes |
| rs10067427 | Non-alcoholic fatty liver disease histology (lobular) |
| rs1502844 | Schizophrenia |
| rs26232 | Rheumatoid arthritis |
| rs252817 | Bipolar disorder and schizophrenia |
| rs10074258 | Hippocampal atrophy |
| rs4460176 | Hemostatic factors and hematological phenotypes |
| rs3806932 | Eosinophilic esophagitis (pediatric) |
| rs2416257 | Eosinophil counts |
| rs17470137 | Aortic root size |
| rs2115172 | Weight |
| rs4836133 | Body mass index |
| rs2637496 | HIV-1 control |
| rs245201 | Brain imaging in schizophrenia (interaction) |
| rs374748 | Obesity (extreme) |
| rs274546 | Height |

| | |
|---|---|
| rs2073643 | Asthma |
| rs6596075 | Crohn's disease |
| rs1016988 | Fibrinogen |
| rs2522056 | Fibrinogen |
| rs4705952 | C-reactive protein |
| rs4143832 | Eosinophil counts |
| rs2244012 | Asthma |
| rs2040704 | IgE levels |
| rs1295686 | Asthma,Atopic dermatitis |
| rs17166496 | Type 1 diabetes |
| rs1644305 | Attention deficit hyperactivity disorder and conduct disorder |
| rs13187289 | Menarche (age at onset) |
| rs31198 | Height |
| rs757647 | Menarche (age at onset) |
| rs11167764 | Crohn's disease |
| rs4624820 | Testicular germ cell cancer,Testicular germ cell tumor |
| rs152528 | Cardiac hypertrophy |
| rs3776331 | Uric acid levels |
| rs9325032 | Cognitive test performance |
| rs11168048 | Pulmonary function,Pulmonary function (interaction) |
| rs3995090 | Pulmonary function |
| rs32579 | Tanning |
| rs2304069 | HIV-1 control |
| rs10036748 | Systemic lupus erythematosus |
| rs1010254 | Optic disc size (cup) |
| rs12658202 | Anthropometric traits |
| rs13165478 | Ventricular conduction |
| rs4704970 | Multiple sclerosis (age of onset) |
| rs157350 | Anthropometric traits |
| rs1501908 | LDL cholesterol |
| rs2277027 | Pulmonary function,Pulmonary function (interaction) |
| rs1473247 | Mean platelet volume |
| rs2546890 | Psoriasis,Multiple sclerosis |
| rs10045431 | Crohn's disease |
| rs6887695 | Crohn's disease |
| rs6556756 | Breast cancer |
| rs958994 | Carotid atherosclerosis in HIV infection |
| rs2122554 | Conduct disorder (symptom count) |
| rs4282339 | Height |
| rs13156607 | Waist circumference |
| rs169082 | Protein quantitative trait loci |
| rs254893 | Quantitative traits |
| rs251253 | PR interval |
| rs889014 | Height |
| rs359457 | Crohn's disease |
| rs6861681 | Waist-hip ratio |
| rs10475598 | Information processing speed |

| | |
|---|---|
| rs2731672 | Metabolite levels,Circulating vasoactive peptide levels,Activated partial thromboplastin time,Platelet function and related traits |
| rs10039254 | Attention deficit hyperactivity disorder (time to onset) |
| rs10464059 | Parkinson's disease |
| rs655601 | Quantitative traits |
| rs9378805 | Chronic lymphocytic leukemia |
| rs1540771 | Freckles |
| rs6918152 | Black vs. blond hair color,Black vs. red hair color |
| rs9502893 | Pancreatic cancer |
| rs4959235 | Antipsychotic-induced QTc interval prolongation |
| rs17309827 | Crohn's disease |
| rs1294421 | Waist-hip ratio |
| rs675209 | Urate levels |
| rs11755724 | Age-related macular degeneration,Multiple sclerosis |
| rs12198986 | Height |
| rs10484246 | Morbidity-free survival |
| rs2153157 | Menopause (age at onset),Menarche and menopause (age at onset) |
| rs1040994 | Response to antipsychotic treatment |
| rs12526453 | Myocardial infarction (early onset),Coronary heart disease |
| rs4715166 | Hip geometry |
| rs499818 | Major CVD |
| rs6914079 | Cognitive test performance |
| rs6941421 | Multiple sclerosis (severity) |
| rs697739 | Amyotrophic lateral sclerosis |
| rs12199222 | Height |
| rs41441749 | Hyperactive-impulsive symptoms |
| rs1202199 | Hyperactive-impulsive symptoms |
| rs6908425 | Crohn's disease |
| rs4712653 | Neuroblastoma |
| rs6939340 | Neuroblastoma |
| rs4236016 | Cardiac hypertrophy |
| rs6922632 | Information processing speed |
| rs793834 | Information processing speed |
| rs932316 | Iron status biomarkers |
| rs1183201 | Uric acid levels |
| rs1165205 | Urate levels |
| rs12216125 | Iron status biomarkers |
| rs198846 | Hemoglobin,Mean corpuscular volume,Blood pressure |
| rs10946808 | Height |
| rs9271366 | Immunoglobulin A,Ulcerative colitis,Multiple sclerosis,Inflammatory bowel disease |
| rs999943 | Obesity (extreme) |
| rs2274459 | Obesity (extreme) |
| rs1776897 | Height |
| rs6918981 | Height |
| rs2814993 | Height |
| rs4713858 | Height |

| | |
|---|---|
| rs1321311 | Colorectal cancer,Electrocardiographic traits |
| rs9470361 | Ventricular conduction |
| rs904251 | Cognitive performance |
| rs9471576 | Total ventricular volume |
| rs2842643 | Attention deficit hyperactivity disorder |
| rs3218097 | Mean corpuscular hemoglobin,Mean corpuscular volume,Red blood cell count |
| rs9349205 | Mean corpuscular hemoglobin,Mean corpuscular volume |
| rs6458307 | Bipolar disorder |
| rs6905288 | Waist-hip ratio,Coronary heart disease |
| rs943072 | Ulcerative colitis |
| rs881858 | Chronic kidney disease |
| rs9472138 | Type 2 diabetes,Thyroid hormone levels |
| rs3799977 | Attention deficit hyperactivity disorder |
| rs10948197 | Height |
| rs2103868 | Panic disorder |
| rs9473582 | Brain structure |
| rs987237 | Body mass index,Adiposity |
| rs1555967 | Waist circumference |
| rs735860 | Glaucoma |
| rs79505489 | Eosinophilic esophagitis (pediatric) |
| rs6455128 | Protein quantitative trait loci |
| rs682238 | Calcium levels |
| rs9354308 | Metabolite levels |
| rs11970254 | Brain structure |
| rs1727638 | AB1-42 |
| rs346291 | Partial epilepsies |
| rs493187 | Bipolar disorder and schizophrenia |
| rs1925690 | MRI atrophy measures |
| rs2509458 | Blood pressure |
| rs10806425 | Celiac disease |
| rs3757247 | Type 1 diabetes,Vitiligo |
| rs11755527 | Type 1 diabetes,Type 1 diabetes autoantibodies |
| rs1847472 | Crohn's disease,Inflammatory bowel disease |
| rs806276 | Attention deficit hyperactivity disorder (time to onset) |
| rs713155 | Brain structure |
| rs2506933 | Cognitive performance |
| rs6909430 | Quantitative traits |
| rs4840086 | Menarche (age at onset) |
| rs2841307 | Bipolar disorder and schizophrenia |
| rs9377619 | Bipolar disorder and schizophrenia |
| rs9322817 | Thyroid stimulating hormone |
| rs7759938 | Menarche (age at onset),Height,Pubertal anthropometrics |
| rs314280 | Menarche (age at onset) |
| rs314277 | Height,Digit length ratio,Menarche and menopause (age at onset) |
| rs314276 | Menarche (age at onset) |
| rs314268 | Height |
| rs9386463 | Primary tooth development (time to first tooth eruption) |

| | |
|---|---|
| rs7746082 | Crohn's disease |
| rs6911490 | Ulcerative colitis |
| rs548234 | Systemic lupus erythematosus |
| rs1417352 | Select biomarker traits |
| rs2153960 | Insulin-like growth factors |
| rs9487094 | Height |
| rs9400317 | Response to hepatitis C treatment |
| rs240993 | Psoriasis |
| rs9488363 | Optic disc size (rim) |
| rs339331 | Prostate cancer |
| rs281868 | Resting heart rate |
| rs89107 | Cardiac structure and function |
| rs11153730 | Ventricular conduction,Heart rate,QT interval |
| rs11970286 | Electrocardiographic traits,QT interval |
| rs11756438 | QT interval |
| rs2817937 | Primary tooth development (number of teeth) |
| rs1343075 | Cognitive performance |
| rs11154022 | Resting heart rate |
| rs61464641 | Protein quantitative trait loci |
| rs9398652 | Resting heart rate |
| rs12110693 | Biomedical quantitative traits |
| rs9491140 | Neuroticism |
| rs9388489 | Type 1 diabetes |
| rs1361108 | Menarche (age at onset),Height |
| rs1490388 | Height |
| rs4549631 | Height |
| rs972275 | Iron status biomarkers |
| rs2180341 | Breast cancer |
| rs6569648 | Height |
| rs6899976 | Height |
| rs9402515 | Protein quantitative trait loci |
| rs2049953 | Non-alcoholic fatty liver disease histology (AST) |
| rs7776054 | Mean corpuscular hemoglobin |
| rs9399137 | HbA2 levels,Platelet counts,Mean corpuscular hemoglobin,Mean corpuscular volume,Hematology traits,F-cell distribution |
| rs9373124 | Other erythrocyte phenotypes,White blood cell types |
| rs4895441 | Mean corpuscular hemoglobin concentration,Mean corpuscular volume,White blood cell count |
| rs9376092 | Beta thalassemia/hemoglobin E disease |
| rs9402686 | Hematological parameters |
| rs9494145 | Red blood cell traits,Mean platelet volume |
| rs9483788 | Other erythrocyte phenotypes,Hematocrit |
| rs6569992 | Red blood cell traits |
| rs2327832 | Celiac disease |
| rs10499194 | Rheumatoid arthritis |
| rs6920220 | Ulcerative colitis,Rheumatoid arthritis,Inflammatory bowel disease |
| rs610604 | Psoriasis |

| | |
|---|---|
| rs628751 | Mean corpuscular hemoglobin |
| rs643381 | Mean corpuscular volume |
| rs11155133 | Acute lymphoblastic leukemia (childhood) |
| rs225717 | Retinal vascular caliber |
| rs6570507 | Scoliosis,Height |
| rs4896582 | Height |
| rs3748069 | Height |
| rs9373523 | Tuberculosis |
| rs9390537 | Alzheimer's disease |
| rs6930576 | Type 2 diabetes nephropathy |
| rs2500535 | Response to antidepressants |
| rs1853665 | Radiation response |
| rs9479482 | Alopecia areata |
| rs9372078 | Panic disorder |
| rs6922269 | Coronary heart disease |
| rs4870044 | Bone mineral density (hip),Bone mineral density (spine) |
| rs3757318 | Breast cancer |
| rs1038304 | Bone mineral density (hip),Bone mineral density (spine) |
| rs2046210 | Breast Cancer in BRCA1 mutation carriers,Breast cancer |
| rs543650 | Height |
| rs6902771 | Alcohol dependence |
| rs1322512 | Tonometry |
| rs9478751 | Uric acid levels |
| rs212388 | Crohn's disease and celiac disease,Crohn's disease |
| rs651164 | Prostate cancer |
| rs6919346 | Lp (a) levels |
| rs7770628 | Protein quantitative trait loci |
| rs992037 | Metabolite levels |
| rs3016539 | Pancreatic cancer |
| rs10945919 | Response to TNF antagonist treatment |
| rs4709845 | Major depressive disorder |
| rs7762160 | Conduct disorder (symptom count) |
| rs2236313 | Vitiligo |
| rs2301436 | Crohn's disease |
| rs3093024 | Rheumatoid arthritis |
| rs3093023 | Rheumatoid arthritis |
| rs13208776 | Vitiligo |
| rs3734905 | HIV-1 control |
| rs6952808 | Bipolar disorder and schizophrenia |
| rs1107592 | Autism spectrum disorder, attention deficit-hyperactivity disorder, bipolar disorder, major depressive disorder, and schizophrenia (combined),Bipolar disorder and schizophrenia |
| rs798544 | Height |
| rs798502 | Ulcerative colitis |
| rs798497 | Height |
| rs1182188 | Height |
| rs6462411 | Quantitative traits |

| | |
|---|---|
| rs10488360 | Factor VII |
| rs10259085 | Multiple sclerosis (severity) |
| rs7780564 | Insulin-like growth factors |
| rs2349775 | Neuroticism |
| rs6463843 | Brain imaging |
| rs1285407 | Protein quantitative trait loci |
| rs6971925 | Response to treatment for acute lymphoblastic leukemia |
| rs10244051 | Metabolic traits |
| rs2191349 | Fasting glucose-related traits (interaction with BMI),Fasting glucose-related traits |
| rs10486776 | Stroke |
| rs10270805 | Response to statin therapy |
| rs2709736 | Bipolar disorder |
| rs12670798 | Cholesterol, total,LDL cholesterol |
| rs1175000 | Height |
| rs2097677 | C-reactive protein |
| rs12700667 | Endometriosis |
| rs886716 | Smoking behavior |
| rs10486567 | Prostate cancer |
| rs864745 | Crohn's disease,Type 2 diabetes |
| rs849141 | Height |
| rs1635852 | Height |
| rs849134 | Type 2 diabetes |
| rs2237349 | Attention deficit hyperactivity disorder |
| rs2252521 | Cognitive performance |
| rs215614 | Smoking behavior |
| rs1362212 | Ventricular conduction |
| rs2392362 | Information processing speed |
| rs343064 | Non-alcoholic fatty liver disease histology (other) |
| rs6948404 | HIV-1 control |
| rs741301 | Type 2 diabetes nephropathy |
| rs10488031 | QT interval |
| rs6974491 | Celiac disease,Primary biliary cirrhosis |
| rs1524058 | Bone mineral density (spine) |
| rs29880 | Iron status biomarkers |
| rs1079866 | Menarche (age at onset) |
| rs10486715 | Quantitative traits |
| rs730497 | Glycated hemoglobin levels |
| rs1799884 | Glycemic traits,Metabolite levels,Glycated hemoglobin levels |
| rs4607517 | Fasting glucose-related traits (interaction with BMI),Fasting plasma glucose,Fasting glucose-related traits |
| rs11977526 | Insulin-like growth factors |
| rs1486139 | Select biomarker traits |
| rs7784776 | Ventricular conduction |
| rs700752 | Insulin-like growth factors |
| rs7805803 | Total ventricular volume |
| rs1456893 | Crohn's disease |

| | |
|---|---|
| rs10276619 | Systemic lupus erythematosus,Hippocampal atrophy |
| rs12718597 | Mean corpuscular volume |
| rs11978267 | Acute lymphoblastic leukemia (childhood) |
| rs1451375 | Malaria |
| rs4948088 | Type 1 diabetes |
| rs10266483 | Response to statin therapy |
| rs6961611 | Information processing speed |
| rs2240466 | Caffeine consumption,Triglycerides |
| rs1178979 | Triglycerides |
| rs714052 | Triglycerides,Hypertriglyceridemia |
| rs17145713 | Protein C levels |
| rs13233571 | C-reactive protein |
| rs17145738 | HDL cholesterol,Triglycerides |
| rs1167796 | Systemic lupus erythematosus |
| rs6465825 | Chronic kidney disease |
| rs2691543 | Height |
| rs10499859 | Left ventricular mass |
| rs4236644 | Bilirubin levels |
| rs10954668 | Non-alcoholic fatty liver disease histology (lobular) |
| rs2371208 | Aging traits |
| rs42041 | Rheumatoid arthritis |
| rs2040494 | Height |
| rs2282978 | Height |
| rs445 | White blood cell count,White blood cell types |
| rs441051 | Mean forced vital capacity from 2 exams |
| rs854555 | Response to TNF antagonist treatment |
| rs4729260 | Bone mineral density (spine) |
| rs7781370 | Bone mineral density (hip) |
| rs12669076 | Immunoglobulin A |
| rs17598306 | Radiation response |
| rs6465657 | Prostate cancer |
| rs112817486 | Ulcerative colitis |
| rs7792939 | Anthropometric traits |
| rs7786877 | Mean corpuscular volume |
| rs7385804 | Iron levels,Hematocrit,Hematological parameters |
| rs2075671 | Other erythrocyte phenotypes |
| rs314370 | Resting heart rate |
| rs7341475 | Schizophrenia |
| rs3914132 | Otosclerosis |
| rs4298437 | Alzheimer's disease |
| rs13224682 | Response to antipsychotic treatment |
| rs2108225 | Ulcerative colitis |
| rs4730273 | Ulcerative colitis |
| rs4730276 | Ulcerative colitis |
| rs4510766 | Ulcerative colitis |
| rs886774 | Ulcerative colitis |
| rs4598195 | Ulcerative colitis |

| | |
|---|---|
| rs2158836 | Ulcerative colitis |
| rs6968385 | Attention deficit hyperactivity disorder (time to onset) |
| rs10279573 | Cognitive performance |
| rs12531640 | Low-density lipoprotein cholesterol |
| rs10229603 | Attention deficit hyperactivity disorder and conduct disorder |
| rs10953730 | Metabolite levels |
| rs4236601 | Glaucoma (primary open-angle) |
| rs3807989 | Atrial fibrillation,Electrocardiographic traits,PR interval |
| rs7782376 | Cognitive performance |
| rs7776725 | Biomedical quantitative traits |
| rs11971186 | Chemerin levels |
| rs4728142 | Systemic lupus erythematosus,Ulcerative colitis |
| rs12537284 | Systemic lupus erythematosus |
| rs7787531 | Tuberculosis |
| rs972283 | Type 2 diabetes |
| rs10265216 | Major depressive disorder (broad) |
| rs12534221 | AB1-42 |
| rs11761231 | Rheumatoid arthritis |
| rs357394 | Pulmonary function |
| rs1874326 | Quantitative traits |
| rs12531488 | Brain imaging |
| rs2214681 | Bone mineral density |
| rs7807268 | Crohn's disease |
| rs855913 | Amyotrophic lateral sclerosis |
| rs2968864 | QT interval |
| rs2968863 | QT interval |
| rs7795096 | Bipolar disorder (age of onset and psychomotor symptoms) |
| rs6464375 | Pancreatic cancer |
| rs10260404 | Amyotrophic lateral sclerosis |
| rs10949808 | Bipolar disorder and schizophrenia |
| rs6966038 | Response to citalopram treatment |
| rs2527866 | Quantitative traits |
| rs10227331 | Inattentive symptoms |
| rs6459804 | Bipolar disorder and schizophrenia |
| rs2730245 | Height |
| rs1529316 | Multiple sclerosis |
| rs1454292 | Hair morphology |
| rs4875598 | Attention deficit hyperactivity disorder symptoms (interaction) |
| rs2738113 | Endometriosis |
| rs332034 | Conduct disorder (interaction) |
| rs9987289 | HDL cholesterol,HDL Cholesterol - Triglycerides (HDLC-TG),C-reactive protein,Cholesterol, total,Metabolic syndrome (bivariate traits),LDL cholesterol |
| rs2126259 | HDL cholesterol,LDL cholesterol |
| rs11775334 | Hypertension |
| rs11782819 | Alzheimer's disease |
| rs7819412 | Triglycerides |

| | |
|---|---|
| rs7824557 | Retinal vascular caliber |
| rs2251301 | Response to antipsychotic therapy (extrapyramidal side effects) |
| rs2002030 | Cognitive performance |
| rs7812879 | Systemic lupus erythematosus |
| rs2736340 | Systemic lupus erythematosus,Rheumatoid arthritis,Kawasaki disease |
| rs13277113 | Systemic lupus erythematosus |
| rs2618476 | Systemic lupus erythematosus |
| rs2645424 | Non-alcoholic fatty liver disease histology (lobular),Non-alcoholic fatty liver disease histology (other) |
| rs4831837 | Morbidity-free survival |
| rs2720508 | Conduct disorder (case status) |
| rs1495741 | Triglycerides,Cholesterol, total,Bladder cancer |
| rs7816032 | Hyperactive-impulsive symptoms |
| rs17581368 | Entorhinal cortical thickness |
| rs7843479 | Mean corpuscular volume |
| rs2279590 | Alzheimer's disease |
| rs11136000 | Alzheimer's disease |
| rs4732812 | Suicidal ideation |
| rs10091038 | Response to statin therapy |
| rs2978263 | Cognitive performance |
| rs2553268 | Exercise treadmill test traits |
| rs16879552 | Hirschsprung's disease |
| rs6468442 | Menopause (age at onset) |
| rs2722425 | Fasting plasma glucose |
| rs4737009 | Glycated hemoglobin levels |
| rs6474412 | Smoking behavior |
| rs310558 | Pulmonary function |
| rs7821565 | Tuberculosis |
| rs9298506 | Intracranial aneurysm |
| rs7833986 | Height |
| rs13273123 | Height |
| rs9650315 | Height |
| rs7815788 | Height |
| rs954295 | Low-density lipoprotein cholesterol |
| rs1992045 | Bipolar disorder and schizophrenia |
| rs3110127 | RR interval (heart rate) |
| rs903027 | MRI atrophy measures |
| rs2218488 | Sleepiness |
| rs1963982 | Blood pressure |
| rs2116078 | Multiple sclerosis (age of onset) |
| rs10504543 | Echocardiographic traits |
| rs12679254 | Inattentive symptoms |
| rs6472866 | Protein quantitative trait loci |
| rs2922763 | Body mass index |
| rs7821178 | Menarche (age at onset) |
| rs7846385 | Height |
| rs2717536 | Low-density lipoprotein cholesterol |

| | |
|---|---|
| rs6473383 | Heart failure |
| rs1375785 | Information processing speed |
| rs4397449 | Cognitive performance |
| rs160441 | Tuberculosis |
| rs42490 | Leprosy |
| rs40457 | Leprosy |
| rs896854 | Type 2 diabetes |
| rs4392868 | Radiation response |
| rs7000734 | Radiation response |
| rs1835740 | Migraine |
| rs1371867 | Atrioventricular conduction |
| rs3108919 | AIDS |
| rs931812 | Attention deficit hyperactivity disorder and conduct disorder |
| rs1264202 | Reasoning |
| rs517811 | Cognitive performance |
| rs977396 | Response to antipsychotic treatment |
| rs2935776 | T-tau |
| rs7832552 | Body mass (lean) |
| rs4876662 | Aortic root size |
| rs16892766 | Colorectal cancer |
| rs11989122 | Height |
| rs2062375 | Osteoporosis |
| rs2062377 | Bone mineral density,Bone mineral density (spine) |
| rs11995824 | Bone mineral density (hip) |
| rs6469804 | Bone mineral density (spine) |
| rs6993813 | Bone mineral density (hip) |
| rs1364705 | Hippocampal atrophy |
| rs7844723 | Hemostatic factors and hematological phenotypes |
| rs907121 | Weight |
| rs10088262 | Pancreatic cancer |
| rs2954021 | Liver enzyme levels (alkaline phosphatase),Liver enzyme levels (alanine transaminase),LDL cholesterol |
| rs17321515 | Triglycerides |
| rs2954029 | HDL cholesterol,Triglycerides,Cholesterol, total,LDL cholesterol |
| rs2954038 | Response to statin therapy |
| rs1456315 | Prostate cancer |
| rs2456449 | Chronic lymphocytic leukemia |
| rs445114 | Prostate cancer |
| rs13281615 | Breast cancer |
| rs1562430 | Breast cancer |
| rs10505477 | Colorectal cancer |
| rs6983267 | Colorectal cancer,Prostate cancer |
| rs7014346 | Colorectal cancer |
| rs1447295 | Prostate cancer |
| rs4242382 | Prostate cancer |
| rs4242384 | Prostate cancer |
| rs7837688 | Prostate cancer |

| | |
|---|---|
| rs9642880 | Urinary bladder cancer,Bladder cancer |
| rs2648875 | End-stage renal disease |
| rs2608053 | Hodgkin's lymphoma |
| rs10492294 | Immunoglobulin A |
| rs9792269 | Celiac disease |
| rs987525 | Cleft lip,Orofacial clefts |
| rs4295627 | Glioma |
| rs6470764 | Height |
| rs263238 | Total ventricular volume |
| rs2014357 | Common traits (Other) |
| rs4243849 | Non-alcoholic fatty liver disease histology (other) |
| rs12680546 | Amyotrophic lateral sclerosis |
| rs9657451 | Cognitive performance |
| rs2705293 | Neuroticism |
| rs7386474 | Bipolar disorder and schizophrenia |
| rs13263959 | Hair color |
| rs755383 | Testicular cancer,Testicular germ cell cancer,Testicular germ cell tumor |
| rs385893 | Platelet counts,Hematological parameters |
| rs10758658 | Mean corpuscular hemoglobin,Mean corpuscular volume |
| rs10758669 | Crohn's disease,Ulcerative colitis,Inflammatory bowel disease |
| rs10974944 | Myeloproliferative neoplasms |
| rs10975003 | Ulcerative colitis |
| rs842304 | HIV-1 susceptibility |
| rs1535480 | Common traits (Other) |
| rs7044355 | Platelet aggregation |
| rs10815798 | Attention deficit hyperactivity disorder and conduct disorder |
| rs17584499 | Type 2 diabetes |
| rs2475335 | Partial epilepsies |
| rs1325154 | Attention deficit hyperactivity disorder (time to onset) |
| rs1360517 | AIDS |
| rs1556032 | AIDS |
| rs10961780 | Height |
| rs471364 | HDL cholesterol |
| rs643531 | HDL cholesterol |
| rs1927702 | Body mass index |
| rs2153271 | Freckling |
| rs3814113 | Ovarian cancer in BRCA1 mutation carriers,Ovarian cancer |
| rs10810865 | Cognitive performance |
| rs1755289 | Multiple sclerosis |
| rs4636294 | Cutaneous nevi |
| rs7023329 | Melanoma |
| rs4977756 | Glioma,Glaucoma |
| rs1333040 | Intracranial aneurysm |
| rs4977574 | Myocardial infarction (early onset),Coronary heart disease |
| rs2383207 | Abdominal aortic aneurysm |
| rs10757278 | Myocardial infarction |
| rs1333049 | Coronary artery calcification,Coronary heart disease |

| | |
|---|---|
| rs2383208 | Type 2 diabetes |
| rs10965250 | Type 2 diabetes |
| rs10811661 | Fasting glucose-related traits (interaction with BMI),Type 2 diabetes |
| rs961831 | Personality dimensions |
| rs613391 | Quantitative traits |
| rs10811771 | Response to antipsychotic therapy (extrapyramidal side effects) |
| rs17774966 | Entorhinal cortical thickness |
| rs1889899 | Smoking behavior |
| rs7045881 | Schizophrenia |
| rs7046653 | Response to TNF antagonist treatment |
| rs10812610 | Heart failure |
| rs2814707 | Amyotrophic lateral sclerosis |
| rs3849942 | Amyotrophic lateral sclerosis |
| rs12555345 | Total ventricular volume |
| rs7029145 | Immunoglobulin A |
| rs10968576 | Body mass index,Obesity |
| rs12336160 | Erectile dysfunction and prostate cancer treatment |
| rs7871764 | Height |
| rs2812378 | Rheumatoid arthritis |
| rs951005 | Rheumatoid arthritis |
| rs10972341 | Weight |
| rs7873102 | Brain structure |
| rs4744712 | Chronic kidney disease |
| rs11143230 | Suicidal ideation |
| rs11144134 | Magnesium levels |
| rs10781380 | Hippocampal atrophy |
| rs2769967 | Inattentive symptoms |
| rs2151145 | Menopause (age at onset) |
| rs12554086 | RR interval (heart rate) |
| rs10867752 | Hippocampal atrophy |
| rs668853 | Ulcerative colitis |
| rs1998303 | Select biomarker traits |
| rs2841498 | Partial epilepsies |
| rs2814828 | Height |
| rs2778031 | Height |
| rs1831521 | Cognitive test performance |
| rs773506 | Type 2 diabetes nephropathy |
| rs9969804 | Height |
| rs3802458 | Erectile dysfunction and prostate cancer treatment |
| rs965513 | Thyroid cancer (Papillary, radiation-related),Thyroid cancer |
| rs755109 | Quantitative traits |
| rs10760706 | Alopecia areata |
| rs10989661 | Smoking behavior |
| rs4149268 | HDL cholesterol |
| rs3890182 | HDL cholesterol |
| rs3905000 | HDL cholesterol,MRI atrophy measures |
| rs1883025 | HDL cholesterol,Metabolic syndrome,Cholesterol, total |

| | |
|---|---|
| rs7861820 | Menarche and menopause (age at onset) |
| rs2090409 | Menarche (age at onset),Pubertal anthropometrics |
| rs4743034 | Height |
| rs7042864 | Tonometry |
| rs865686 | Breast cancer |
| rs7042161 | Bipolar disorder |
| rs10980926 | Menarche (age at onset),Pubertal anthropometrics |
| rs946053 | Height |
| rs4246905 | Ulcerative colitis,Inflammatory bowel disease |
| rs6478108 | Leprosy |
| rs4263839 | Crohn's disease |
| rs6478109 | Inflammatory bowel disease |
| rs876347 | Response to antipsychotic therapy (extrapyramidal side effects) |
| rs1572299 | Schizophrenia |
| rs11789399 | Bipolar disorder and schizophrenia |
| rs4837628 | Parkinson's disease |
| rs881375 | Rheumatoid arthritis |
| rs3761847 | Rheumatoid arthritis |
| rs2479106 | Polycystic ovary syndrome |
| rs2807580 | Cognitive performance |
| rs888219 | Response to antipsychotic treatment |
| rs4130590 | Bipolar disorder |
| rs7865146 | Metabolic syndrome |
| rs2502731 | Attention deficit hyperactivity disorder |
| rs7866070 | Common traits (Other) |
| rs7466269 | Height |
| rs11243897 | Attention deficit hyperactivity disorder |
| rs1076160 | Psoriasis |
| rs2905072 | Bipolar disorder |
| rs657152 | Liver enzyme levels,Obesity-related traits,Phytosterol levels,Thyroid hormone levels |
| rs514659 | Coronary heart disease |
| rs4962153 | Liver enzyme levels |
| rs7044529 | Central corneal thickness,Corneal structure |
| rs1537415 | Periodontitis |
| rs7849585 | Height |
| rs12338076 | Height |
| rs10794720 | Chronic kidney disease |
| rs11250464 | Radiation response |
| rs2999399 | Emphysema-related traits |
| rs6560749 | Quantitative traits |
| rs729397 | Response to statin therapy |
| rs2764980 | Attention deficit hyperactivity disorder and conduct disorder |
| rs6601764 | Crohn's disease |
| rs10458787 | Body mass index |
| rs1391511 | Neonatal lupus |
| rs2380205 | Breast cancer |

| | |
|---|---|
| rs706779 | Vitiligo |
| rs706778 | Rheumatoid arthritis |
| rs2104286 | Multiple sclerosis |
| rs3118470 | Alopecia areata,Multiple sclerosis |
| rs12251307 | Type 1 diabetes |
| rs947474 | Type 1 diabetes |
| rs4750316 | Rheumatoid arthritis |
| rs501764 | Hodgkin's lymphoma |
| rs10795668 | Colorectal cancer |
| rs10906115 | Type 2 diabetes |
| rs12779790 | Type 2 diabetes |
| rs525455 | Platelet aggregation |
| rs1561570 | Paget's disease |
| rs1541010 | RR interval (heart rate) |
| rs7077361 | Parkinson's disease |
| rs10508517 | Quantitative traits |
| rs11254363 | Folate pathway vitamin levels |
| rs6602175 | MRI atrophy measures |
| rs11014166 | Diastolic blood pressure,Hypertension,Systolic blood pressure |
| rs7069923 | Quantitative traits |
| rs7076247 | Protein quantitative trait loci |
| rs1326986 | Ankylosing spondylitis |
| rs16920624 | Response to antidepressants |
| rs2359536 | Peripheral artery disease |
| rs11013962 | Common traits (Other) |
| rs2484873 | Cognitive performance |
| rs927675 | MRI atrophy measures |
| rs1612122 | Major depressive disorder (broad) |
| rs1927457 | Normalized brain volume |
| rs2986971 | Non-alcoholic fatty liver disease histology (lobular) |
| rs2994684 | Response to antipsychotic treatment |
| rs11009175 | Depression (quantitative trait) |
| rs7905537 | Emphysema-related traits |
| rs17582416 | Crohn's disease |
| rs12261843 | Ulcerative colitis |
| rs1779876 | Protein quantitative trait loci |
| rs1200821 | Hemostatic factors and hematological phenotypes |
| rs2742234 | Hirschsprung's disease |
| rs501120 | Coronary heart disease |
| rs1746048 | Myocardial infarction (early onset),Coronary heart disease |
| rs11239177 | Bipolar disorder and schizophrenia |
| rs1480597 | Parkinson's disease |
| rs7911712 | Emphysema-related traits |
| rs11239550 | Mean corpuscular volume |
| rs4838605 | Diabetic retinopathy |
| rs10776612 | Conduct disorder (case status) |
| rs3849150 | Subclinical atherosclerosis traits (other) |

| | |
|---|---|
| rs1913517 | Systemic lupus erythematosus |
| rs4838508 | HIV-1 control |
| rs1733724 | Electrocardiographic traits,Ventricular conduction |
| rs583012 | Select biomarker traits |
| rs10740609 | Weight |
| rs4462262 | Diabetic retinopathy |
| rs1819658 | Crohn's disease |
| rs12356193 | Uric acid levels |
| rs10761482 | Schizophrenia |
| rs10994336 | Bipolar disorder |
| rs10994338 | Bipolar disorder and major depressive disorder (combined) |
| rs1530440 | Diastolic blood pressure |
| rs10821936 | Acute lymphoblastic leukemia (childhood) |
| rs7089424 | Acute lymphoblastic leukemia (childhood) |
| rs10995271 | Crohn's disease |
| rs10761659 | Crohn's disease,Inflammatory bowel disease |
| rs224136 | Crohn's disease |
| rs12355784 | Liver enzyme levels |
| rs2393967 | Mean platelet volume |
| rs2893923 | Platelet aggregation |
| rs10761779 | Liver enzyme levels |
| rs16922827 | Low-density lipoprotein cholesterol |
| rs10762058 | Asthma (toluene diisocyanate-induced) |
| rs4142041 | Nicotine dependence |
| rs6480314 | Optic disc size (disc) |
| rs1900004 | Optic disc parameters,Vertical cup-disc ratio |
| rs3858145 | Optic disc size (cup),Optic disc size (disc) |
| rs12571093 | Optic disc size (disc) |
| rs7072268 | Glycated hemoglobin levels |
| rs7077164 | Non-alcoholic fatty liver disease histology (lobular) |
| rs1227756 | Non-alcoholic fatty liver disease histology (lobular) |
| rs10999409 | Obesity (extreme) |
| rs1816002 | Weight |
| rs16928529 | Attention deficit hyperactivity disorder |
| rs1245541 | Insulin-like growth factors |
| rs2395528 | Conduct disorder (interaction) |
| rs704010 | Breast cancer |
| rs703965 | Bipolar disorder |
| rs11593576 | Vitiligo |
| rs1250540 | Multiple sclerosis |
| rs1250552 | Celiac disease |
| rs1250550 | Crohn's disease,Inflammatory bowel disease (early onset),Multiple sclerosis |
| rs1484170 | Cardiac hypertrophy |
| rs588517 | Carotid atherosclerosis in HIV infection |
| rs4933824 | Response to iloperidone treatment (QT prolongation) |
| rs2224865 | Eosinophilic esophagitis (pediatric) |
| rs9664222 | Longevity |

| | |
|---|---|
| rs10887741 | Exercise (leisure time) |
| rs10509540 | Type 1 diabetes |
| rs1926203 | Lung cancer |
| rs1937332 | Atrioventricular conduction |
| rs1329650 | Smoking behavior |
| rs1111875 | Type 2 diabetes |
| rs5015480 | Type 2 diabetes |
| rs3781264 | Esophageal cancer and gastric cancer |
| rs4086116 | Warfarin maintenance dose |
| rs10786284 | Attention deficit hyperactivity disorder |
| rs531676 | Metabolic syndrome |
| rs11597390 | Liver enzyme levels |
| rs4509693 | Alzheimer's disease |
| rs7071247 | Platelet aggregation |
| rs3814219 | Endothelial function traits |
| rs1320448 | Cardiac hypertrophy |
| rs515910 | Attention deficit hyperactivity disorder |
| rs869244 | Platelet aggregation |
| rs10885122 | Fasting glucose-related traits (interaction with BMI),Fasting glucose-related traits |
| rs7901695 | Type 2 diabetes,Coronary heart disease |
| rs4506565 | Type 2 diabetes,Fasting glucose-related traits |
| rs7903146 | Metabolic syndrome,Proinsulin levels,Fasting insulin-related traits (interaction with BMI),Fasting glucose-related traits (interaction with BMI),Glycated hemoglobin levels,Type 2 diabetes,Type 2 diabetes and other traits |
| rs12243326 | Two-hour glucose challenge |
| rs4751674 | Cognitive performance |
| rs10490919 | Information processing speed |
| rs740363 | Heart failure |
| rs2184898 | Conduct disorder (case status) |
| rs10788160 | Prostate-specific antigen levels |
| rs2981579 | Breast cancer |
| rs2981575 | Breast cancer |
| rs1219648 | Breast cancer |
| rs2981582 | Breast cancer |
| rs10510102 | Breast cancer |
| rs6585827 | Height |
| rs3793917 | Age-related macular degeneration |
| rs11200638 | Age-related macular degeneration (wet) |
| rs4962416 | Prostate cancer |
| rs4363506 | Amyotrophic lateral sclerosis |
| rs9804317 | Cognitive performance |
| rs4751178 | Speech perception in dyslexia |
| rs4751185 | HIV-1 susceptibility |
| rs4963128 | Systemic lupus erythematosus |
| rs3817198 | Breast cancer |
| rs909116 | Breast cancer |

| | |
|---|---|
| rs1004446 | Type 1 diabetes,Type 1 diabetes autoantibodies |
| rs7111341 | Type 1 diabetes |
| rs7127900 | Prostate cancer |
| rs2074238 | QT interval |
| rs12296050 | Electrocardiographic traits,QT interval |
| rs12576239 | QT interval |
| rs179429 | Platelet aggregation |
| rs2237878 | Protein quantitative trait loci |
| rs2237895 | Type 2 diabetes |
| rs16928809 | Bilirubin levels |
| rs3847646 | Calcium levels |
| rs11036238 | Malaria |
| rs2071348 | Beta thalassemia/hemoglobin E disease |
| rs4910742 | Fetal hemoglobin levels,Inflammatory biomarkers |
| rs110419 | Neuroblastoma |
| rs10769908 | Body mass index |
| rs6484218 | Schizophrenia, bipolar disorder and depression (combined) |
| rs7940646 | Platelet aggregation |
| rs2018368 | Bipolar disorder and schizophrenia |
| rs11042937 | Low-density lipoprotein cholesterol |
| rs7120489 | Mortality among heart failure patients |
| rs900145 | Menarche (age at onset) |
| rs9630182 | Bone mineral density |
| rs1993116 | Vitamin D levels |
| rs10741657 | Vitamin D insufficiency |
| rs2060793 | Vitamin D levels |
| rs7117858 | Bone mineral density (hip) |
| rs297325 | Obesity and osteoporosis |
| rs381815 | Blood pressure,Systolic blood pressure |
| rs11024074 | Diastolic blood pressure |
| rs4638289 | Amyloid A Levels |
| rs2896526 | Amyloid A Levels |
| rs874426 | Attention deficit hyperactivity disorder (time to onset) |
| rs7128311 | Response to treatment for acute lymphoblastic leukemia |
| rs1793004 | Crohn's disease |
| rs4561213 | Magnesium levels |
| rs12794435 | Ovarian cancer |
| rs7481311 | Weight,Body mass index |
| rs925946 | Weight,Body mass index |
| rs6265 | Weight,Smoking behavior,Body mass index |
| rs10767664 | Body mass index |
| rs10767971 | Parkinson's disease (age of onset) |
| rs5028798 | Volumetric brain MRI |
| rs286913 | Response to antipsychotic treatment |
| rs12808199 | Osteoporosis |
| rs4611189 | Reasoning |
| rs10501293 | Cognitive performance |

| | |
|---|---|
| rs11605924 | Fasting glucose-related traits (interaction with BMI),Fasting glucose-related traits |
| rs16938437 | Menarche (age at onset) |
| rs7932354 | Bone mineral density (hip),Bone mineral density |
| rs1007738 | Bone mineral density (hip) |
| rs7120118 | HDL cholesterol |
| rs3817334 | Body mass index |
| rs10838738 | Body mass index |
| rs7395662 | HDL cholesterol |
| rs1397048 | Hemostatic factors and hematological phenotypes |
| rs526934 | Folate pathway vitamin levels |
| rs7930940 | Low-density lipoprotein cholesterol |
| rs17824933 | Multiple sclerosis |
| rs4939490 | Multiple sclerosis |
| rs102275 | Crohn's disease,Metabolic syndrome,Metabolite levels,Palmitoleic acid (16:1n-7) plasma levels,Phospholipid levels (plasma),Oleic acid (18:1n-9) plasma levels,Stearic acid (18:0) plasma levels |
| rs174547 | Comprehensive strength and appendicular lean mass,HDL cholesterol,Metabolite levels,Metabolic traits,Phospholipid levels (plasma),Triglycerides,Resting heart rate,Lipid metabolism phenotypes |
| rs174548 | HDL cholesterol,Metabolite levels,Hematology traits,Triglycerides |
| rs174550 | Fasting glucose-related traits (interaction with BMI),Phospholipid levels (plasma),Fasting glucose-related traits |
| rs174570 | HDL cholesterol,Cholesterol, total,LDL cholesterol |
| rs1535 | Metabolic syndrome,Phospholipid levels (plasma),Response to statin therapy |
| rs174583 | Response to statin therapy |
| rs1000778 | Sphingolipid levels |
| rs2244621 | Low-density lipoprotein cholesterol |
| rs694739 | Crohn's disease,Alopecia areata |
| rs17300741 | Uric acid levels |
| rs2078267 | Urate levels |
| rs505802 | Uric acid levels |
| rs17146964 | Vertical cup-disc ratio |
| rs4014195 | Chronic kidney disease |
| rs2242663 | Bipolar disorder |
| rs599083 | Bone mineral density (spine) |
| rs11228565 | Prostate cancer |
| rs7931342 | Prostate cancer |
| rs10896449 | Prostate cancer |
| rs7130881 | Prostate cancer |
| rs7105934 | Renal cell carcinoma |
| rs614367 | Breast cancer |
| rs12785878 | Vitamin D insufficiency |
| rs634552 | Height |
| rs2155219 | Ulcerative colitis,IgE grass sensitization,Allergic rhinitis,Inflammatory bowel disease |
| rs2373115 | Alzheimer's disease (late onset) |

| | |
|---|---|
| rs10899489 | Menarche (age at onset) |
| rs530965 | Cognitive test performance |
| rs1458095 | Body mass index |
| rs10501570 | Parkinson's disease |
| rs1452928 | Hip geometry |
| rs10898392 | Height |
| rs3851179 | Alzheimer's disease |
| rs6592284 | Cognitive performance |
| rs1386330 | Multiple sclerosis (age of onset) |
| rs10831496 | Tanning |
| rs1488902 | Amyotrophic lateral sclerosis |
| rs1528753 | Aging traits |
| rs1350445 | Subclinical atherosclerosis traits (other) |
| rs1387153 | Fasting plasma glucose,Glycated hemoglobin levels,Type 2 diabetes,Metabolic syndrome (bivariate traits) |
| rs2166706 | Fasting plasma glucose |
| rs10830963 | Metabolite levels,Obesity-related traits,Fasting glucose-related traits (interaction with BMI),Fasting plasma glucose,Fasting glucose-related traits |
| rs1447352 | Metabolic traits |
| rs10831284 | Attention deficit hyperactivity disorder and conduct disorder |
| rs1939875 | Eosinophilic esophagitis (pediatric) |
| rs7115578 | Response to treatment for acute lymphoblastic leukemia |
| rs2405657 | Amyotrophic lateral sclerosis |
| rs2509843 | Bipolar disorder and schizophrenia |
| rs4237591 | Non-alcoholic fatty liver disease histology (lobular) |
| rs952700 | Volumetric brain MRI |
| rs2852894 | MRI atrophy measures |
| rs495366 | Matrix metalloproteinase levels |
| rs716274 | Small-cell lung cancer |
| rs10895959 | Inattentive symptoms |
| rs10431058 | Common traits (Other) |
| rs11212617 | Response to metformin |
| rs746463 | Exercise treadmill test traits |
| rs3802842 | Colorectal cancer |
| rs2115763 | Interleukin-18 levels |
| rs7105881 | Response to antipsychotic treatment |
| rs17116334 | Conduct disorder (interaction) |
| rs2847476 | Volumetric brain MRI |
| rs678170 | Ulcerative colitis |
| rs1712790 | Urinary albumin excretion |
| rs638882 | Hip geometry |
| rs490592 | Brain structure |
| rs1240773 | HIV-1 susceptibility |
| rs4938303 | Triglycerides |
| rs7350481 | Triglycerides,Hematological and biochemical traits |
| rs12272004 | Carotenoid and tocopherol levels,Triglycerides,Cholesterol, total,LDL cholesterol |

| | |
|---|---|
| rs1558861 | Triglycerides,LDL cholesterol |
| rs12280753 | Cardiovascular disease risk factors |
| rs964184 | HDL cholesterol,Vitamin E levels,Metabolic syndrome,Metabolite levels,Response to Vitamin E supplementation,Phospholipid levels (plasma),Triglycerides,Cholesterol, total,LDL cholesterol,Coronary heart disease,Lipoprotein-associated phospholipase A2 activity and mass,Hypertriglyceridemia |
| rs12286037 | Triglycerides,Metabolic syndrome (bivariate traits),Lipoprotein-associated phospholipase A2 activity and mass |
| rs6589566 | Triglycerides,LDL cholesterol |
| rs7396835 | Quantitative traits |
| rs10047462 | Iron status biomarkers |
| rs2075292 | Triglycerides |
| rs7112513 | Protein quantitative trait loci |
| rs236918 | Iron status biomarkers |
| rs17122021 | Pain |
| rs4639966 | Systemic lupus erythematosus |
| rs6589964 | Menarche (age at onset) |
| rs735665 | Follicular lymphoma,Chronic lymphocytic leukemia |
| rs544368 | Bipolar disorder |
| rs563519 | Heart failure |
| rs1695739 | Low-density lipoprotein cholesterol |
| rs1557488 | Attention deficit hyperactivity disorder and conduct disorder |
| rs1939992 | Protein quantitative trait loci |
| rs620875 | Response to antipsychotic treatment |
| rs6590322 | Hippocampal atrophy |
| rs11221332 | Celiac disease |
| rs1550976 | Asperger disorder |
| rs4397868 | Menopause (age at onset) |
| rs1031381 | Cognitive test performance |
| rs10774021 | Chronic kidney disease |
| rs12425791 | Stroke |
| rs10848704 | Quantitative traits |
| rs10848911 | Male infertility |
| rs4238010 | Major depressive disorder |
| rs11611647 | Red blood cell count |
| rs10849033 | Acute lymphoblastic leukemia (childhood) |
| rs12579350 | Panic disorder |
| rs1800693 | Multiple sclerosis,Primary biliary cirrhosis |
| rs6487679 | Non-alcoholic fatty liver disease histology (AST) |
| rs11052552 | Type 1 diabetes |
| rs4763879 | Type 1 diabetes |
| rs2187642 | Height |
| rs2856321 | Height |
| rs2900333 | Testicular germ cell cancer,Testicular germ cell tumor |
| rs1348582 | QT interval |
| rs10770705 | Height |

| | |
|---|---|
| rs2117032 | Bilirubin levels |
| rs10743430 | Entorhinal cortical thickness |
| rs2216228 | Non-alcoholic fatty liver disease histology (lobular) |
| rs7979575 | Response to statin therapy |
| rs1464500 | Response to antipsychotic treatment |
| rs17287293 | Heart rate,Resting heart rate |
| rs11047543 | PR interval |
| rs718314 | Waist-hip ratio,Renal cell carcinoma |
| rs2306677 | Amyotrophic lateral sclerosis |
| rs522958 | Hyperactive-impulsive symptoms,Attention deficit hyperactivity disorder |
| rs2638953 | Height |
| rs2046383 | Heart failure |
| rs1463605 | Aging traits |
| rs10844154 | Weight,Emphysema-related traits |
| rs708224 | Pancreatic cancer |
| rs261902 | Normalized brain volume |
| rs9300212 | Cognitive test performance |
| rs6582630 | Drug-induced liver injury (flucloxacillin) |
| rs1994090 | Parkinson's disease |
| rs11175593 | Crohn's disease |
| rs1491942 | Parkinson's disease |
| rs11564258 | Crohn's disease,Inflammatory bowel disease |
| rs1880887 | Protein quantitative trait loci |
| rs1458175 | Multiple sclerosis |
| rs871392 | Biochemical measures |
| rs2731006 | Panic disorder |
| rs1520832 | Heart failure |
| rs1373549 | Hippocampal atrophy |
| rs7138803 | Weight,Waist circumference,Body mass index,Obesity |
| rs11169552 | Colorectal cancer |
| rs2016266 | Bone mineral density,Bone mineral density (spine) |
| rs10876432 | Bone mineral density (spine) |
| rs11170631 | Height |
| rs7969151 | Tanning |
| rs2120991 | Biliary atresia |
| rs1443512 | Waist-hip ratio |
| rs10506328 | Mean platelet volume |
| rs1153188 | Type 2 diabetes |
| rs11171739 | Type 1 diabetes |
| rs2292239 | Type 1 diabetes,Type 1 diabetes autoantibodies |
| rs2958154 | Age-related macular degeneration |
| rs12367822 | Platelet aggregation |
| rs167769 | Eosinophilic esophagitis (pediatric) |
| rs1106766 | Urate levels |
| rs1678542 | Rheumatoid arthritis |
| rs10506410 | Cardiac hypertrophy |
| rs7302017 | Waist circumference |

| | |
|---|---|
| rs10506458 | Hemostatic factors and hematological phenotypes |
| rs10506525 | Primary tooth development (time to first tooth eruption),Primary tooth development (number of teeth) |
| rs2358944 | Type 2 diabetes nephropathy |
| rs10784496 | AB1-42 |
| rs1531343 | Type 2 diabetes |
| rs4026608 | Aortic root size |
| rs7134599 | Ulcerative colitis,Inflammatory bowel disease |
| rs1558744 | Ulcerative colitis |
| rs2870946 | Ulcerative colitis |
| rs12313946 | White blood cell count |
| rs9943849 | Major depressive disorder |
| rs317689 | Response to diuretic therapy |
| rs315135 | Response to diuretic therapy |
| rs10748128 | Height |
| rs11177669 | Height |
| rs1495377 | Creutzfeldt-Jakob disease (variant),Type 2 diabetes |
| rs4760790 | Type 2 diabetes |
| rs7961581 | Type 2 diabetes |
| rs12831974 | Rheumatoid arthritis |
| rs7963521 | Chemerin levels |
| rs10506701 | Bone mineral density |
| rs1402279 | Smoking behavior |
| rs300489 | Self-rated health |
| rs7297018 | Attention deficit hyperactivity disorder and conduct disorder |
| rs3782181 | Testicular germ cell cancer |
| rs4474514 | Testicular cancer |
| rs2681472 | Diastolic blood pressure,Hypertension |
| rs2681492 | Systolic blood pressure |
| rs17249754 | Biomedical quantitative traits,Blood pressure |
| rs10858945 | Optic disc size (cup) |
| rs10777332 | Biochemical measures |
| rs17019682 | Heart failure |
| rs1836127 | Non-alcoholic fatty liver disease histology (other) |
| rs11108495 | Weight |
| rs10507130 | Coronary artery calcification |
| rs7971536 | Height |
| rs35767 | Fasting glucose-related traits,Fasting insulin-related traits |
| rs10745954 | C-reactive protein |
| rs10778213 | C-reactive protein |
| rs4964805 | Attention deficit hyperactivity disorder |
| rs4964469 | Parkinson's disease |
| rs9943753 | HDL cholesterol |
| rs2338104 | HDL cholesterol |
| rs11065611 | Protein quantitative trait loci |
| rs10849915 | Alcohol consumption |
| rs10774625 | Retinal vascular caliber |

| | |
|---|---|
| rs653178 | Celiac disease,Celiac disease and Rheumatoid arthritis,Urate levels,Diastolic blood pressure,Blood pressure,Chronic kidney disease |
| rs11065987 | Hemoglobin,Hematocrit,Tetralogy of Fallot,Cholesterol, total,LDL cholesterol |
| rs17696736 | Type 1 diabetes |
| rs11066301 | Hematological parameters |
| rs3825214 | Electrocardiographic traits |
| rs11067228 | Prostate-specific antigen levels |
| rs1896312 | PR interval |
| rs2384550 | Diastolic blood pressure,Blood pressure |
| rs10850409 | Ventricular conduction |
| rs2194980 | Metabolite levels |
| rs4767631 | Biochemical measures |
| rs10444502 | Biochemical measures |
| rs1997111 | T-tau |
| rs2650000 | Metabolic traits,LDL cholesterol |
| rs7953249 | N-glycan levels,Chronic obstructive pulmonary disease-related biomarkers |
| rs1183910 | C-reactive protein |
| rs7310409 | C-reactive protein,Liver enzyme levels (gamma-glutamyl transferase) |
| rs2259816 | C-reactive protein,Coronary heart disease |
| rs735396 | N-glycan levels |
| rs1169313 | Liver enzyme levels |
| rs7957197 | Type 2 diabetes |
| rs7961894 | Platelet counts,Mean platelet volume |
| rs12817488 | Parkinson's disease |
| rs1790100 | Multiple sclerosis |
| rs11830103 | Height |
| rs4765623 | Renal cell carcinoma |
| rs78640837 | Depression (quantitative trait) |
| rs7299940 | Panic disorder |
| rs885389 | RR interval (heart rate) |
| rs7326068 | Schizophrenia, bipolar disorder and depression (combined) |
| rs17369571 | Protein quantitative trait loci |
| rs7318731 | RR interval (heart rate) |
| rs1034200 | Central corneal thickness,Corneal structure |
| rs1572072 | Nasopharyngeal carcinoma |
| rs17079773 | Inattentive symptoms |
| rs9319321 | Asthma (toluene diisocyanate-induced) |
| rs10507380 | Electrocardiographic traits |
| rs4771122 | Body mass index |
| rs7336332 | Weight |
| rs17086609 | Cognitive performance |
| rs1305088 | Non-alcoholic fatty liver disease histology (other) |
| rs1161463 | Attention deficit hyperactivity disorder symptoms (interaction) |
| rs9314986 | Biliary atresia |
| rs690705 | Alzheimer's disease |
| rs1777672 | HIV-1 control |
| rs1590305 | Hip geometry |

| | |
|---|---|
| rs7993214 | Psoriasis |
| rs9548988 | Ulcerative colitis |
| rs10492681 | Select biomarker traits |
| rs941823 | Ulcerative colitis,Inflammatory bowel disease |
| rs6561030 | Height |
| rs1012053 | Bipolar disorder |
| rs9533090 | Bone mineral density,Bone mineral density (spine) |
| rs9594738 | Bone mineral density (hip),Bone mineral density |
| rs9594759 | Bone mineral density (spine) |
| rs2062305 | Crohn's disease |
| rs1021188 | Bone mineral density |
| rs1324015 | Cognitive performance |
| rs958546 | Atrial fibrillation |
| rs2478333 | QT interval |
| rs1575891 | Cardiac hypertrophy |
| rs1262778 | Bipolar disorder and major depressive disorder (combined) |
| rs1239947 | Height |
| rs3116602 | Height |
| rs3118914 | Height |
| rs10507577 | Select biomarker traits |
| rs9536591 | Stroke |
| rs1512651 | Warfarin maintenance dose |
| rs10492604 | Sleep duration |
| rs9317284 | Bone mineral density |
| rs1333026 | Body mass index |
| rs1585440 | Pancreatic cancer |
| rs2066219 | Diabetes related insulin traits |
| rs9572423 | Major depressive disorder |
| rs626277 | Chronic kidney disease |
| rs9600079 | Prostate cancer |
| rs1886449 | Pancreatic cancer |
| rs1886512 | Ventricular conduction |
| rs8000245 | Optic disc size (rim) |
| rs548097 | Heart failure |
| rs2073831 | Low-density lipoprotein cholesterol |
| rs9574199 | Total ventricular volume |
| rs17070284 | Cognitive performance |
| rs2039553 | Pancreatic cancer |
| rs9574565 | Orofacial clefts |
| rs1359790 | Type 2 diabetes |
| rs8002779 | Height |
| rs2352028 | Lung cancer |
| rs9523762 | Multiple sclerosis |
| rs7995215 | Attention deficit hyperactivity disorder |
| rs9302001 | Panic disorder |
| rs1926657 | Breast cancer |
| rs4318070 | Total ventricular volume |

| | |
|---|---|
| rs688872 | Brain structure |
| rs9584805 | Non-alcoholic fatty liver disease histology (lobular) |
| rs7992643 | Attention deficit hyperactivity disorder |
| rs2044117 | Bipolar disorder and schizophrenia |
| rs4771450 | Uric acid levels |
| rs4996815 | Bipolar disorder and schizophrenia |
| rs957788 | Anorexia nervosa |
| rs767210 | Cognitive performance |
| rs4773330 | Bilirubin levels |
| rs9555810 | Menarche (age at onset) |
| rs12147450 | Response to antipsychotic therapy (extrapyramidal side effects) |
| rs2239633 | Acute lymphoblastic leukemia (childhood) |
| rs452036 | Resting heart rate |
| rs223116 | Resting heart rate |
| rs12436436 | Bipolar disorder |
| rs1950500 | Height |
| rs854384 | MRI atrophy measures |
| rs1951082 | Attention deficit hyperactivity disorder and conduct disorder |
| rs2038256 | Multiple sclerosis--Brain Glutamate Levels |
| rs7142881 | Response to iloperidone treatment (QT prolongation) |
| rs2039485 | Brain lesion load |
| rs915071 | Bipolar disorder and schizophrenia |
| rs2383378 | Anorexia nervosa |
| rs12586317 | Psoriasis |
| rs8016947 | Psoriasis |
| rs944289 | Thyroid cancer |
| rs12883384 | Neuroticism |
| rs7159841 | Hemostatic factors and hematological phenotypes |
| rs7151223 | Cognitive performance |
| rs1584157 | Low-density lipoprotein cholesterol |
| rs1265879 | Cognitive performance |
| rs17122693 | Cognitive performance |
| rs8020441 | Cognitive performance |
| rs7153703 | Total ventricular volume |
| rs7140150 | Brain structure |
| rs11626056 | Hippocampal atrophy |
| rs730532 | Pulmonary function |
| rs12431733 | Parkinson's disease |
| rs4444235 | Colorectal cancer |
| rs4293296 | Working memory |
| rs10134944 | Bipolar disorder |
| rs808225 | Pulmonary function |
| rs4901869 | Panic disorder |
| rs2093210 | Height |
| rs10483727 | Optic disc size (rim),Vertical cup-disc ratio,Glaucoma (primary open-angle) |
| rs8005745 | Select biomarker traits |
| rs973968 | Iron status biomarkers |

| | |
|---|---|
| rs7158173 | Anthropometric traits |
| rs7159888 | N-glycan levels,IgG glycosylation |
| rs10483776 | N-glycan levels |
| rs8007846 | Multiple sclerosis--Brain Glutamate Levels |
| rs1956529 | Primary tooth development (number of teeth) |
| rs4902642 | Crohn's disease |
| rs1465788 | Type 1 diabetes |
| rs4899260 | Celiac disease |
| rs2268983 | Smoking behavior |
| rs36563 | Alcohol dependence |
| rs11848785 | Ventricular conduction |
| rs4903031 | C-reactive protein |
| rs862034 | Height |
| rs7155603 | Rheumatoid arthritis |
| rs935334 | Blood pressure |
| rs2121070 | Blood pressure |
| rs2360997 | Attention deficit hyperactivity disorder symptoms (interaction) |
| rs6574433 | Cognitive performance |
| rs11159647 | Alzheimer's disease |
| rs12100561 | Hepatocellular carcinoma |
| rs8017423 | Mortality among heart failure patients |
| rs7153027 | Height |
| rs8007661 | Height |
| rs1187614 | Response to antipsychotic treatment |
| rs8005962 | Tuberculosis |
| rs8015016 | QT interval |
| rs4900384 | Type 1 diabetes |
| rs10484128 | Hemostatic factors and hematological phenotypes |
| rs6575793 | Menarche (age at onset) |
| rs1884537 | Optic disc size (disc) |
| rs941576 | Type 1 diabetes |
| rs4906172 | Menopause (age at onset) |
| rs11622475 | Bipolar disorder |
| rs10129255 | Kawasaki disease |
| rs3867498 | Pulmonary function |
| rs17636733 | Cardiac hypertrophy |
| rs8043440 | Cognitive performance |
| rs17565841 | Parkinson's disease (age of onset) |
| rs916977 | Iris color |
| rs1667394 | Blond vs. brown hair color,Blue vs. brown eyes,Eye color,Blue vs. green eyes |
| rs8033165 | Black vs. blond hair color,Black vs. red hair color |
| rs1471225 | Inattentive symptoms |
| rs2125623 | Mortality among heart failure patients |
| rs4779584 | Colorectal cancer |
| rs634990 | Refractive error |
| rs4923705 | Attention deficit hyperactivity disorder |
| rs10520045 | Major depressive disorder |

| | |
|---|---|
| rs8041675 | Hyperactive-impulsive symptoms |
| rs11073328 | Low-density lipoprotein cholesterol |
| rs8035957 | Type 1 diabetes |
| rs12912251 | Bipolar disorder and major depressive disorder (combined),Bipolar disorder |
| rs12899449 | Bipolar disorder |
| rs2624265 | Metabolic traits |
| rs12907914 | Cardiac hypertrophy |
| rs2453533 | Chronic kidney disease |
| rs2467853 | Renal function and chronic kidney disease |
| rs12594515 | Waist circumference,Weight |
| rs11637235 | Protein quantitative trait loci |
| rs2305707 | Height |
| rs1897031 | Cognitive performance |
| rs8032158 | Keloid |
| rs7169431 | Chronic lymphocytic leukemia |
| rs2934442 | Bipolar disorder (age of onset and psychomotor symptoms) |
| rs1550576 | Hypertension |
| rs4775031 | Cognitive performance |
| rs4775041 | HDL cholesterol,Metabolite levels,Triglycerides |
| rs10468017 | Age-related macular degeneration,HDL cholesterol,Cardiovascular disease risk factors,Phospholipid levels (plasma),Metabolic syndrome (bivariate traits) |
| rs1532085 | HDL cholesterol,Metabolic syndrome,Metabolite levels,Triglycerides,Cholesterol, total,Red blood cell traits,Lipid metabolism phenotypes |
| rs493258 | Age-related macular degeneration |
| rs261334 | HDL cholesterol |
| rs3825776 | Amyotrophic lateral sclerosis |
| rs7179456 | Asperger disorder |
| rs340029 | C-reactive protein |
| rs11071559 | Asthma |
| rs12912233 | Depression (quantitative trait) |
| rs809736 | Response to citalopram treatment |
| rs17271305 | Two-hour glucose challenge |
| rs7172432 | Type 2 diabetes |
| rs1436955 | Type 2 diabetes |
| rs11071657 | Fasting glucose-related traits |
| rs11071720 | Mean platelet volume |
| rs6494537 | Mean corpuscular hemoglobin |
| rs17293632 | Crohn's disease,Inflammatory bowel disease |
| rs7359257 | Menarche (age at onset) |
| rs12593813 | Restless legs syndrome |
| rs41371354 | Attention deficit hyperactivity disorder |
| rs11072089 | Bipolar disorder and schizophrenia |
| rs17374222 | Rheumatoid arthritis |
| rs4776472 | Atrial fibrillation |
| rs7176508 | Chronic lymphocytic leukemia |
| rs893817 | Aortic root size |

| | |
|---|---|
| rs1378942 | Diastolic blood pressure,Blood pressure,Systolic blood pressure |
| rs6495122 | Coffee consumption,Caffeine consumption,Diastolic blood pressure |
| rs4886707 | Height |
| rs1394125 | Urate levels,Chronic kidney disease |
| rs1533665 | Personality dimensions |
| rs8034191 | Chronic obstructive pulmonary disease,Lung cancer |
| rs3825932 | Type 1 diabetes |
| rs3743200 | RR interval (heart rate) |
| rs11634397 | Type 2 diabetes |
| rs2278702 | Bipolar disorder |
| rs12324805 | Body mass index |
| rs783540 | Chronic lymphocytic leukemia |
| rs1568657 | Kawasaki disease |
| rs2562784 | Height |
| rs2554380 | Height |
| rs10906982 | Height |
| rs7183263 | Height |
| rs7176093 | Aging traits |
| rs8041863 | Height |
| rs4932217 | Height |
| rs12915189 | Information processing speed |
| rs2677744 | Attention deficit hyperactivity disorder |
| rs8042680 | Type 2 diabetes |
| rs886144 | Metabolite levels |
| rs3924426 | Response to iloperidone treatment (QT prolongation) |
| rs7495052 | Inattentive symptoms |
| rs1455782 | Pulmonary function |
| rs7175404 | Attention deficit hyperactivity disorder |
| rs2388436 | Hoarding |
| rs4984390 | Drug-induced liver injury (flucloxacillin) |
| rs7173947 | Hematological and biochemical traits |
| rs6496074 | Cognitive performance |
| rs2567426 | Information processing speed |
| rs2398162 | Hypertension |
| rs4533251 | Attention deficit hyperactivity disorder and conduct disorder |
| rs4965121 | Neuroticism |
| rs2871865 | Height |
| rs4533267 | Height |
| rs11855415 | Handedness in dyslexia |
| rs7189020 | Mean corpuscular volume |
| rs1122794 | Mean corpuscular hemoglobin,Red blood cell traits |
| rs763014 | Height |
| rs26868 | Height |
| rs886427 | Metabolic syndrome |
| rs2444217 | Body mass index |
| rs2601828 | Partial epilepsies |
| rs12921846 | Attention deficit hyperactivity disorder and conduct disorder |

| | |
|---|---|
| rs9924951 | Metabolite levels |
| rs1463132 | HIV-1 control |
| rs11074889 | Attention deficit hyperactivity disorder |
| rs4781011 | Ulcerative colitis |
| rs6498142 | Immunoglobulin A |
| rs11865121 | Multiple sclerosis |
| rs12708716 | Type 1 diabetes,Type 1 diabetes autoantibodies |
| rs2903692 | Type 1 diabetes |
| rs6498169 | Multiple sclerosis |
| rs416603 | Type 1 diabetes |
| rs8049607 | QT interval |
| rs7192086 | Schizophrenia |
| rs153091 | Response to antipsychotic treatment |
| rs2113334 | Metabolic syndrome |
| rs3136202 | Conduct disorder (symptom count) |
| rs1659127 | Menarche (age at onset),Height |
| rs12444979 | Body mass index |
| rs12444268 | Type 1 diabetes |
| rs4293393 | Chronic kidney disease and serum creatinine levels |
| rs13333226 | Hypertension |
| rs12917707 | Renal function and chronic kidney disease,Chronic kidney disease |
| rs151222 | Schizophrenia |
| rs433598 | Schizophrenia |
| rs8049603 | Multiple sclerosis |
| rs420259 | Bipolar disorder |
| rs11643520 | Brain structure |
| rs2203512 | Cognitive performance |
| rs8057551 | Bone mineral density |
| rs151181 | Crohn's disease |
| rs4788084 | Type 1 diabetes,Type 1 diabetes autoantibodies |
| rs8049439 | Educational attainment,Inflammatory bowel disease (early onset) |
| rs7359397 | Body mass index |
| rs4787483 | Anthropometric traits |
| rs10782001 | Psoriasis |
| rs10871454 | Warfarin maintenance dose |
| rs9923231 | Warfarin maintenance dose |
| rs9888739 | Systemic lupus erythematosus |
| rs11150610 | Systemic lupus erythematosus |
| rs11574637 | Systemic lupus erythematosus |
| rs9302752 | Leprosy |
| rs17221417 | Crohn's disease |
| rs2076756 | Crohn's disease,Inflammatory bowel disease |
| rs4131099 | Personality dimensions |
| rs1362756 | Optic disc parameters |
| rs3803662 | Breast cancer (male),Breast cancer |
| rs4784227 | Breast cancer |
| rs3112612 | Breast cancer |

| | |
|---|---|
| rs6499640 | Weight,Body mass index |
| rs1421085 | Obesity (early onset extreme),Dietary macronutrient intake,Obesity |
| rs1558902 | Waist circumference,Obesity (extreme),Body mass index,Obesity |
| rs1121980 | Obesity (early onset extreme),Body mass index |
| rs8050136 | Weight,Body mass in chronic obstructive pulmonary disease,Body mass index,Adiposity,Type 2 diabetes |
| rs9939609 | Menarche (age at onset),Biomedical quantitative traits,Body mass index,Type 2 diabetes |
| rs9941349 | Obesity (extreme) |
| rs9930506 | Obesity-related traits |
| rs11642841 | Type 2 diabetes |
| rs17291845 | Information processing speed |
| rs9989419 | HDL cholesterol |
| rs1800775 | HDL cholesterol,Triglycerides,Lipid metabolism phenotypes |
| rs1864163 | Age-related macular degeneration,HDL cholesterol,Lipid metabolism phenotypes |
| rs1532624 | HDL cholesterol,Cholesterol,Lipid metabolism phenotypes |
| rs7499892 | Biochemical measures,Lipid metabolism phenotypes |
| rs37062 | Electrocardiographic traits,QT interval |
| rs7188697 | QT interval |
| rs2639889 | Morbidity-free survival |
| rs1381102 | Attention deficit hyperactivity disorder and conduct disorder |
| rs255049 | HDL cholesterol |
| rs1728785 | Ulcerative colitis |
| rs6499188 | Ulcerative colitis |
| rs9929218 | Colorectal cancer |
| rs8047014 | Attention deficit hyperactivity disorder |
| rs1364063 | Menarche (age at onset) |
| rs7193343 | Atrial fibrillation |
| rs12446956 | Major depressive disorder |
| rs10871290 | Breast cancer |
| rs1948632 | Tuberculosis |
| rs13330107 | Inattentive symptoms |
| rs2059238 | Cardiac structure and function |
| rs2859631 | Radiation response |
| rs7498403 | Height |
| rs1424233 | Obesity |
| rs4889240 | Attention deficit hyperactivity disorder and conduct disorder |
| rs6564851 | Carotenoid and tocopherol levels |
| rs4889294 | Protein quantitative trait loci |
| rs4783227 | Response to antipsychotic treatment |
| rs11646213 | Hypertension |
| rs8055236 | Coronary heart disease |
| rs10514585 | Depression (quantitative trait) |
| rs6563943 | Height |
| rs3096277 | Blood pressure |
| rs10514604 | Attention deficit hyperactivity disorder |

| | |
|---|---|
| rs371915 | Eosinophilic esophagitis (pediatric) |
| rs2326458 | Height |
| rs305061 | Chronic lymphocytic leukemia |
| rs16940202 | Ulcerative colitis |
| rs10048146 | Bone mineral density (hip),Bone mineral density,Bone mineral density (spine) |
| rs4843747 | Menopause (age at onset) |
| rs12447690 | Central corneal thickness |
| rs837763 | mean corpuscular hemoglobin concentration |
| rs258322 | Melanoma,Black vs. red hair color |
| rs11648785 | Tanning |
| rs10852932 | Aortic root size |
| rs391300 | Type 2 diabetes |
| rs220470 | Attention deficit hyperactivity disorder |
| rs9303196 | Non-small cell lung cancer |
| rs16956936 | Type 1 diabetes |
| rs10521157 | Low-density lipoprotein cholesterol |
| rs6503319 | Malaria |
| rs10521232 | Tonometry |
| rs4792394 | Conduct disorder (symptom count) |
| rs3848445 | Protein quantitative trait loci |
| rs4273077 | Hematological and biochemical traits |
| rs4924935 | Pancreatic cancer |
| rs4795067 | Psoriasis |
| rs2138852 | Mean platelet volume |
| rs3760318 | Height |
| rs225190 | Pancreatic cancer |
| rs3091315 | Crohn's disease |
| rs2074518 | QT interval |
| rs8070473 | Depression (quantitative trait) |
| rs4796217 | Protein quantitative trait loci |
| rs712039 | Tuberculosis |
| rs7501939 | Prostate cancer |
| rs9303277 | Primary biliary cirrhosis |
| rs2872507 | Crohn's disease,Ulcerative colitis,Rheumatoid arthritis,Type 1 diabetes autoantibodies |
| rs8067378 | Ulcerative colitis |
| rs2290400 | Type 1 diabetes |
| rs7221109 | Type 1 diabetes |
| rs2315504 | Height |
| rs744166 | Crohn's disease,Multiple sclerosis |
| rs1513670 | Bone mineral density (hip) |
| rs228769 | Bone mineral density (hip),Bone mineral density (spine) |
| rs12946454 | Systolic blood pressure |
| rs4986172 | Height |
| rs2084881 | Ovarian cancer |
| rs9303542 | Ovarian cancer |
| rs6504340 | Primary tooth development (number of teeth) |

| | |
|---|---|
| rs2326017 | Cognitive performance,Body mass index (non-asthmatics) |
| rs9674544 | Primary tooth development (time to first tooth eruption),Primary tooth development (number of teeth) |
| rs16948048 | Diastolic blood pressure |
| rs1035050 | Bipolar disorder |
| rs2075555 | Breast cancer |
| rs9635759 | Menarche (age at onset) |
| rs8073783 | Conduct disorder (interaction) |
| rs12449568 | Height |
| rs4794665 | Height |
| rs792376 | Low-density lipoprotein cholesterol |
| rs9303401 | Cognitive test performance |
| rs8068952 | Vertical cup-disc ratio |
| rs9895661 | Renal function-related traits (sCR),Chronic kidney disease,Renal function-related traits (eGRFcrea) |
| rs8068318 | Creatinine levels |
| rs2079795 | Height |
| rs757608 | Height |
| rs7209435 | Height |
| rs2665838 | Height |
| rs8074751 | Attention deficit hyperactivity disorder |
| rs7209395 | Weight,β2-Glycoprotein I (β2-GPI) plasma levels |
| rs9912468 | Ventricular conduction |
| rs17645023 | Bipolar disorder and schizophrenia |
| rs8079702 | Primary tooth development (time to first tooth eruption),Primary tooth development (number of teeth) |
| rs6501384 | Eosinophilic esophagitis (pediatric) |
| rs1859962 | Prostate cancer |
| rs3816995 | Panic disorder |
| rs7219585 | Information processing speed |
| rs10512597 | Fibrinogen |
| rs6565681 | Moyamoya disease |
| rs7226677 | Bipolar disorder and major depressive disorder (combined),Bipolar disorder |
| rs8097348 | Exercise (leisure time) |
| rs2542151 | Type 1 diabetes,Crohn's disease,Type 1 diabetes autoantibodies |
| rs4331426 | Tuberculosis |
| rs11661542 | Intracranial aneurysm |
| rs11082304 | Platelet counts,Height,Smoking behavior |
| rs4800148 | Height |
| rs4369779 | Height |
| rs9635963 | Protein quantitative trait loci |
| rs7227401 | Osteoporosis |
| rs1840440 | Weight |
| rs11083271 | Non-alcoholic fatty liver disease histology (lobular) |
| rs4145170 | Cognitive performance |
| rs1941184 | Parkinson's disease (age of onset) |
| rs10438933 | Amyotrophic lateral sclerosis |

| | |
|---|---|
| rs1013696 | Response to antidepressants |
| rs4799915 | Response to iloperidone treatment (QT prolongation) |
| rs2162440 | Telomere length |
| rs8085804 | Cognitive performance |
| rs991014 | Ventricular conduction |
| rs2243803 | Menarche (age at onset) |
| rs4890568 | Hematological and biochemical traits |
| rs2576037 | Personality dimensions |
| rs1398217 | Menarche (age at onset) |
| rs4939827 | Colorectal cancer |
| rs8099594 | Height |
| rs7240405 | HDL cholesterol |
| rs4939883 | HDL cholesterol,Cholesterol, total,Lipid metabolism phenotypes |
| rs2156552 | HDL cholesterol |
| rs1036935 | Chronic lymphocytic leukemia |
| rs732528 | Entorhinal cortical thickness |
| rs17594526 | Schizophrenia |
| rs9960767 | Schizophrenia |
| rs613872 | Fuchs's corneal dystrophy |
| rs1978503 | Breast cancer |
| rs12604483 | HIV-1 susceptibility |
| rs1120787 | HIV-1 control |
| rs7236632 | Attention deficit hyperactivity disorder and conduct disorder |
| rs10503019 | Vitiligo |
| rs8093763 | Bleomycin sensitivity |
| rs12957347 | Metabolic syndrome |
| rs571312 | Body mass index |
| rs17782313 | Height,Body mass index,Obesity |
| rs10871777 | Obesity (extreme),Obesity |
| rs489693 | Antipsychotic drug-induced weight gain,Waist circumference |
| rs12970134 | Waist circumference and related phenotypes,Weight,Body mass index |
| rs4257308 | Tuberculosis |
| rs884205 | Bone mineral density,Bone mineral density (spine) |
| rs2957128 | Paget's disease |
| rs3018362 | Bone mineral density (hip),Paget's disease |
| rs470490 | Atrioventricular conduction |
| rs17077540 | Major depressive disorder |
| rs17184557 | Osteoporosis |
| rs8092443 | Response to antipsychotic treatment |
| rs11663206 | Response to antipsychotic treatment |
| rs2660917 | Iron status biomarkers |
| rs337718 | Multiple sclerosis (severity) |
| rs1943816 | P-tau181p |
| rs2717128 | RR interval (heart rate) |
| rs2002842 | Rheumatoid arthritis |
| rs4799088 | Amyotrophic lateral sclerosis |
| rs12986413 | Height |

| | |
|---|---|
| rs11669592 | Non-alcoholic fatty liver disease histology (other) |
| rs3760776 | N-glycan levels,Vitamin B12 levels,Tumor biomarkers |
| rs4072910 | Height |
| rs1862471 | Menarche (age at onset) |
| rs3093030 | Soluble levels of adhesion molecules |
| rs280519 | Psoriasis |
| rs1122608 | Myocardial infarction (early onset),Coronary heart disease |
| rs11668477 | LDL cholesterol |
| rs2738459 | LDL cholesterol |
| rs7253363 | Multiple sclerosis (severity) |
| rs7255045 | Mean corpuscular volume |
| rs11085824 | Mean corpuscular hemoglobin |
| rs11666377 | Brain lesion load |
| rs11666579 | Response to antidepressants |
| rs12608932 | Amyotrophic lateral sclerosis |
| rs7248363 | Bipolar disorder and schizophrenia |
| rs16996148 | Triglycerides,LDL cholesterol |
| rs17216525 | Triglycerides |
| rs2562456 | Pain |
| rs1975174 | Telomere length |
| rs8102137 | Bladder cancer |
| rs746961 | Select biomarker traits |
| rs1078373 | Cognitive performance |
| rs4805834 | Creatinine levels |
| rs10411210 | Colorectal cancer |
| rs736289 | Crohn's disease |
| rs29941 | Weight,Body mass index |
| rs11084753 | Body mass index |
| rs10407640 | Functional MRI |
| rs8102476 | Prostate cancer |
| rs12979860 | Response to hepatitis C treatment,Chronic Hepatitis C infection |
| rs8099917 | Response to hepatitis C treatment,Chronic Hepatitis C infection |
| rs3733829 | Smoking behavior |
| rs4105144 | Smoking behavior |
| rs7260329 | Smoking behavior |
| rs17318596 | Height |
| rs2191566 | Acute lymphoblastic leukemia (childhood) |
| rs2061333 | Alzheimer's disease |
| rs644148 | Personality dimensions |
| rs157580 | HDL cholesterol,Alzheimer's disease,Alzheimer's disease biomarkers,LDL cholesterol |
| rs439401 | HDL Cholesterol - Triglycerides (HDLC-TG),Triglycerides,Alzheimer's disease biomarkers |

| | |
|---|---|
| rs41377151 | Age-related macular degeneration,Quantitative traits,HDL cholesterol,Alzheimer's disease (late onset),Alzheimer's disease (age of onset),Longevity,Triglycerides,C-reactive protein,Alzheimer's disease,Cholesterol, total,Cognitive decline,LDL cholesterol,Lipoprotein-associated phospholipase A2 activity and mass |
| rs2159324 | Quantitative traits |
| rs11083846 | Chronic lymphocytic leukemia |
| rs11668878 | Chronic lymphocytic leukemia |
| rs281379 | Crohn's disease,Pubertal anthropometrics |
| rs2287921 | Bipolar disorder,Retinal vascular caliber |
| rs2280401 | Metabolite levels,Serum albumin level,Serum total protein level,Hematological and biochemical traits |
| rs3745516 | Primary biliary cirrhosis |
| rs266849 | Prostate-specific antigen levels |
| rs2735839 | Prostate-specific antigen levels,Prostate cancer |
| rs3826656 | Alzheimer's disease |
| rs1878047 | Body mass index |
| rs299175 | Multiple sclerosis (severity) |
| rs260461 | Attention deficit hyperactivity disorder |
| rs6051520 | Information processing speed |
| rs397020 | Multiple sclerosis |
| rs2281808 | Type 1 diabetes |
| rs6136489 | Mean platelet volume |
| rs1810636 | Low-density lipoprotein cholesterol |
| rs3761218 | Bipolar disorder |
| rs6052699 | Platelet aggregation |
| rs261360 | Hair morphology |
| rs4815868 | Information processing speed |
| rs236114 | Menopause (age at onset) |
| rs2326679 | Menopause (age at onset) |
| rs967417 | Height |
| rs2145270 | Height,Body mass index |
| rs6085920 | Uric acid levels |
| rs6118083 | Cognitive performance |
| rs6056209 | Cognitive performance |
| rs2273061 | Bone mineral density |
| rs1884136 | Information processing speed |
| rs2207418 | Cardiac hypertrophy |
| rs2073233 | Brain structure |
| rs680379 | Sphingolipid levels |
| rs1223271 | Parkinson's disease |
| rs6110278 | Mean platelet volume |
| rs4141463 | Autism |
| rs2208059 | Ileal carcinoids |
| rs852069 | Menarche (age at onset) |
| rs3790268 | Matrix metalloproteinase levels |
| rs6046396 | Bipolar disorder and schizophrenia |

| | |
|---|---|
| rs2180439 | Male-pattern baldness |
| rs1160312 | Male-pattern baldness |
| rs1158167 | Cystatin C |
| rs911119 | Chronic kidney disease |
| rs6138150 | Response to TNF antagonist treatment |
| rs291671 | Hair color |
| rs7274811 | Height |
| rs4911414 | Tanning,Skin sensitivity to sun,Freckles,Red vs. non-red hair color,Burning and freckling |
| rs1015362 | Skin sensitivity to sun,Freckles,Red vs. non-red hair color,Burning and freckling |
| rs619865 | Freckling |
| rs6060369 | Height |
| rs6060373 | Height,Spine bone size |
| rs6088813 | Height |
| rs2236164 | Height |
| rs6031882 | Hippocampal atrophy |
| rs4811196 | Bone mineral density |
| rs6024905 | Bipolar disorder and schizophrenia |
| rs6102059 | LDL cholesterol |
| rs6017342 | Ulcerative colitis |
| rs1008953 | Psoriasis |
| rs11696501 | Brain structure |
| rs6074022 | Multiple sclerosis |
| rs4810485 | Rheumatoid arthritis |
| rs6066084 | Quantitative traits |
| rs4810685 | Attention deficit hyperactivity disorder (time to onset) |
| rs13038095 | Atrial fibrillation |
| rs6063312 | Tonometry |
| rs2235617 | Psoriasis |
| rs6068020 | Male infertility |
| rs6013382 | Amyotrophic lateral sclerosis |
| rs6013509 | Hemoglobin |
| rs6091737 | Calcium levels |
| rs6127921 | Response to citalopram treatment |
| rs4811971 | Height |
| rs127430 | Cardiovascular disease risk factors |
| rs13831 | Event-related brain oscillations |
| rs16982520 | Hypertension |
| rs6027755 | Non-alcoholic fatty liver disease histology (other) |
| rs1970546 | Volumetric brain MRI |
| rs4925189 | T-tau |
| rs4925386 | Colorectal cancer |
| rs6010620 | Glioma,Glioma (high-grade) |
| rs4809324 | Glioma (high-grade) |
| rs2297441 | Ulcerative colitis |
| rs2315008 | Inflammatory bowel disease |
| rs4809330 | Crohn's disease |

| | |
|---|---|
| rs1006899 | Bone mineral density (spine) |
| rs1736135 | Crohn's disease,Ulcerative colitis |
| rs1297265 | Ulcerative colitis |
| rs2823819 | Attention deficit hyperactivity disorder |
| rs2823962 | Amyotrophic lateral sclerosis |
| rs2825388 | Attention deficit hyperactivity disorder symptoms (interaction) |
| rs1888414 | Hippocampal atrophy |
| rs2828520 | Major depressive disorder |
| rs9977253 | Non-alcoholic fatty liver disease histology (other) |
| rs17001239 | Cognitive performance |
| rs2830840 | Response to citalopram treatment |
| rs239713 | AB1-42 |
| rs9305354 | Urinary albumin excretion |
| rs363512 | Hyperactive-impulsive symptoms |
| rs9305406 | Response to statin therapy |
| rs7283316 | Information processing speed |
| rs13048019 | Amyotrophic lateral sclerosis |
| rs2833556 | Reasoning |
| rs2833607 | Vitiligo |
| rs1003719 | Eye color traits |
| rs2835810 | Metabolic syndrome |
| rs743446 | Neonatal lupus |
| rs2836823 | Nicotine dependence |
| rs2242944 | Ankylosing spondylitis |
| rs9981861 | Non-small cell lung cancer |
| rs7279297 | Tanning |
| rs425215 | Common traits (Other) |
| rs11203203 | Type 1 diabetes,Celiac disease and Rheumatoid arthritis,Rheumatoid arthritis,Vitiligo |
| rs9976767 | Type 1 diabetes |
| rs2839619 | Biochemical measures |
| rs234720 | Cognitive performance |
| rs496300 | Metabolic syndrome |
| rs2838519 | Crohn's disease,Ulcerative colitis |
| rs762421 | Crohn's disease |
| rs2838815 | Pulmonary function |
| rs131654 | Systemic lupus erythematosus |
| rs181359 | Crohn's disease |
| rs4821112 | Mean corpuscular volume |
| rs5751614 | Height |
| rs4820599 | Liver enzyme levels,Gamma glutamyl transpeptidase |
| rs5751901 | Protein quantitative trait loci |
| rs8139900 | Uric acid levels |
| rs688034 | Coronary heart disease |
| rs1547014 | Vertical cup-disc ratio |
| rs738722 | Esophageal cancer and gastric cancer |
| rs2412973 | Inflammatory bowel disease (early onset) |

| | |
|---|---|
| rs5753037 | Type 1 diabetes |
| rs713875 | Crohn's disease |
| rs2106294 | Type 2 diabetes nephropathy |
| rs9609565 | Hematological parameters |
| rs130575 | Attention deficit hyperactivity disorder |
| rs4821469 | End-stage renal disease (non-diabetic) |
| rs735854 | Optic disc size (rim) |
| rs5756506 | Hematological parameters |
| rs2413450 | Mean corpuscular hemoglobin,Mean corpuscular volume,Hematocrit,Hematological and biochemical traits |
| rs229541 | Type 1 diabetes |
| rs9607469 | Optic disc parameters |
| rs2284063 | Cutaneous nevi,Melanoma |
| rs1014971 | Bladder cancer |
| rs9623117 | Prostate cancer |
| rs139909 | Height |
| rs7364180 | Alzheimer's disease biomarkers |
| rs5759167 | Prostate cancer |
| rs2281135 | Liver enzyme levels |
| rs9615362 | Low-density lipoprotein cholesterol |
| rs6009824 | Natriuretic peptide levels |
| rs470119 | Mean corpuscular hemoglobin |
| rs131794 | Mean corpuscular volume |
| rs7885458 | Biochemical measures |
| rs7892812 | Cognitive performance |
| rs12388359 | Alcohol dependence |
| rs5934953 | Cognitive performance |
| rs5979785 | Celiac disease |
| rs5925696 | Erectile dysfunction and prostate cancer treatment |
| rs5944185 | Erectile dysfunction and prostate cancer treatment |
| rs5971305 | Erectile dysfunction and prostate cancer treatment |
| rs6611365 | Optic disc size (disc) |
| rs1934179 | Hypospadias |
| rs1327301 | Prostate cancer |
| rs5945572 | Prostate cancer |
| rs5945619 | Prostate cancer |
| rs5965182 | Erectile dysfunction and prostate cancer treatment |
| rs6625163 | Male-pattern baldness |
| rs4844096 | Primary tooth development (number of teeth) |
| rs5936487 | Primary tooth development (time to first tooth eruption) |
| rs1474563 | Height |
| rs2710057 | Bone mineral density (hip) |
| rs5941436 | Cognitive performance |
| rs75900375 | Alzheimer's disease |
| rs5982533 | Biochemical measures |
| rs2430212 | Iron status biomarkers |
| rs5955415 | Bipolar disorder and schizophrenia |

| | |
|---|---|
| rs6627057 | Bipolar disorder and schizophrenia |
| rs5936441 | Response to acetaminophen (hepatotoxicity) |
| rs2159767 | Schizophrenia |
| rs530501 | Cognitive performance |
| rs5945326 | Type 2 diabetes |
| rs766420 | Bilirubin levels |
| rs2664170 | Type 1 diabetes |
