# Supplementary material for: Genome at Juncture of Early Human Migration: A Systematic Analysis of Two Whole Genomes and Thirteen Exomes from Kuwaiti Population Subgroup of Inferred Saudi Arabian Tribe Ancestry
Source: PLoS One. 2014 Jun 4;9(6):e99069. doi: 10.1371/journal.pone.0099069 (PMC4045902; doi:10.1371/journal.pone.0099069)
Supplement: Table S4 — Structural variations as seen in the two whole genome sequences of Saudi Arabian tribe ancestry. (PDF) [file pone.0099069.s008.pdf]

Type of Variations	KWS1	KWS2	Consensus
Deletions	15950	14921	27060
Duplications	1985	2253	4192
Insertions	654	390	1044
Inversions	591	627	1137
Tandem Duplicates	1981	1995	3411
Translocations (Intra-chromosomal)	641	577	1216
Translocations (Inter-Chromosomal)	704	998	1702
