# Supplementary material for: Genome at Juncture of Early Human Migration: A Systematic Analysis of Two Whole Genomes and Thirteen Exomes from Kuwaiti Population Subgroup of Inferred Saudi Arabian Tribe Ancestry
Source: PLoS One. 2014 Jun 4;9(6):e99069. doi: 10.1371/journal.pone.0099069 (PMC4045902; doi:10.1371/journal.pone.0099069)
Supplement: Table S5 — List of the 164 deleterious variants that show significant differences in allele frequencies between KWS samples and continental populations. (PDF) [file pone.0099069.s009.pdf]

SNP	Reference	Alternates	AA Ref	AA Alt	CDS Strand	Ref Codon	Codon Pos	Ancestral Allele	SIFT	SIFT Pred	PolyPhen2 HumVar	PolyPhen2 HumVar Pred
rs138288952	G	A	G	S	+	GGC	1	G	0.51	Tolerated	0.949	Probably Damaging
rs114987153	C	T	A	V	+	GCG	2	C	0.05	Damaging	0	Benign
rs61739207	C	T	T	I	+	ACA	2	C	0.35	Tolerated	0.986	Probably Damaging
rs61742849	G	A	G	D	+	GGC	2	G	0.01	Damaging	0.555	Possibly Damaging
rs146993928	C	A	S	I	-	AGC	2	C	0	Damaging	0.999	Probably Damaging
rs144766457	C	T	R	W	+	CGG	1	c	0.03	Damaging	0.895	Possibly Damaging
rs141557248	C	T	P	S	+	CCA	1	C	0.01	Damaging	0.013	Benign
rs112218133	C	T	H	Y	+	CAT	1	C	0	Damaging	0.537	Possibly Damaging
rs138142057	G	C	D	H	+	GAC	1	G	0	Damaging	0.818	Possibly Damaging
rs74967568	G	A	G	S	+	GGC	1	G	0.01	Damaging	0.596	Possibly Damaging
rs149727487	T	A	L	Q	+	CTG	2	T	0	Damaging	0.956	Probably Damaging
rs72762066	C	T	P	L	+	CCC	2	C	0.01	Damaging	0.42	Benign
rs140374909	C	T	R	H	-	CGC	2	C	0.05	Damaging	0.219	Benign
rs144891600	T	C	M	V	-	ATG	1	T	0.04	Damaging	0.014	Benign
rs7542813	A	G	T	A	+	ACG	1	G	0.01	Damaging	0	Benign
rs78622	C	T	G	D	-	GGC	2	?	0.05	Dama	0	Benign

116										ging		
rs2175977	G	C	S	T	+	AGC	2	G	0.05	Damaging	0.965	Probably Damaging
rs72819557	A	C	Q	H	+	CAA	3	A	0.16	Tolerated	0.99	Probably Damaging
rs17036091	T	C	Y	H	+	TAT	1	T	0	Damaging	1	Probably Damaging
rs143400009	T	C	S	P	+	TCC	1	T	0.06	Tolerated	0.998	Probably Damaging
rs16853333	G	A	G	E	+	GGA	2	G	0.01	Damaging	1	Probably Damaging
rs146057575	T	C	N	S	-	AAT	2	T	0	Damaging	0.001	Benign
rs151183191	A	C	S	R	-	AGT	3	A	0.04	Damaging	0.656	Possibly Damaging
rs1626450	A	G	I	V	+	ATA	1	G	0.02	Damaging	0.027	Benign
rs199834849	A	T	L	H	-	CTT	2	A	0	Damaging	0.998	Probably Damaging
rs140144664	C	T	P	S	+	CCC	1	C	0.04	Damaging	0.998	Probably Damaging
rs6707568	A	G	L	P	-	CTG	2	G	0	Damaging	0	Benign
rs2923857	G	C	A	P	+	GCC	1	C	0	Damaging	0	Benign
rs61749309	T	C	R	G	-	AGA	1	T	0.03	Damaging	0.009	Benign
rs150362720	T	A	N	Y	-	AAT	1	T	0.01	Damaging	0.177	Benign
rs148929708	A	G	N	D	+	AAT	1	A	0.85	Tolerated	0.931	Probably Damaging
rs35261777	A	G	I	M	+	ATA	3	A	0.01	Damaging	0.243	Benign

rs138288586	A	G	T	A	+	ACT	1	A	0.01	Damaging	0.998	Probably Damaging
rs147017058	C	G	S	R	+	AGC	3	C	0.37	Tolerated	0.965	Probably Damaging
rs149515968	T	A	S	T	+	TCC	1	T	0.46	Tolerated	0.998	Probably Damaging
rs116030393	T	C	V	A	+	GTC	2	T	0.01	Damaging	0.411	Benign
rs146859876	G	A	G	D	+	GGC	2	G	0.47	Tolerated	1	Probably Damaging
rs62282468	G	T	P	H	-	CCT	2	g	?	?	0.98	Probably Damaging
rs1134986	C	T	R	Q	-	CGA	2	C	0.15	Tolerated	0.955	Probably Damaging
rs150738695	A	G	C	R	-	TGT	1	A	0	Damaging	1	Probably Damaging
rs28522910	C	T	P	L	+	CCG	2	C	0.01	Damaging	0.035	Benign
rs78833872	C	T	P	L	+	CCG	2	C	0	Damaging	0.142	Benign
rs6850206	C	T	V	M	-	GTG	1	C	0.1	Tolerated	0.977	Probably Damaging
rs72552705	G	C	C	S	+	TGC	2	-	0	Damaging	0.003	Benign
rs145319675	G	T	Q	K	-	CAG	1	G	0.01	Damaging	0.879	Possibly Damaging
rs149692526	G	A	R	Q	+	CGA	2	G	0.13	Tolerated	0.997	Probably Damaging
rs150680111	C	T	T	M	+	ACG	2	C	0.02	Damaging	0.965	Probably Damaging
rs75762896	T	G	F	C	+	TTC	2	T	0.02	Damaging	0.586	Possibly Damaging
rs74512322	G	A	L	F	-	CTC	1	G	0.04	Damaging	0.191	Benign

rs202132393	C	T	G	S	-	GGT	1	C	0	Damaging	1	Probably Damaging
rs72706355	G	A	R	Q	+	CGA	2	G	0	Damaging	0.996	Probably Damaging
rs35433301	C	T	R	W	+	CGG	1	C	0	Damaging	0.998	Probably Damaging
rs34748216	C	T	S	F	+	TCC	2	C	0.04	Damaging	0.311	Benign
rs200055351	T	C	V	A	+	GTG	2	T	0	Damaging	0.249	Benign
rs111363347	C	T	V	I	-	GTC	1	C	0.01	Damaging	0.973	Probably Damaging
rs17408150	T	A	L	Q	+	CTA	2	T	0	Damaging	0.985	Probably Damaging
rs34390520	C	T	L	F	+	CTC	1	C	0.05	Damaging	0.352	Benign
rs141134077	G	C	G	A	+	GGG	2	?	0.1	Tolerated	1	Probably Damaging
rs140080192	G	A	E	K	+	GAG	1	?	0	Damaging	0.999	Probably Damaging
rs112243146	G	A	G	E	+	GGG	2	G	0.02	Damaging	?	?
rs9256983	A	T	R	W	+	AGG	1	?	0	Damaging	?	?
rs144031629	A	G	H	R	+	CAT	2	A	0	Damaging	0.999	Probably Damaging
rs138769310	C	A	V	L	-	GTG	1	C	0	Damaging	0.021	Benign
rs143669522	G	A	T	I	-	ACC	2	G	0.05	Damaging	0.082	Benign
rs148443947	C	T	S	L	+	TCG	2	C	0	Damaging	0.953	Probably Damaging
rs145779681	C	T	P	L	+	CCG	2	C	0.17	Tolerated	0.993	Probably Damaging

rs35504966	G	A	L	F	-	CTT	1	G	0.05	Damaging	0.07	Benign
rs147800155	G	A	R	Q	+	CGG	2	G	0	Damaging	0.918	Probably Damaging
rs72657315	A	G	T	A	+	ACT	1	A	0.03	Damaging	0.028	Benign
rs61756062	A	C	I	S	-	ATC	2	A	0.01	Damaging	0.956	Probably Damaging
rs113016282	A	G	I	T	-	ATC	2	A	0	Damaging	0.97	Probably Damaging
rs145722885	C	T	R	Q	-	CGG	2	C	0.03	Damaging	0.511	Possibly Damaging
rs149360770	T	C	K	R	-	AAA	2	T	0	Damaging	0.275	Benign
rs190333133	C	T	R	W	+	CGG	1	C	0	Damaging	1	Probably Damaging
rs2234013	G	A	G	S	+	GGT	1	G	0	Damaging	0.997	Probably Damaging
rs150441443	G	A	R	H	+	CGT	2	G	0.04	Damaging	0.189	Benign
rs149140637	A	G	L	P	-	CTA	2	A	0	Damaging	0.997	Probably Damaging
rs117851304	G	A	R	H	+	CGC	2	G	0	Damaging	0.836	Possibly Damaging
rs17856355	C	G	L	V	+	CTG	1	C	0.05	Damaging	0.996	Probably Damaging
rs149106647	T	G	I	M	+	ATT	3	T	0	Damaging	0.983	Probably Damaging
rs141421747	C	G	L	V	+	CTG	1	c	0	Damaging	?	?
rs147275962	C	T	V	M	-	GTG	1	C	0	Damaging	0.677	Possibly Damaging
rs138686336	G	A	R	C	-	CGC	1	G	0.02	Damaging	0.954	Probably Damaging

rs200510870	G	A	L	F	-	CTC	1	G	0	Damaging	0.028	Benign
rs137868712	G	A	R	C	-	CGC	1	G	0	Damaging	0.917	Probably Damaging
rs28671254	G	A	R	C	-	CGC	1	g	0.01	Damaging	0.877	Possibly Damaging
rs146112401	G	A	V	M	+	GTG	1	G	0.01	Damaging	0.067	Benign
rs7076156	A	G	T	A	+	ACC	1	G	0	Damaging	0.001	Benign
rs139881253	T	A	T	S	-	ACC	1	T	0.02	Damaging	0.472	Possibly Damaging
rs2231687	A	G	F	L	-	TTT	1	A	0.05	Damaging	0	Benign
rs150893869	A	G	K	E	+	AAA	1	A	0	Damaging	0.761	Possibly Damaging
rs10776692	C	G	R	G	+	CGC	1	G	0	Damaging	0	Benign
rs112269777	C	T	G	R	-	GGG	1	C	0	Damaging	?	?
rs192473731	G	A	V	I	+	GTC	1	G	0	Damaging	?	?
rs7077	G	C	G	A	+	GGG	2	g	0	Damaging	?	?
rs151296695	T	C	V	A	+	GTA	2	T	0	Damaging	0.245	Benign
rs145474756	G	T	L	M	-	CTG	1	G	0.01	Damaging	0.982	Probably Damaging
rs144873026	G	C	P	R	-	CCT	2	G	0	Damaging	?	?
rs149447683	C	T	V	M	-	GTG	1	C	0.03	Damaging	0.055	Benign
rs45511502	C	T	E	K	-	GAG	1	C	0	Damaging	0.067	Benign

rs140432356	C	T	S	F	+	TCT	2	C	0.04	Damaging	0.998	Probably Damaging
rs61753359	G	A	V	M	+	GTG	1	G	0.05	Damaging	0.921	Probably Damaging
rs138705814	A	G	I	V	+	ATA	1	A	0	Damaging	?	?
rs139979211	C	T	T	M	+	ACG	2	C	0	Damaging	?	?
rs5745068	C	T	R	H	-	CGC	2	C	0.02	Damaging	0.652	Possibly Damaging
rs75008557	A	G	N	S	+	AAT	2	?	0.02	Damaging	0.732	Possibly Damaging
rs140239552	C	T	V	M	-	GTG	1	C	0	Damaging	0.995	Probably Damaging
rs35650607	C	T	V	M	-	GTG	1	C	0.11	Tolerated	0.969	Probably Damaging
rs141259390	G	A	T	I	-	ACA	2	G	0.06	Tolerated	0.931	Probably Damaging
rs142908361	T	C	R	G	-	AGG	1	T	0	Damaging	0.051	Benign
rs78193368	T	C	I	T	+	ATT	2	T	0	Damaging	0.977	Probably Damaging
rs117095293	A	C	S	A	-	TCT	1	A	0.11	Tolerated	0.997	Probably Damaging
rs4987133	A	G	I	T	-	ATT	2	A	0	Damaging	0.735	Possibly Damaging
rs61747605	G	A	P	S	-	CCT	1	G	0.06	Tolerated	0.993	Probably Damaging
rs55739947	C	A	L	M	+	CTG	1	C	0.02	Damaging	1	Probably Damaging
rs1800067	G	A	R	Q	+	CGA	2	G	0.01	Damaging	0.98	Probably Damaging
rs151258803	C	T	R	K	-	AGA	2	C	0.04	Damaging	0.996	Probably Damaging

rs147727760	C	G	R	P	-	CGA	2	?	0.02	Damaging	0.631	Possibly Damaging
rs183146864	G	A	V	I	+	GTA	1	G	0	Damaging	0.993	Probably Damaging
rs140699573	C	A	Q	H	-	CAG	3	C	0.29	Tolerated	0.996	Probably Damaging
rs141218390	C	A	A	E	+	GCG	2	C	0.05	Damaging	0.056	Benign
rs149163440	C	G	P	R	+	CCC	2	C	0	Damaging	0.316	Benign
rs56014026	G	A	T	M	-	ACG	2	G	0.02	Damaging	0.965	Probably Damaging
rs62624978	C	G	G	A	-	GGG	2	c	0.87	Tolerated	0.999	Probably Damaging
rs61753153	C	T	A	T	-	GCT	1	C	0.04	Damaging	0.871	Possibly Damaging
rs78909145	T	C	K	R	-	AAG	2	T	0.02	Damaging	0.06	Benign
rs11658194	T	C	S	P	+	TCG	1	C	0.01	Damaging	?	?
rs63491460	C	G	S	W	+	TCG	2	G	0	Damaging	?	?
rs144643694	G	T	A	D	-	GCT	2	G	0.02	Damaging	0.143	Benign
rs887230	C	T	R	K	-	AGG	2	T	0	Damaging	0	Benign
rs139865124	T	A	E	V	-	GAG	2	T	0	Damaging	0.994	Probably Damaging
rs147050513	C	T	R	Q	-	CGA	2	C	0.03	Damaging	0.999	Probably Damaging
rs7226498	T	C	R	G	-	AGA	1	C	0	Damaging	?	?
rs61733594	C	T	G	R	-	GGG	1	C	0	Damaging	0.844	Possibly Damaging

rs182872936	C	T	T	M	+	ACG	2	C	0	Damaging	0.994	Probably Damaging
rs138288250	A	G	Y	C	+	TAT	2	A	0	Damaging	0.514	Possibly Damaging
rs193234359	C	T	P	L	+	CCC	2	C	0	Damaging	?	?
rs62106065	T	G	C	G	+	TGT	1	?	0.5	Tolerated	0.984	Probably Damaging
rs62106066	T	C	L	P	+	CTC	2	?	0	Damaging	0.976	Probably Damaging
rs2108622	C	T	V	M	-	GTG	1	c	0.01	Damaging	0.98	Probably Damaging
rs189646827	G	T	E	D	+	GAG	3	-	0.04	Damaging	0.89	Possibly Damaging
rs143821677	G	A	T	M	-	ACG	2	G	0	Damaging	0.996	Probably Damaging
rs148996461	G	A	A	V	-	GCG	2	G	0	Damaging	0.886	Possibly Damaging
rs56084716	C	G	R	G	+	CGA	1	C	0.02	Damaging	0.994	Probably Damaging
rs8192709	C	T	R	C	+	CGC	1	C	0	Damaging	0.265	Benign
rs76853904	C	T	G	R	-	GGA	1	C	0.05	Damaging	0.568	Possibly Damaging
rs145131242	T	G	F	C	+	TTC	2	T	0.01	Damaging	0.954	Probably Damaging
rs28399656	T	A	M	K	+	ATG	2	?	0.02	Damaging	0.159	Benign
rs139657571	A	G	M	T	-	ATG	2	a	0	Damaging	0.835	Possibly Damaging
rs61978639	G	C	G	A	+	GGC	2	G	0	Damaging	0.801	Possibly Damaging
rs118147154	A	C	Q	P	+	CAG	2	A	0	Damaging	0.979	Probably Damaging

rs75242867	C	G	G	R	-	GGG	1	C	0	Damaging	0.015	Benign
rs201546457	A	C	L	R	-	CTC	2	A	0	Damaging	0.787	Possibly Damaging
rs7247236	T	C	T	A	-	ACC	1	C	0	Damaging	0	Benign
rs35394887	C	A	D	E	+	GAC	3	C	0.01	Damaging	0.498	Possibly Damaging
rs61729229	T	C	W	R	+	TGG	1	T	0	Damaging	1	Probably Damaging
rs140814287	C	T	R	C	+	CGC	1	C	?	?	0.997	Probably Damaging
rs4431000	C	A	G	W	-	GGG	1	C	0.02	Damaging	0.966	Probably Damaging
rs117821416	A	T	S	C	+	AGC	1	A	0	Damaging	0.926	Probably Damaging
rs151104204	G	A	P	S	-	CCT	1	G	0	Damaging	0.932	Probably Damaging
rs80027270	G	A	T	M	-	ACG	2	G	0	Damaging	?	?
rs151338796	G	A	P	S	-	CCT	1	g	0	Damaging	?	?
rs117135869	C	T	A	V	+	GCC	2	C	0.03	Damaging	0.992	Probably Damaging
rs140861368	C	T	S	F	+	TCC	2	C	0.05	Damaging	0.879	Possibly Damaging
