# Supplementary material for: Genome at Juncture of Early Human Migration: A Systematic Analysis of Two Whole Genomes and Thirteen Exomes from Kuwaiti Population Subgroup of Inferred Saudi Arabian Tribe Ancestry
Source: PLoS One. 2014 Jun 4;9(6):e99069. doi: 10.1371/journal.pone.0099069 (PMC4045902; doi:10.1371/journal.pone.0099069)
Supplement: Table S7 — Concordance rates for SNP calls between deep sequencing experiments and genome-wide genotyping. (PDF) [file pone.0099069.s011.pdf]

Sample ID	Total SNPs Overlapping	Total Mismatch	Homozygous SNPs		Heterozygous SNPs	
			Total SNPs	SNPs with Mismatch Calls	Total SNPs	SNPs with Mismatch Calls
KWS1	311,152	193	120,048	94	191,104	99
KWS2	309,379	387	119,883	145	189,496	242
KWS3	13,249	95	5,222	6	8,027	89
KWS4	13,212	78	5,426	12	7,786	66
KWS5	13,198	51	5,103	10	8,095	41
KWS6	12,781	72	5,820	11	6,961	61
KWS7	13,247	84	5,267	16	7,980	68
KWS8	13,231	74	5,913	12	7,318	62
KWS9	13,096	78	5,553	13	7,543	65
KWS10	13,461	64	5,638	6	7,823	58
KWS11	13,205	65	5,572	9	7,633	56
KWS12	12,725	132	5,363	27	7,362	105
KWS13	13,171	69	5,483	16	7,688	53
KWS14	13,131	86	5,392	7	7,739	79
KWS15	13,063	104	5,360	10	7,703	94
