# Supplementary material for: Genome at Juncture of Early Human Migration: A Systematic Analysis of Two Whole Genomes and Thirteen Exomes from Kuwaiti Population Subgroup of Inferred Saudi Arabian Tribe Ancestry
Source: PLoS One. 2014 Jun 4;9(6):e99069. doi: 10.1371/journal.pone.0099069 (PMC4045902; doi:10.1371/journal.pone.0099069)
Supplement: Table S8 — Details about genome sequences used to compare diversity of KWS genomes. (PDF) [file pone.0099069.s012.pdf]

Individual ID	Population	Ethnicity	Platform	Reference
NA19240	YRI	African	ABI SOLiD	De la Vega et al. [5]
NA18507	YRI	African	ABI SOLiD	Bentley et al. [6]
NA18507	YRI	African	Illumina	Mc Kernan et al. [7]
Chinese	Han Chinese	Asian	Illumina	Wang et al. [8]
Korean	Korean	Asian	Illumina	Ahn et al. [9]
Venter	CEU	Europe	Sanger	Levy et al. [10]
Watson	CEU	Europe	Roche 454	Wheeler et al. [11]
NA07022	CEU	Europe	CGenomics	Drmanac et al. [12]
NA12878	CEU	Europe	ABI SOLiD	De la Vega et al. [5]
Quake	CEU	Europe	Helicos	Pushkarev et al. [13]
NA19701	ASW	African	CGenomics	Drmanac et al. [12]
NA06985	CEU	Europe	CGenomics	Drmanac et al. [12]
NA18537	CHB	Asian	CGenomics	Drmanac et al. [12]
NA20845	GIH	Asian	CGenomics	Drmanac et al. [12]
NA18956	JPT	Asian	CGenomics	Drmanac et al. [12]
NA19020	LWK	African	CGenomics	Drmanac et al. [12]
NA21767	MKK	African	CGenomics	Drmanac et al. [12]
NA19670	MXL	American	CGenomics	Drmanac et al. [12]
NA20509	TSI	Europe	CGenomics	Drmanac et al. [12]
NA19129	YRI	African	CGenomics	Drmanac et al. [12]
